# Efficient synthesis of pyrazolopyridines containing a chromane backbone through domino reaction

**DOI:** 10.3762/bjoc.15.85

**Published:** 2019-04-11

**Authors:** Razieh Navari, Saeed Balalaie, Saber Mehrparvar, Fatemeh Darvish, Frank Rominger, Fatima Hamdan, Sattar Mirzaie

**Affiliations:** 1 Peptide Chemistry Research Center, K. N. Toosi University of Technology, P. O. Box 15875-4416, Tehran, Iran, Tel: +98-21-23064226, Fax: +98-21-22889403; 2 Medical Biology Research Center, Kermanshah University of Medical Sciences, Kermanshah, Iran; 3 Organisch-Chemisches Institut der Universitaet Heidelberg, Im Neuenheimer Feld 270, D-69120 Heidelberg, Germany

**Keywords:** chromane, domino reaction, fused heterocyclic skeletons, pyrazolopyridines

## Abstract

An efficient approach for the synthesis of pyrazolopyridines containing the aminochromane motif through a base-catalyzed cyclization reaction is reported. The synthesis was carried out through a three-component reaction of (arylhydrazono)methyl-4*H*-chromen-4-one, malononitrile, primary amines in the presence of Et_3_N at room temperature. However, carrying out the reaction under the same conditions without base led to a fused chromanyl-cyanopyridine. High selectivity, high atom economy, and good to high yields in addition to mild reaction conditions are the advantages of this approach.

## Introduction

The synthesis of new fused heterocyclic backbones has always been a major challenge in the field of organic synthesis [1–3]. Multicomponent reactions (MCRs) and domino reactions are known as efficient synthetic approaches for the synthesis of complex molecules [4–6]. Domino reactions have been defined by Tietze and were applied for the synthesis of complicated scaffolds [7–9]. The selection of a suitable starting material and designing a post-transformational reaction is a key point for the synthesis of complex molecules through the designing of domino reactions [10].

Chromone derivatives have been found to exhibit a broad range of biological activities [11–13]. 3-Formylchromone was used as a suitable starting material for the construction of various heterocyclic skeletons [14–17].

**Figure 1:**
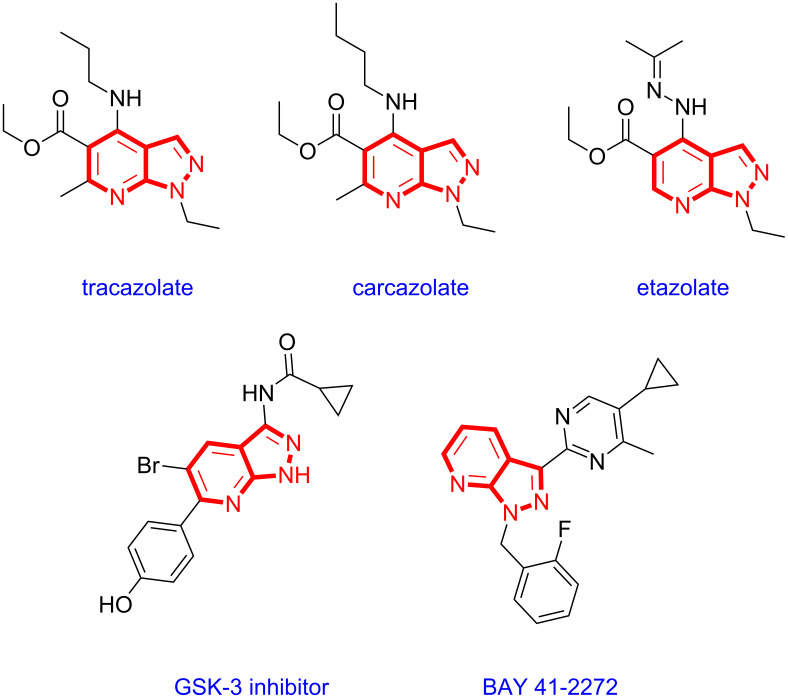
Selected examples of bioactive molecules based on the pyrazolopyridine framework.

Pyrazolopyridines are a promising class of heterocyclic compounds which inhibits cyclin-dependent protein kinase-2 (cdk-2), cyclin-dependent protein kinase-5 (cdk-5), and phosphatidylinositol 3-kinase (PI3-K) [18–21]. Thus, these compounds have a high potential for the treatment of several neurological diseases. Some examples of pyrazolopyridine derivatives are anxiolytic drugs such as tracazolate, cartazolate, and etazolate [22]. Other pyrazolopyridine-containing bioactive compounds include a GSK-3 inhibitor [23] and BAY 41-2272, [24–25] and could be used as cardiovascular therapeutic agents (Figure 1). Moreover, pyrazolopyridine derivatives also have industrial importance as fluorophores and luminophores in organic light emitting diodes [26–27].

**Scheme 1:**
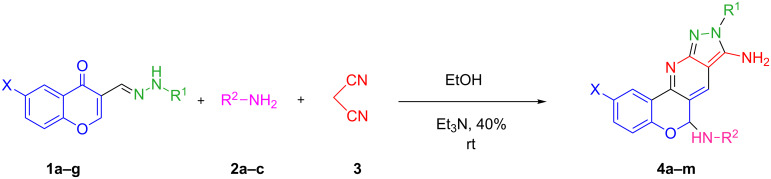
Synthesis of pyrazolopyridines containing chromone **4a**–**m** through a multicomponent reaction.

The synthesis of pyrazolopyridines has been reported in several different multistep manners [28–36]. However, some of the reported methods have several limitations such as long reaction time, low yields, boring workup procedures, and several side reactions with less selectivity of the process. Due to the extended biological activities of pyrazolopyridines, finding a suitable approach for the synthesis of these compounds is a synthetic challenge in organic synthesis. In continuation of our research work to construct fused heterocyclic skeletons [37–40], we describe the synthesis of pyrazolopyridines **4a**–**m** containing a chromone moiety (Scheme 1). The synthetic approach was based on a domino reaction of (arylhydrazono)methyl-4*H*-chromen-4-one **1a**–**g**, primary amines **2a–c**, and malononitrile (**3**) in the presence of triethylamine in ethanol at room temperature.

## Results and Discussion

In the beginning, 3-formylchromone was prepared based on a known method [41]. Then, we focused our initial study on the preparation of the (arylhydrazono)methyl-4*H*-chromen-4-one via the reaction of 3-formylchromone and phenylhydrazine [42–43]. Subjecting (arylhydrazono)methyl-4*H*-chromen-4-one (**1a**), benzylamine (**2a**), and malononitrile (**3**) in the presence of 40% triethylamine was stirred at room temperature in ethanol. Compound **4a** was precipitated and filtered. After confirmation of the structure, we tried to find a suitable base and solvent for the model reaction. The results are summarized in Table 1.

**Table 1:** Optimization of solvent and base for the synthesis of **4a** as a model reaction.

Entry	Base	Solvent	Yield^a^ (%)

**1**	**Et_3_N 40%**	**EtOH**	**65**
2	DBU 40%	EtOH	61
3	DIPEA 40%	EtOH	60
4	Et_3_N 40%	DCM	12
5	Et_3_N 40%	MeCN	35
6	Et_3_N 40%	MeOH	63

^a^The reported yields are the related amount of pure precipitate that did not require any further purification.

The reactions were studied in the presence of 10, 20, 30% triethylamine as a base in ethanol and the yields were formed 25, 38, 58%, respectively (entries 1–3, Table 1). After changing the ratio of triethylamine to 40%, the yield of pure product was increased to 65% in ethanol. The yield of the reaction in the presence of DBU and diisopropylethylamine (DIPEA) as a base were 61 and 60%, respectively. This means that the type of base does not play an effective role in this reaction. The effect of different solvents (EtOH, MeOH, MeCN, and DCM) was investigated on the progress of the reaction. As shown in Table 1, carrying out the reaction in EtOH was found the best choice due to the better yield (65%). Based on this data, the optimized reaction conditions were achieved using 40% of Et_3_N in ethanol at room temperature. The use of a protic solvent seems to be essential for the progress of the reaction and may related to the dipole moment and also the hydrogen-bonding ability of protic solvents.

**Figure 2:**
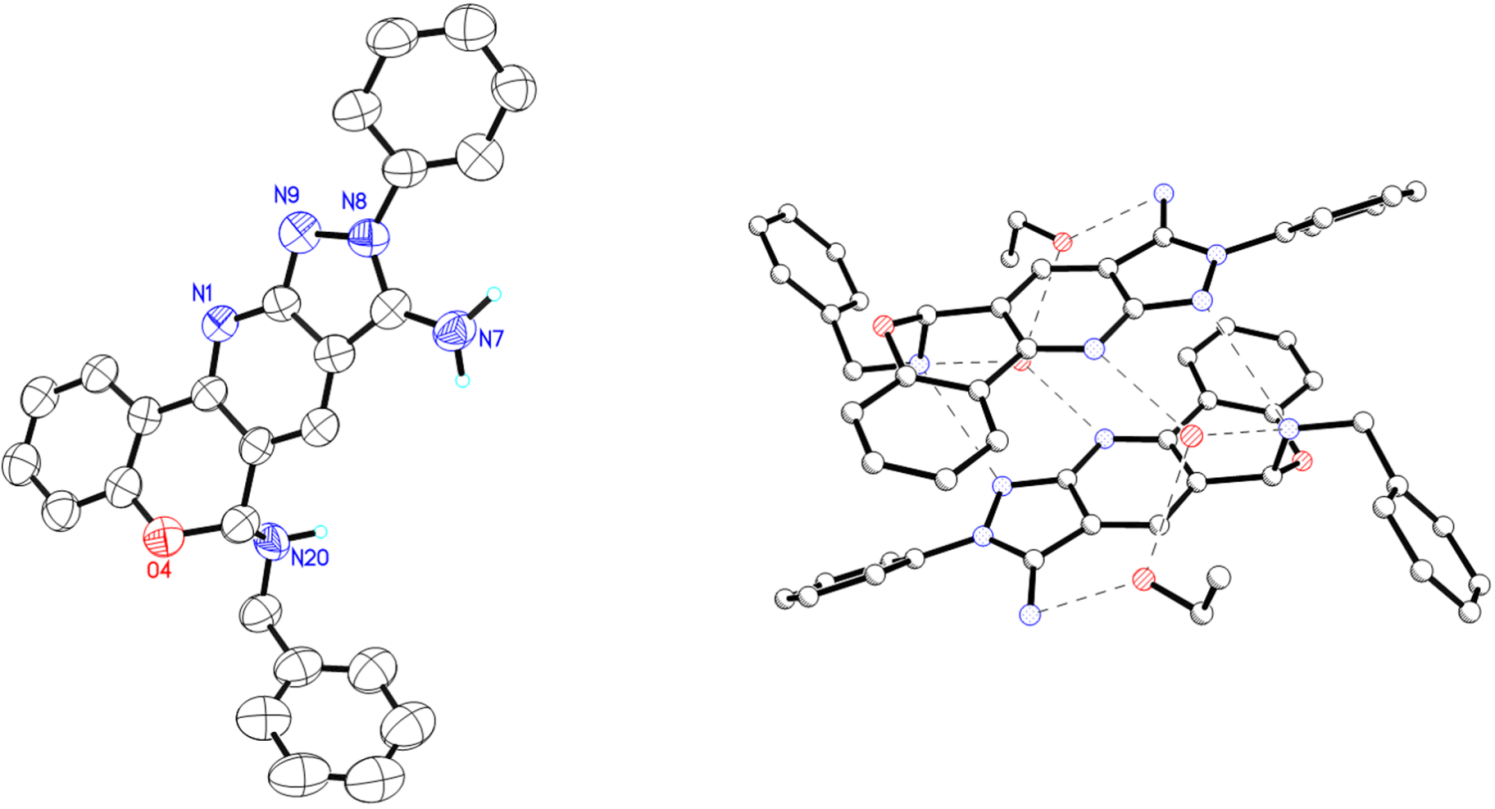
ORTEP Structure of compound **4a** and intermolecular hydrogen bonding; the ellipsoid probability level of each ORTEP diagram is 50%.

The spectroscopic data confirmed the formation of **4a**. The X-ray crystallographic data of compound **4a** confirmed the structure of the product (Figure 2). Meanwhile, via hydrogen bridges and the solvent molecules (water and ethanol) the molecules build pairs with π–π stacking of the aromatic systems.

**Figure 3:**
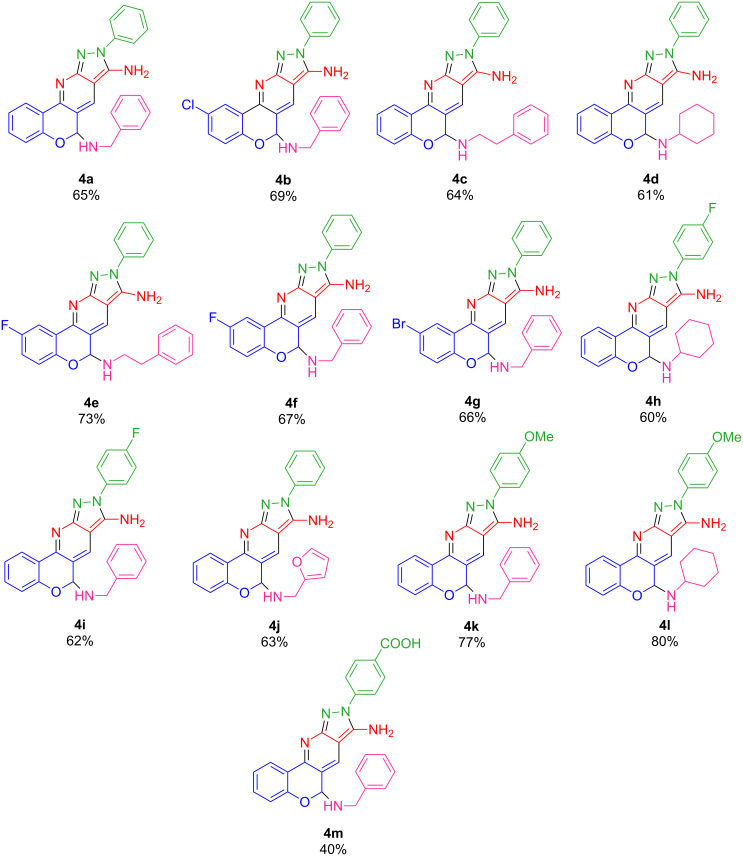
Structures of synthesized pyrazolopyridines **4a**–**m**. Reaction conditions: 3-formylchromone derivatives (1 mmol), primary amine (1.2 mmol), malononitile (1.2 mmol), Et_3_N (40 mmol %) in 3 mL ethanol at room temperature for 8–12 h.

To examine the scope and generality of our reaction, other (arylhydrazono)methyl-4*H*-chromen-4-ones were synthesized and their reaction with different primary amines and malononitrile were examined and compounds **4a**–**m** were formed. The results are summarized in Figure 3. In another experiment, the model reaction of (arylhydrazono)methyl-4*H*-chromen-4-one (**1a**), phenylethylamine (**2b**), and malononitrile (**3**) were investigated in ethanol at room temperature. The formed precipitate was collected as the sole product and the spectroscopic data were investigated and showed that the product is identified as the fused heterocyclic pyridinochromane **5a**. This confirmed that the final cyclization needs basic reaction conditions.

The synthesized (arylhydrazono)methyl-4*H*-chromen-4-ones **1a**–**g** have several electron-deficient centers such as at C-2, C-4 and C=N and are susceptible to different intramolecular and intermolecular ring formations.

**Scheme 2:**
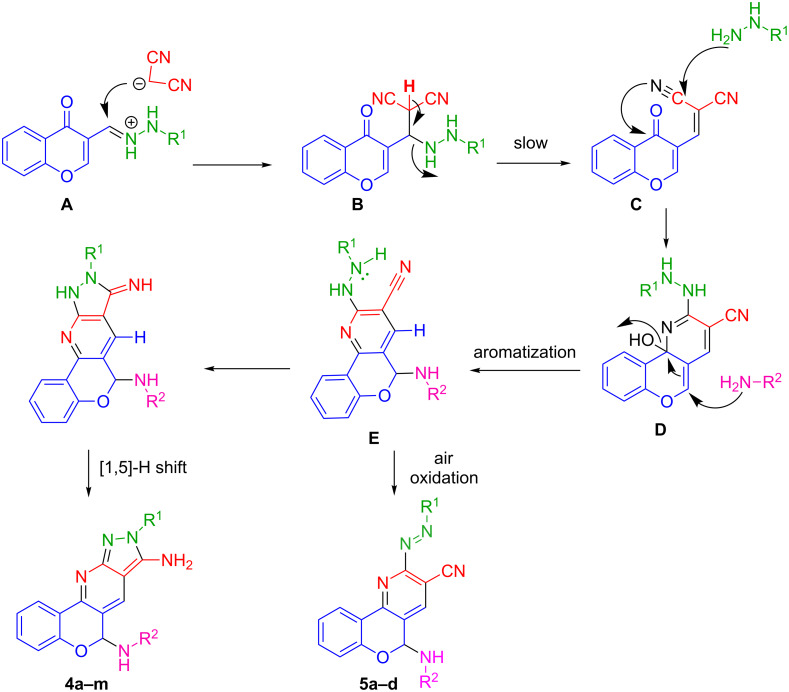
The proposed mechanism for the synthesis of pyrazolopyridines derivatives **4a**–**m** and **5a–d**.

It seems that a proposed mechanism could proceed initially through (arylhydrazono)methyl-4*H*-chromen-4-one as a suitable Michael acceptor that can react with benzylamine or any primary amine to produce the intermediate A. There is a conjugated system in intermediate A that allows malononitrile to attack through Michael addition and produces the intermediate B (Scheme 2). Due to the presence of acidic hydrogen in intermediate B, it is possible to form chromonyl-malononitrile conjugated system C and to eliminate the phenylhydrazine. Intermediate C is a highly potent Michael acceptor that may allow the addition of hydrazine to the nitrile group resulting in intermediate D that after elimination of water led to intermediate E and this intermediate in the presence of a base can be converted to the **4a**–**m** after tautomerization. The compounds **5a**–**d** were formed through the same procedure without the base and after air oxidation. To confirm the mechanism, the reaction was done under inert atmosphere and the products **5a**–**d** were not formed. The reaction mechanism is shown in Scheme 2.

**Figure 4:**
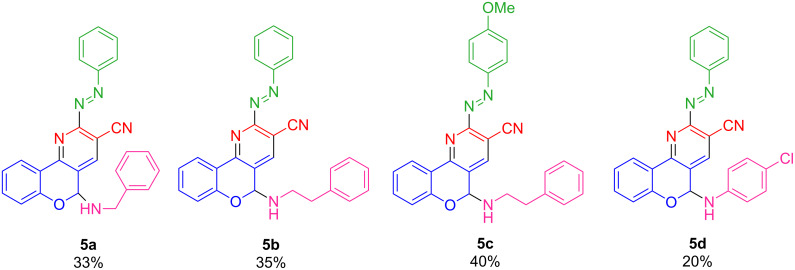
Structures of synthesized compounds **5a**–**d**.

The chemical structures of the products **5a**–**d** are shown in Figure 4.

Usually, to activate the nitrile group for cyclization reaction, the existence of Lewis acid, the addition of organolithium reagents or metal hexamethylenedisalazane is necessary. However, in this approach, the nucleophilic addition on the nitrile group and the cyclization was achieved without the addition of a catalyst [44]. This reaction possesses a highly atom economical character and requires only triethylamine (40 mol %) as a base in EtOH and provides fused heterocyclic backbones with high selectivity.

## Conclusion

In conclusion, we have successfully established an efficient route toward the synthesis of a diverse array of fused heterocyclic skeletons through a domino reaction. We showed an interesting behavior of the base for the cyclization reaction and provided access to fused heterocycles pyrazolopyridines containing chromane. The same reaction without base led to the formation of pyridochromanes **5a**–**d**. The presented procedure provides several advantageous features including domino reaction character, one-pot procedure, high selectivity, mild reaction conditions, the simplicity of operation, high atom-economy, and good to high yields.

## Experimental

### General information

Reagents and solvents were purchased from various commercial sources and were used directly without any further purification unless otherwise stated. ^1^H and ^13^C NMR spectra were recorded at 300 and 75 MHz, respectively. Chemical shifts were reported in parts per million (δ) using TMS, and coupling constants were expressed in Hertz. Melting points were recorded using an electrothermal capillary melting point apparatus and were uncorrected. HRMS spectra were recorded using high-resolution mass spectra and were recorded on Mass-ESI-POS(FT-ICR-Qe) spectrometer.

### General procedure for the synthesis of compounds **1a–g**

In a 25 mL flask containing 3 mL ethanol, 3-formylchromone derivatives (1 mmol) and hydrazine derivatives (1 mmol) were added and the mixture stirred for three hours at room temperature. The formed precipitate was filtered and washed with ethanol. Yields: 75–92%.

### General procedure for the synthesis of compounds **4a–m**

To a 25 mL flask containing 3 ml ethanol, 1 mmol (arylhydrazono)methyl-4*H*-chromen-4-one derivatives (**1a**–**g**), 1.2 mmol primary amines (**2a**–**d**), 1.2 mmol malononitrile (**3**), and triethylamine amine (40 mmol %) were added and the mixture was stirred at room temperature for 8–12 hours. The progression of the reaction was monitored by TLC (eluent ethyl acetate/*n*-hexane 3:1). After completion of the reaction, the yellow precipitate (**4a**–**m**) was filtered and washed with water and ethanol. Yields: 65–80%.

### General procedure for the synthesis of compounds **5a–d**

In a 25 mL flask containing 3 ml ethanol, 1 mmol (arylhydrazono)methyl-4*H*-chromen-4-one derivatives (**1a**,**b**), 1.2 mmol amine derivatives or 4-chloroaniline (**2a**–**c**), and 1.2 mmol malononitrile (**3**) were added and the mixture was stirred at room temperature for 3–4 hours. The progress of the reaction was monitored by TLC (eluent ethyl acetate/*n*-hexane 1:2). After completion of the reaction, the yellow precipitate (**5a**–**d**) was filtered and washed with water and ethanol. Yields: 20–40%.

## Supporting Information

File 1 Analytical and spectroscopic data.
