# Supplementary material for: Efficient synthesis of pyrazolopyridines containing a chromane backbone through domino reaction
Source: Beilstein J Org Chem. 2019 Apr 11;15:874–80. doi: 10.3762/bjoc.15.85 (PMC6466694; doi:10.3762/bjoc.15.85)
Supplement: File 1 — Analytical and spectroscopic data. [file Beilstein_J_Org_Chem-15-874-s001.pdf]

BEILSTEIN JOURNAL OF ORGANIC CHEMISTRY

Supporting Information

for

Efficient synthesis of pyrazolopyridines containing a chromane backbone through domino reaction

Razieh Navari, Saeed Balalaie, Saber Mehrparvar, Fatemeh Darvish, Frank Rominger, Fatima Hamdan and Sattar Mirzaie

Beilstein J. Org. Chem. **2019,** *15,* 874–880. doi:10.3762/bjoc.15.85

Analytical and spectroscopic data

License and Terms: This is a supporting information file under the terms of the Creative Commons Attribution License (http://creativecommons.org/licenses/by/4.0). Please note that the reuse, redistribution and reproduction in particular requires that the authors and source are credited.
The license is subject to the *Beilstein Journal of Organic Chemistry* terms and conditions: (https://www.beilstein-journals.org/bjoc)

3-((2-Phenylhydrazono)methyl)-4*H*-chromen-4-one (1a): Yellow powder (227 mg, 86% yield). m.p.: 239-241 ^oC.

IR ν_{max} (KBr, cm^{-1}): 3266, 3046, 1632.

^{1}H NMR (300 MHz, $CDCl_3$) δ (ppm): 6.74 (*t*, H, J= 7.1Hz, H-Ar), 7.05 (*d*, 2H, *J*= 7.9Hz, H-Ar), 7.20 (*t*, 2H, *J*= 7.5Hz, H-Ar), 7.50 (*t*, 1H, *J*= 7.4Hz, H-Ar), 7.67 (*d*, 1H, J= 8.4Hz, H-Ar), 7.81 (*t*, 1H, J= 7.4Hz, H-Ar), 7.96 (*s*, 1H, =CH), 8.11 (*d*, 1H, *J*= 7.8Hz, H-Ar), 8.79 (s, 1H, =CH-O), 10.48 (*s*, 1H, NH).

^{13}C {1H} NMR (75 MHz, DMSO-d_6) δ (ppm): 112.0, 118.6, 118.9, 119.6, 123.2, 125.2, 125.6, 127.9, 129.0, 134.2, 145.0, 152.2, 155.2, 174.9.

Anal. Calcd. for $C_{16}H_{12}N_2O_2$ (264/3): C 72.72, H 4.58, N 10.60, O 12.11; Found: C 72.67, H 4.10, N 10.31, O 12.54.

N6-benzyl-9-phenyl-6,9-dihydrochromeno[4,3-b]pyrazolo[4,3-e]pyridine-6,8-diamine (4a): Yellow powder, 273 mg, 65% yield. m.p.: 136-141 ^oC.

IR ν_{max} (KBr, cm^{-1}): 3330, 1632, 1602, 1551.

^{1}H NMR (300 MHz, DMSO-d_6) δ (ppm): 3.97 (*d*, 2H, *J* = 4.0 Hz, CH_2), 4.10-4.14 (*m*, 1H, NH), 5.88 (*d*, 1H, *J* = 10.8 Hz, O-C-(sp^3)-H), 6.71 (*t*, 1H, *J* = 6.9 Hz, H-Ar), 6.80 (*d*, 2H, *J* = 6.9 Hz, H-Ar), 6.94 (*d*, 1H, *J* = 6.9 Hz, H-Ar), 7.03 (*t*, 1H, *J* = 7.2 Hz, H-Ar), 7.16 (*t*, 2H, *J* = 7.7 Hz, H-Ar), 7.22 (*d*, 1H, *J* = 6.8 Hz, H-Ar), 7.27-7.37 (*m*, 5H, H-Ar), 7.88 (*s*, 1H, H-Ar), 7.96 (*d*, 1H, *J* = 7.5 Hz, H-Ar), 8.01 (*s*, 1H, NH'), 9.30 (*s*, 1H, NH).

^{13}**C {1H} NMR** (75 MHz, DMSO-d_6) δ (ppm): 48.0, 86.6, 87.9, 112.4, 117.1, 117.7, 118.7, 120.1, 121.2, 123.5, 125.0, 126.6, 127.8, 128.1, 128.8, 129.9, 132.5, 140.1, 142.0, 149.4, 149.8, 155.2, 160.1.

HR-MS (ESI-POS): calc. for $C_{26}H_{22}N_5O$ $[M+H]^+$ 420.1819, found420.1820.

Further purification of **4a** was done by recrystallization of the sample in ethanol: Orange crystal (needle), dimensions 0.120 × 0.070 × 0.050 mm^3, crystal system monoclinic, space group *P*21/*n*, *Z* = 4, *a* = 15.586(4) Å, *b* = 9.519(2) Å, *c* = 17.628(4) Å, alpha = 90 deg, beta = 111.170(6) deg, gamma = 90 deg, *V* = 2439.1(10) Å^3, rho = 1.317 g/cm^3, *T* = 200(2) K, Thetamax = 20.818 deg, radiation Mo Kalpha, lambda = 0.71073 Å, 0.5 deg omega-scans with CCD area detector, covering the asymmetric unit in reciprocal space with a mean redundancy of 3.91 and a completeness of 99.8% to a resolution of 1.10 Å, 10272 reflections measured, 2624 unique (R(int)=0.1111), 1199 observed ($I > 2\sigma(I)$), intensities were corrected for Lorentz and polarization effects, an empirical scaling and absorption correction was applied using SADABS [1] based on the Laue symmetry of the reciprocal space, mu = 0.09 mm^{-1}, T_{min} = 0.77, T_{max} = 0.96, structure refined against F^2 with a Full-matrix least-squares algorithm using the SHELXL-2014/7 (Sheldrick, 2014) software [2], 340 parameters refined, hydrogen atoms were treated using appropriate riding models, except those at the nitrogen atoms, which were refined restrained and those at the crystal water molecule, which were not considered at all. goodness of fit 1.00 for observed reflections, final residual values R1(F)=0.084, wR(F^2) = 0.183 for observed reflections, residual electron density −0.24 to 0.25 eÅ^{-3}. CCDC 1864559 contains the supplementary crystallographic data for this paper. These data can be obtained free of charge from The Cambridge Crystallographic Data Centre via

***N*6-Benzyl-2-chloro-9-phenyl-6,9-dihydrochromeno[4,3-*b*]pyrazolo[4,3-*e*]pyridine-6,8-diamine (4b):** Yellow powder, 313 mg, 69% yield. m.p.: 198-201 ^oC.

IR $\bar{\upsilon}_{max}$ (KBr, cm^{-1}): 3371, 1664, 1530.

^{1}H-NMR (300 MHz, DMSO-d_6) δ (ppm): 3.97- 4.10 (*m*, 2H, CH_2), 4.45- 4.53 (*m*, 1H, N-H), 6.20 (*d*, 1H, O-C-(sp^3)-H), 7.05 (*d*, 1H, *J*= 8.7Hz, H-Ar), 7.23-7.31 (*m*, 5H, H-Ar), 7.36 (*t*, 2H, *J*= 7.1Hz, H-Ar), 7.48- 7.54 (*m*, 1H, H-Ar), 7.60- 7.63 (*m*, 1H, H-Ar), 7.69-7.75 (*m*, 3H, H-Ar), 8.04 (*d*, 1H, J= 1.5Hz, H-Ar), 8.07 (*s*, 1H, H-Pyridyl), 8.08 (*s*, 1H, NH), 8.54 (*s*, 1H, NH).

^{13}C-NMR (75 MHz, DMSO-d_6) δ (ppm): 48.3, 86.8. 103.2, 115.6, 120.3, 122.0, 123.5, 125.7, 126.7, 127.2, 127.9, 128.1, 128.1, 128.3, 129.9, 132.9, 133.9, 134.0, 139.8, 149.1, 151.8, 153.8, 162.0.

HR-MS (ESI-POS): calc. for $C_{26}H_{21}ClN_5O$ $[M+H]^+$ 452.1290, found 452.1293.

***N*6-Phenethyl-9-phenyl-6,9-dihydrochromeno[4,3-*b*]pyrazolo[4,3-e]pyridine-6,8-diamine (4c):** Yellow powder, 277 mg, yield 64%. m.p.: 221-225 ^oC.

IR $\bar{\upsilon}_{max}$ (KBr, cm^{-1}): 3458, 3205, 1638, 1550.

^{1}H-NMR (300 MHz, DMSO-d_6) δ (ppm): 2.81 (*t*, 2H, *J*= 7.2Hz, CH_2), 3.06-3.13 (*m*, 2H, CH_2), 3.54 (*m*, 1H, NH), 5.97 (*d*, 1H, *J* = 8.8 Hz, O-C(SP^3)-H), 6.72 (*s*, 2H, NH_2), 6.99 (*d*, 1H, *J* = 7.9 Hz, H-Ar), 7.08 (*t*, 1H, *J* = 7.3 Hz, H-Ar), 7.15-7.25 (*m*, 5H, H-Ar), 7.36 (*t*, 1H, *J* = 7.1 Hz, H-Ar), 7.45 (*t*, 1H, *J* = 7.0 Hz, H-Ar), 7.58 (*t*, 2H, *J* = 7.2 Hz, H-Ar), 7.74 (*d*, 2H, *J* = 7.5 Hz, H-Ar), 8.23 (*d*, 1H, *J* = 6.7 Hz, H-Ar), 8.27 (*s*, 1H, H-Pyridyl).

^{13}C-NMR (75 MHz, DMSO-d_6) δ (ppm): 36.4, 46.5, 88.7, 101.0, 117.8, 119.1, 121.4, 123.3, 124.2, 125.1, 126.0, 127.7, 128.3, 128.6, 128.9, 129.4, 131.5, 138.7, 140.3, 141.5, 150.4, 155.1, 157.0.

HR-MS (ESI-POS): calc. for $C_{27}H_{24}N_5O$ $[M+H]^+$ 434.1975, found 434.1977.

***N*6-Cyclohexyl-9-phenyl-6,9-dihydrochromeno[4,3-*b*]pyrazolo[4,3-*e*]pyridine-6,8-diamine (4d):** Yellow powder, 251 mg, 61% yield. m.p.: 253-257 ^{o}C.

IR $\bar{\upsilon}_{max}$ (KBr, cm^{-1}): 3455, 3200, 3143, 1634, 1550.

^{1}H-NMR (300 MHz, DMSO-d_6): 1.14-1.29 (*m*, 5H, CH-Cyclohexyl), 1.51-1.55 (*m*, 1H, CH-Cyclohexyl), 1.64-1.72 (*m*, 2H, CH-Cyclohexyl), 1.84-1.90 (*m*, 1H, CH-Cyclohexyl), 1.98-2.02 (*m*, 1H, CH-Cyclohexyl), 2.90.3.00 (*m*, 1H, CH-N), 3.25 (dd, 1H, *J* = 11.4 , 3.8 Hz, NH),6.02 (*d*, 1H, *J* = 11.5 Hz, O-C-(sp^3)-H), 6.70 (*s*, 2H, NH_2), 6.96 (*d*, 1H, *J* = 8.0 Hz, H-Ar), 7.07 (*t*, 1H, *J* = 7.5 Hz, H-Ar), 7.35 (*t*, 1H, *J* = 7.2 Hz, H-Ar), 7.44 (*t*, 1H, *J* = 7.2 Hz, H-Ar), 7.58 (*t*, 2H, *J* = 7.6 Hz, H-Ar), 7.74 (*d*, 2H, *J* = 7.8 Hz, H-Ar), 8.22 (*d*, 1H, *J* = 8.7 Hz, H-Ar), 8.25 (*s*, 1H, H-Pyridyl).

^{13}C-MR (75 MHz, DMSO-d_6): 24.3, 24.5, 25.8, 32.6, 34.2, 52.2, 86.8, 101.0, 117.9, 119.7, 121.2, 123.3, 124.2, 125.1, 127.5, 128.8, 129.4, 131.4, 138.8, 141.5, 150.5, 155.1, 156.9.

HR-MS (ESI-POS) δ (ppm): calc. for $C_{25}H_{26}N_5O$ $[M+H]^+$ 412.2132, found 412.2136.

2-fluoro-N6-phenethyl-9-phenyl-6,9-dihydrochromeno[4,3-b]pyrazolo[4,3-e] pyridine-6,8-diamine (4e): Yellow powder, 330 mg, 73% yield. m.p.: 217-225 ^{o}C.

IR $\bar{\upsilon}_{max}$ (KBr, cm^{-1}): 3455, 1635, 1549, 1503.

^{1}H-NMR (300 MHz, DMSO-d_6) δ (ppm): 2.81 (*t*, 2H, *J* = 7.2 Hz, CH_2), 3.35-3.47 (*m*, 2H, CH_2),3.50-3.65 (m, 1H, NH), 5.97 (*d*, 1H, *J* = 10.2 Hz, O-C-(sp^3)-H), 6.76 (*s*, 2H, NH_2), 7.00-7.05 (*m*, 1H, H-Ar), 7.01-7.27 (*m*, 6H, H-Ar), 7.45 (*t*, 1H, *J* = 7.1 Hz, H-Ar), 7.58 (*t*, 2H, *J* = 7.4 Hz, H-Ar), 7.74 (*d*, 2H, *J* = 7.4 Hz, H-Ar), 7.88 (*dd*, 1H, *J* = 8.5, 2.3 Hz, H-Ar), 8.28 (*s*, 1H, H-Pyridyl).

^{13}C-NMR (75 MHz, DMSO-d_6) δ (ppm): 36.3, 46.5, 88.8, 101.3, 110.3, 110.5, 118.0, 118.3 ($^2J_{C\text{-}F}$ = 24 Hz), 118.8, 119.4, 119.5 ($^3J_{C\text{-}F}$ = 7.3 Hz), 124.2, 124.4, 125.9, 127.7, 128.3, 128.6, 129.2, 129.4, 138.7, 140.2, 141.6, 149.4, 151.2, 155.5, 156.7, 158.6 ($^1J_{C\text{-}F}$ = 235 Hz).

HR-MS (ESI-POS): calc. for $C_{27}H_{23}FN_5O$ $[M+H]^+$ 452.1881, found 452.1882.

N6-benzyl-2-fluoro-9-phenyl-6,9-dihydrochromeno[4,3-b]pyrazolo[4,3-e] pyridine-6,8-diamine (4f): Yellow powder, 293 mg, 67% yield. m.p.: 177-185 °C.

IR $\bar{\upsilon}_{max}$ (KBr, cm^{-1}): 3303, 3200, 3038, 1654, 1599.

^{1}H-NMR (300 MHz, DMSO-d_6) δ (ppm): 3.98 (*brs*, 2H, CH_2), 4.02-4.20 (*m*, 1H, NH), 5.89 (*d*, 1H, *J*= 5.6Hz, O-C-(sp^3)-H), 6.81-6.85 (*m*, 2H, H-Ar), 6.93 (*d*, 1H, H-Ar), 6.90-7.10 (*m*, 3H, H-Ar), 7.22 (*d*, 1H, *J* = 6.9 Hz, H-Ar), 7.27-7.40 (*m*, 5H, H-Ar) 7.90 (*s*, 1H, H-Pyridyl), 7.97 (*d*, 1H, *J* = 7.2 Hz, H-Ar), 8.00 (*s*, 1H, NH), 9.33 (*s*, 1H, NH). **^{13}C-NMR** (75 MHz, DMSO-d_6) δ (ppm): 48.0, 86.6, 88.0, 113.5, 113.6, 115.1, 115.4, 117.0, 117.8, 121.0, 121.2, 125.0, 126.6, 127.8, 128.1, 128.5, 132.5, 140.2, 142.0, 146.0, 149.8, 154.4, 155.2, 157.5, 160.0.

HR-MS (ESI-POS): calc. for $C_{26}H_{21}FN_5O$ $[M+H]^+$ 438.1725, found 438.1727.

***N*6-Benzyl-2-bromo-9-phenyl-6,9-dihydrochromeno[4,3-*b*]pyrazolo[4,3-*e*]pyridine-6,8-diamine (4g):** Yellow powder, 329 mg, 66% yield. m.p.: 215-225 °C.

IR $\bar{\upsilon}_{max}$ (KBr, cm^{-1}): 3060, 3051, 1625, 1502.

^{1}H-NMR (300 MHz, DMSO-d_6) (ppm): 4.06 (*s*, 2H, CH_2), 4.16-4.34 (*m*, 1H, NH), 5.93 (*brs*, 1H, O-C-(sp^3)-H), 6.81 (*s*, 2H, NH_2), 6.97 (*d*, 1H, *J*=8.6 Hz, H-Ar), 7.25 (*d*, 1H, *J*= 7.3Hz, H-Ar), 7.34 (*t*, 3H, *J*= 7.5Hz, H-Ar), 7.44 (*m*, 2H, H-Ar), 7.50 (*t*, 1H, *J* = 9.4 Hz, H-Ar), 7.59 (*t*, 2H, *J*= 7.4Hz, H-Ar), 7.74 (*d*, 2H, *J*= 7.7Hz, H-Ar), 8.31 (*s*, 1H, H-Ar), 8.35 (*s*, 1H, H-Pyridyl).

^{13}C-NMR (75 MHz, DMSO-d_6) (ppm): 48.0, 88.6, 101.4, 113.0, 118.1, 118.5, 120.3, 123.3, 124.2, 125.3, 126.6, 127.2, 127.7, 127.9, 128.1, 128.8, 129.4, 133.7, 138.6, 140.2, 141.7, 149.0, 154.2, 156.7.

HR-MS (ESI-POS): calc. for $C_{26}H_{21}N_5O^{79}Br$ $[M+H]^+$ 498.0924, found 498.0927, calc. for $C_{26}H_{21}N_5O^{81}Br$ $[M+H]^+$ 500.0904, found 500.0907.

***N*6-Cyclohexyl-9-(4-fluorophenyl)-6,9-dihydrochromeno[4,3-*b*]pyrazolo[4,3-*e*] pyridine-6,8-diamine (4h):** Yellow powder, 258 mg, 60% yield. m.p.: 220-226 °C.

IR $\bar{\upsilon}_{max}$ (KBr, cm^{-1}): 3309, 3187, 3046, 1601, 1508.

^{1}H-NMR (300 MHz, DMSO-d_6) (ppm): 1.05-1.28 (*m*, 5H, CH-Cyclohexyl),1.50-1.53 (*m*, 1H, CH-Cyclohexyl), 1.58-1.72 (*m*, 2H, CH-Cyclohexyl), 1.78-1.82 (*m*, 1H, CH-Cyclohexyl), 1.89-1.93 (*m*, 1H, CH-Cyclohexyl), 2.78-2.95 (*m*, 1H, CH-Cyclohexyl), 3.35-3.48 (*m*, 1H, NH), 5.99 (*d*, 1H, *J*= 11.0Hz, O-C-(sp^3)-H), 6.79-6.84 (*m*, 2H, H-Ar), 6.92 (*d*, 1H, *J*= 8.1Hz, H-Ar), 7.00 (*t*, 3H, *J*= 8.9Hz, H-Ar), 7.36 (*t*, 1H, *J*= 7.4Hz, H-Ar) 7.80 (*s*, 1H, H-Pyridyl), 7.95 (*d*, 1H, *J*= 5.9Hz, H-Ar), 7.97 (*s*, 1H, NH), 9.28 (*s*, 1H, NH).

^{13}C-NMR (75 MHz, DMSO-d_6) (ppm): 24.3, 24.5, 25.6, 32.6, 34.2, 52.5, 85.4, 87.9, 113.5, 113.6, 115.1, 115.4, 117.0, 117.9, 118.4, 121.0, 125.0, 132.4, 142.0, 146.0,149.6,154.4,155.2,157.5.

HR-MS (ESI-POS): calc. for $C_{25}H_{25}FN_5O$ [M+H]$^+$ 430.2038, found 430.2040.

***N*6-Benzyl-9-(4-fluorophenyl)-6,9-dihydrochromeno[4,3-*b*]pyrazolo[4,3-*e*]pyridine-6,8-diamine (4i):** Yellow powder, 271 mg, 62% yield. m.p.: 193-196 ^{o}C.

IR $\bar{\upsilon}_{max}$ (KBr, cm^{-1}): 3303, 3199, 2900, 1599, 1555.

^{1}H-NMR (300 MHz, DMSO-d_6) (ppm): 3.97 (*s*, 2H, CH_2), 3.98-4.15 (m, 1H, NH), 5.89 (*s*, 1H, O-C-(sp3)-H), 6.80-6.85 (*m*, 2H, H-Ar), 6.94 (*d*, 1H, J = 8.4 Hz, H-Ar), 7.00 (*t*, 2H, J = 8.4 Hz, H-Ar), 7.04 (*t*,1H, J = 7.8 Hz, H-Ar), 7.18-7.39 (*m*, 6H, H-Ar), 7.89 (*s*, 1H, H-Pyridyl), 7.96 (*d*, 1H, J= 7.8Hz, H-Ar), 7.97 (*s*, 1H, NH), 9.31 (*s*, 1H, NH).

^{13}C-NMR (75 MHz, DMSO-d_6) (ppm): 48.0, 86.6, 88.0, 113.5, 113.6 ($^3J_{C\text{-}F}$ = 7.4 Hz), 115.1, 115.4 ($^2J_{C\text{-}F}$ = 22.3 Hz), 117.0, 117.8, 121.0, 121.2, 125.0, 126.6, 127.8, 128.1, 132.5, 140.1, 142.0, 146.0, 149.7, 154.4 ($^1J_{C\text{-}F}$ = 232.5 Hz), 155.2, 157.5 ($^1J_{C\text{-}F}$ = 232.5 Hz), 160.0.

HR-MS (ESI-POS): calc. for $C_{26}H_{21}FN_5O$ [M+H]$^+$ 438.1725, found 438.1724.

***N*6-(Furan-2-ylmethyl)-9-phenyl-6,9-dihydrochromeno[4,3-*b*]pyrazolo[4,3-*e*] pyridine-6,8-diamine (4j):** Yellow powder, 258 mg, 63%yield. m.p.: 224-233 ^{o}C.

IR $\bar{\upsilon}_{max}$ (KBr, cm^{-1}): 3282, 3201, 1634, 1609, 1547.

^{1}H-NMR (300 MHz, DMSO-d_6) (ppm): 3.95-4.10 (*m*, 3H, CH_2, NH), 5.90 (*d*, 1H, J = 10.9 Hz, O-C-(sp^3)-H), 6.36 (*d*, 1H, J = 2.6 Hz, H-Ar), 6.38 (*d*, 1H, J = 8.0 Hz, H-Ar), 6.73 (*s*,

2H, NH_2), 7.00 (*d*, 1H, *J* = 8.0 Hz, H-Ar), 7.10 (*t*, 1H, *J* = 7.4 Hz, H-Ar), 7.38 (*t*, 1H, *J* = 7.3 Hz, H-Ar), 7.45 (*t*, 1H, *J* = 7.3 Hz, H-Ar), 7.58 (*t*, 3H, *J* = 7.4 Hz, H-Ar), 7.72 (*d*, 2H, *J* = 7.7 Hz, H-Ar), 8.24 (*d*, 1H, *J* = 7.2 Hz, H-Ar), 8.29 (*s*, 1H, H-Pyridyl). **^{13}C-NMR** (75 MHz, DMSO-d_6) (ppm): 41.1, 87.8, 101.0, 107.0, 110.4, 117.9, 118.9, 121.5, 123.3, 124.2, 125.1, 127.7, 129.0, 129.4, 131.5, 138.7, 141.6, 142.0, 150.4, 153.8, 155.0, 157.0.

HR-MS (ESI-POS): calc. for $C_{24}H_{20}N_5O_2$ $[M+H]^+$ 410.1612, found 410.1614.

***N*6-Benzyl-9-(4-methoxyphenyl)-6,9-dihydrochromeno[4,3-*b*]pyrazolo[4,3-*e*] pyridine-6,8-diamine (4k):** Yellow powder, 346 mg, 77% yield; m.p.: 153-155 ^{o}C.

IR $\bar{\upsilon}_{max}$ (KBr, cm^{-1}): 3329, 3060, 1599.

^{1}H-NMR (300 MHz, DMSO-d_6) (ppm): 3.64 (*brs*, 2H, CH_2), 3.97 (*s*, 3H, OMe), 3.98-4.09 (*m*, 1H, NH), 5.88 (*brs*, 1H, O-C(SP^3)-H), 6.79 (*brs*, 3H, H-Ar), 6.94 (*d*, 1H, *J* = 7.9 Hz, H-Ar), 7.05 (*t*, 1H, *J* = 7.2 Hz, H-Ar), 7.22 (*d*, 2H, *J* = 6.9 Hz, H-Ar), 7.28- 7.37 (*m*, 5H, H-Ar) 7.70 (*s*, 1H, H-Pyridyl), 7.87 (*s*, 1H, NH), 8.00 (*d*, 1H, *J* = 7.3 Hz, H-Ar), 9.26 (*s*, 1H, NH).

^{13}C-NMR (75 MHz, DMSO-d_6) (ppm): 48.0, 55.2, 86.6, 87.9, 113.8, 114.3, 117.2, 117.6, 117.7, 121.0, 121.2, 125.1, 126.6, 127.8, 128.1, 140.2, 141.7, 142.0, 143.2, 149.7, 152.8, 155.2, 160.2.

HR-MS (ESI-POS): calc. for $C_{27}H_{24}N_5O_2$ $[M+H]^+$ 450.1925, found 450.1929, calc. for $C_{27}H_{23}NaN_5O$ $[M+H]^+$ 472.1744, found 472.1749.

***N*6-Cyclohexyl-9-(4-methoxyphenyl)-6,9-dihydrochromeno[4,3-*b*]pyrazolo[4,3-*e*] pyridine-6,8-diamine (4l):** Yellow powder, 353 mg, 80% yield. m.p.: 177-178 ^{o}C.

IR $\bar{\upsilon}_{max}$ (KBr, cm^{-1}): 3229, 3202, 1596.

^{1}H-NMR (300 MHz, DMSO-d_6) (ppm): 1.08-1.23 (*m*, 5H, CH-Cyclohexyl), 1.42-1.56 (*m*, 3H, CH-Cyclohexyl), 1.77-1.89 (*m*, 1H, CH-Cyclohexyl), 2.66-2.88 (m, 1H, CH-Cyclohexyl), 3.33-3.39 (m, 1H, NH), 3.36 (s, 3H, OMe), 5.97 (*d*, 1H, J = 8.7 Hz, O-C(SP^3)-H), 6.77 (*brs*, 4H, H-Ar), 6.92 (*d*, 1H, *J* = 7.9 Hz, H-Ar), 7.01 (*t*, 1H, *J* = 7.2 Hz, H-Ar), 7.35 (*t*, 1H, *J* = 6.9 Hz, H-Ar), 7.67 (*s*, 1H, H-Pyridyl), 7.80 (*s*, 1H, NH), 8.98 (*d*, 1H, *J* = 7.1 Hz, H-Ar), 9.20 (*s*, 1H, NH).

^{13}C-NMR (75 MHz, DMSO-d_6) (ppm): 24.3, 24.4, 25.6, 32.6, 34.2, 52.5, 55.3, 85.4, 87.8, 113.8, 114.3, 117.9, 118.1, 121.0, 125.0, 132.3, 142.0, 143.2, 149.6, 152.8, 155.2, 160.0.

HR-MS (ESI-POS): calc. for $C_{26}H_{28}N_5O_2$ $[M+H]^+$ 442.2238, found 442.2238, calc. for $C_{26}H_{27}N_5NaO_2$ $[M+Na]^+$ 464.2057, found 464.2061.

4-(8-Amino-6-(benzylamino)chromeno[4,3-b]pyrazolo[4,3-e]pyridine-9(6H)-yl) benzoic acid (4m): Yellow powder, 185 mg, 40% yield. m.p.: 172-174 °C.

IR $\bar{\upsilon}_{max}$ (KBr, cm^{-1}): 3312, 3029, 1596.

^{1}H-NMR (300 MHz, DMSO-d_6) (ppm): 3.90-4.02 (*m*, 2H, CH_2), 4.02-4.08 (*m*, 1H, NH), 5.90 (*brs*, 1H, O-C(SP^3)-H), 6.81 (*d*, 1H, *J* = 8.4 Hz, H-Ar), 6.93 (*d*, 1H, *J* = 8.0 Hz, H-Ar), 7.02 (*t*, 1H, *J* = 7.3 Hz, H-Ar), 7.12-7.34 (*m*, 6H, H-Ar), 7.76 (*d*, 2H, *J* = 8.4 Hz, H-Ar), 7.91 (*s*, 1H, NH), 7.92 (*d*, 1H, *J* = 7.7 Hz, H-Ar), 8.62 (*s*, 1H, NH), 9.45 (*s*, 1H, COOH).

^{13}C-NMR (75 MHz, DMSO-d_6) (ppm): 23.6, 48.0, 86.6, 88.0, 111.1, 117.8, 118.0, 120.9, 121.2, 121.6, 125.0, 126.6, 127.8, 128.1, 128.2, 128.4, 130.9, 123.5, 140.1, 153.0, 155.3, 159.8, 167.8.

HR-MS (ESI-POS): calc. for $C_{27}H_{22}N_5O_3$ [M+H]$^+$ 464.1717, found 464.1721, calc. for $C_{27}H_{21}N_5NaO_3$ [M+Na]$^+$ 486.1537, found 486.1541.

(*E*)-5-(Benzylamino)-2-(phenyldiazenyl)-5*H*-chromeno[4,3-*b*]pyridine-3-carbonitrile (5a): Yellow powder, 138 mg, 33% yield. m.p: 136-138°C.

IR $\bar{\upsilon}_{max}$ (KBr, cm^{-1}): 3331, 3208, 2228.

^{1}H-NMR (300 MHz, DMSO-d_6) (ppm): 3.98-4.06 (*m*, 2H, CH_2), 4.37-4.48 (*m*, 1H, NH), 6.14 (*d*, 2H, J = 10.5 Hz, O-C(SP^3)-H), 7.03 (*d*, 1H, *J* = 8.4 Hz, H-Ar), 7.14 (*t*, 1H, *J* = 7.4 Hz, H-Ar), 7.14 (*t*, 1H, *J* = 7.4 Hz, H-Ar), 7.24 (*d*, 1H, J = 7.2 Hz, H-Ar), 7.32 (*t*, 2H, *J* = 7.1 Hz, H-Ar), 7.39 (*d*, 2H, *J* = 7.1 Hz, H-Ar), 7.48 (*t*, 1H, J = 7.7 Hz, H-Ar), 7.68-7.71 (*m*, 3H, H-Ar), 8.04 (*d*, 2H, J = 6.8 Hz, H-Ar), 8.17 (*d*, 1H, J = 7.3 Hz, H-Ar) 8.47 (*s*, 1H, H-Pyridyl).

^{13}C-NMR (75 MHz, DMSO-d_6) (ppm): 48.2, 86.4, 102.4, 115.8, 118.1, 120.5, 121.9, 123.5, 125.4, 126.7, 127.8, 127.9, 128.2, 129.9, 133.5, 133.8, 139.9, 141.4, 150.5, 151.8, 155.2, 162.1.

(*E*)-5-(Phenethylamino)-2-(phenyldiazenyl)-5*H*-chromeno[4,3-*b*]pyridine-3-carbonitrile (5b): Yellow powder, 152 mg, 35% yield. m.p.: 120-122 ^{o}C.

IR $\bar{\upsilon}_{max}$ (KBr, cm^{-1}): 3373, 2220, 1593.

^{1}H-NMR (300 MHz, DMSO-d_6) (ppm): 2.76 (*t*, 2H, *J* = 7.5 Hz, CH_2), 3.02-3.08 (*m*, 2H, CH_2), 3.9-4.01 (m, 1H, NH), 6.19 (*d*, 1H, *J* = 10.1 Hz, O-C(SP^3)-H), 7.06-7.30 (*m*, 7H, H-Ar), 7.48 (*t*, 1H, *J* = 7.4 Hz, H-Ar), 7.68-7.71 (*m*, 3H, H-Ar), 8.04 (*d*, 2H, *J* = 7.0 Hz, H-Ar), 8.17 (*d*, 1H, *J* = 7.4 Hz, H-Ar), 8.30 (*s*, 1H, H-Pyridyl).

^{13}C-NMR (75 MHz, DMSO-d_6) (ppm): 36.2, 46.3, 86.7, 102.3, 115.8, 118.1, 120.3, 121.8, 1234, 125.3, 125.9, 128.1, 128.2, 128.6, 129.8, 133.5, 133.8, 140.0, 141.3, 150.5, 151.8, 155.3, 162.0.

HR-MS (ESI-POS): calc. for $C_{27}H_{22}N_5O$ [M+H]$^+$ 432.1819, found 432.1825.

(*E*)-2-((4-Methoxyphenyl)diazenyl)-5-(phenethylamino)-5*H*-chromeno[4,3-*b*]pyridine-3-carbonitrile (5c): Yellow powder, 185 mg, 40% yield. m.p.: 155-157 °C.

IR $\bar{\upsilon}_{max}$ (KBr, cm^{-1}): 3328, 3260, 2227.

^{1}H-NMR (300 MHz, DMSO-d_6) (ppm): 2.71-2.77 (*m*, 2H, CH_2), 2.96-3.21 (*m*, 2H, CH_2), 3.70-3.93 (*m*, 1H, NH), 3.92 (*s*, 3H, OMe), 6.18 (*d*, 1H, *J* = 9.1 Hz, O-C(SP^3)-H), 7.07 (*d*, 1H, *J* = 8.0 Hz, H-Ar), 7.15-7.29 (*m*, 8H, H-Ar), 7.38-7.47 (*m*, 1H, H-Ar), 8.04 (*d*, 2H, *J* = 7.4 Hz, H-Ar), 8.18 (*d*, 1H, *J* = 7.0 Hz, H-Ar), 8.26 (*s*, 1H, H-Pyridyl). **^{13}C-NMR** (75 MHz, DMSO-d_6) (ppm): 36.2, 46.3, 55.9, 86.7, 101.9, 115.1, 116.0, 118.2, 120.5, 121.8, 125.9, 126.0, 127.6, 128.2, 128.6, 140.1, 146.2, 150.4, 155.3, 162.4, 164.0.

(*E*)-5-((4-chlorophenyl)amino)-2-(phenyldiazenyl)-5*H*-chromeno[4,3-*b*]pyridine-3-carbonitrile (5d): Yellow powder, 88 mg, 20% yield. m.p. 175-177 °C.

IR $\bar{\upsilon}_{max}$ (KBr, cm^{-1}): 3373, 2220, 1593.

^{1}H-NMR (300 MHz, DMSO-d_6) (ppm): 3.34 (s, 1H, NH), 6.59 (*d*, 1H, J = 6.0 Hz, O-C(SP^3)-H), 6.69-6.77 (*m*, 7H, H-Ar), 6.80-6.96 (*m*, 4H, H-Ar), 7.03-7.22 (*m*, 5H, H-Ar), 7.25-7.36 (*m*, 1H, H-Ar), 8.02 (*brs*, 2H, H-Ar), 9.41 (*s*, 1H, H-Pyridyl).

^{13}C-NMR (75 MHz, DMSO-d_6) (ppm): 80.2, 102.4, 115.4, 115.7, 118.5, 120.6, 122.2, 122.4, 123.6, 125.4, 126.5, 128.8, 129.9, 133.6, 133.9, 141.7, 143.9, 150.1, 151.8, 154.4, 162.6.

HR-MS (ESI-POS): calc. for $C_{25}H_{17}ClN_5O$ [M+H]$^+$ 438.1111, found 438.111.

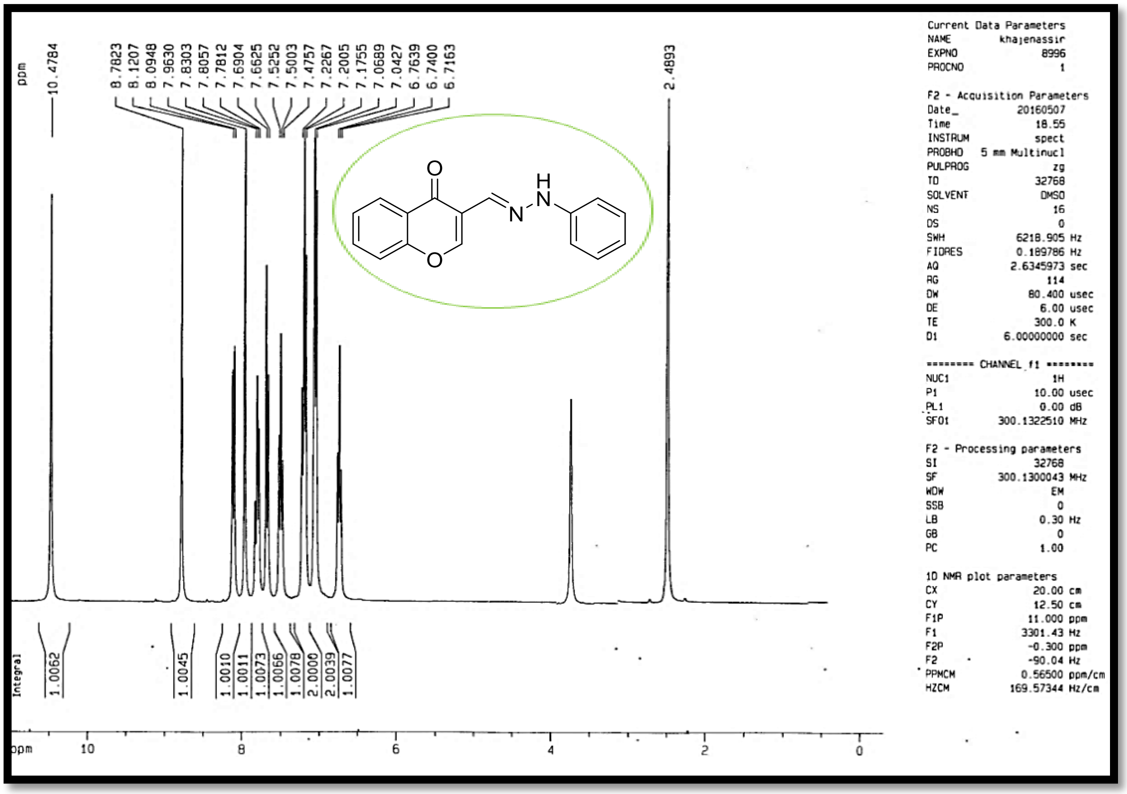

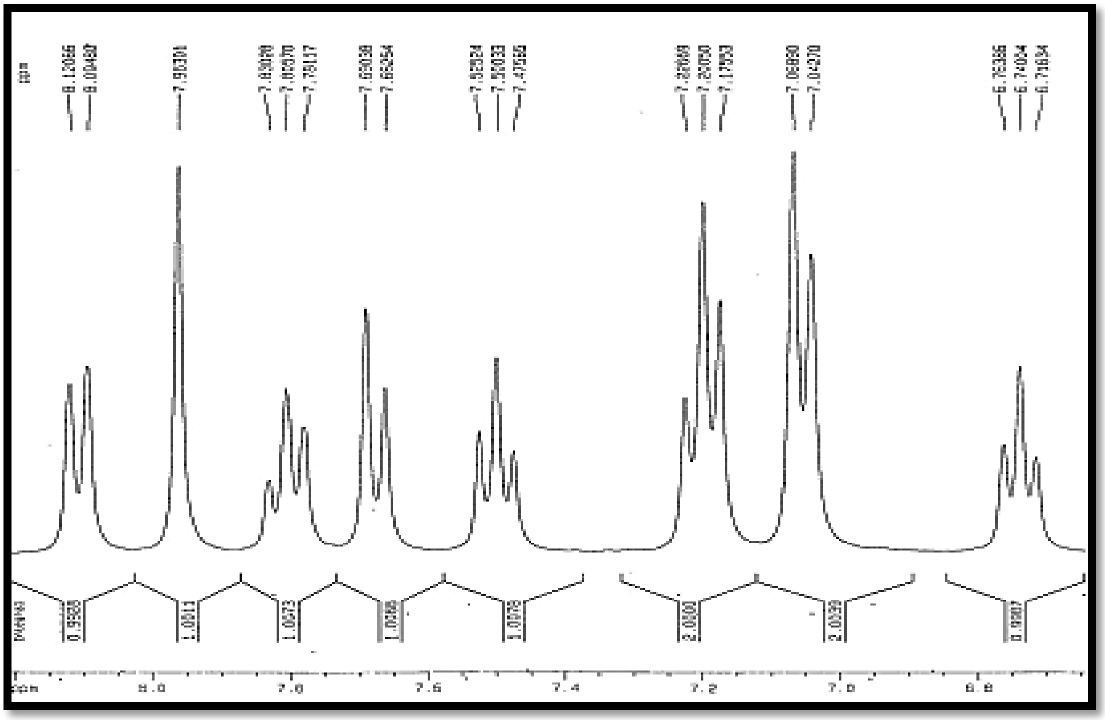

^{1}H-NMR （300 MHz, DMSO- d_6） (**1a**)

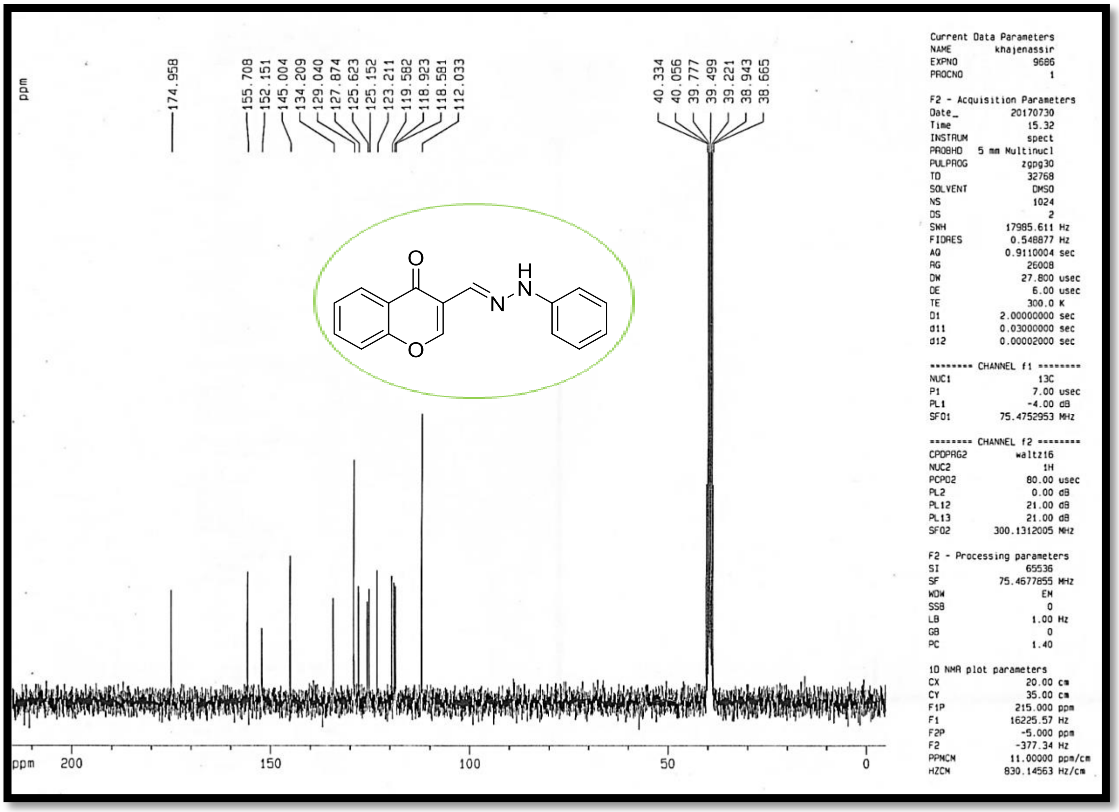

^{13}C-NMR （75 MHz, DMSO- d_6） (**1a**)

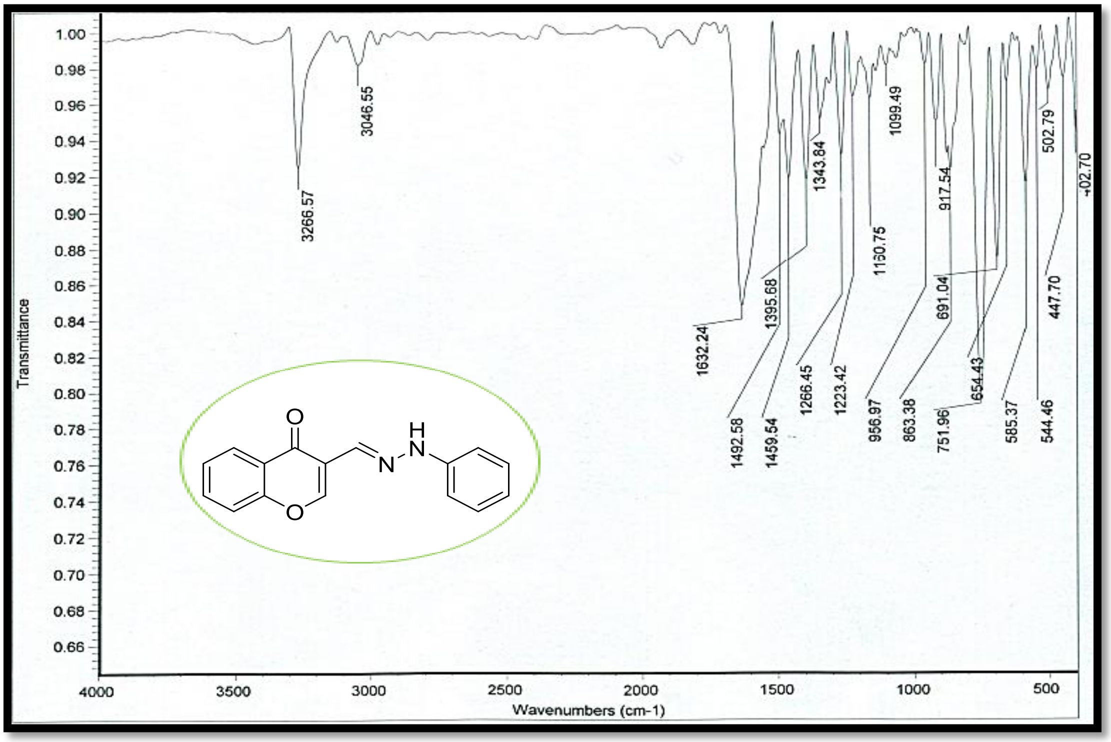

IR (KBr) (**1a**)

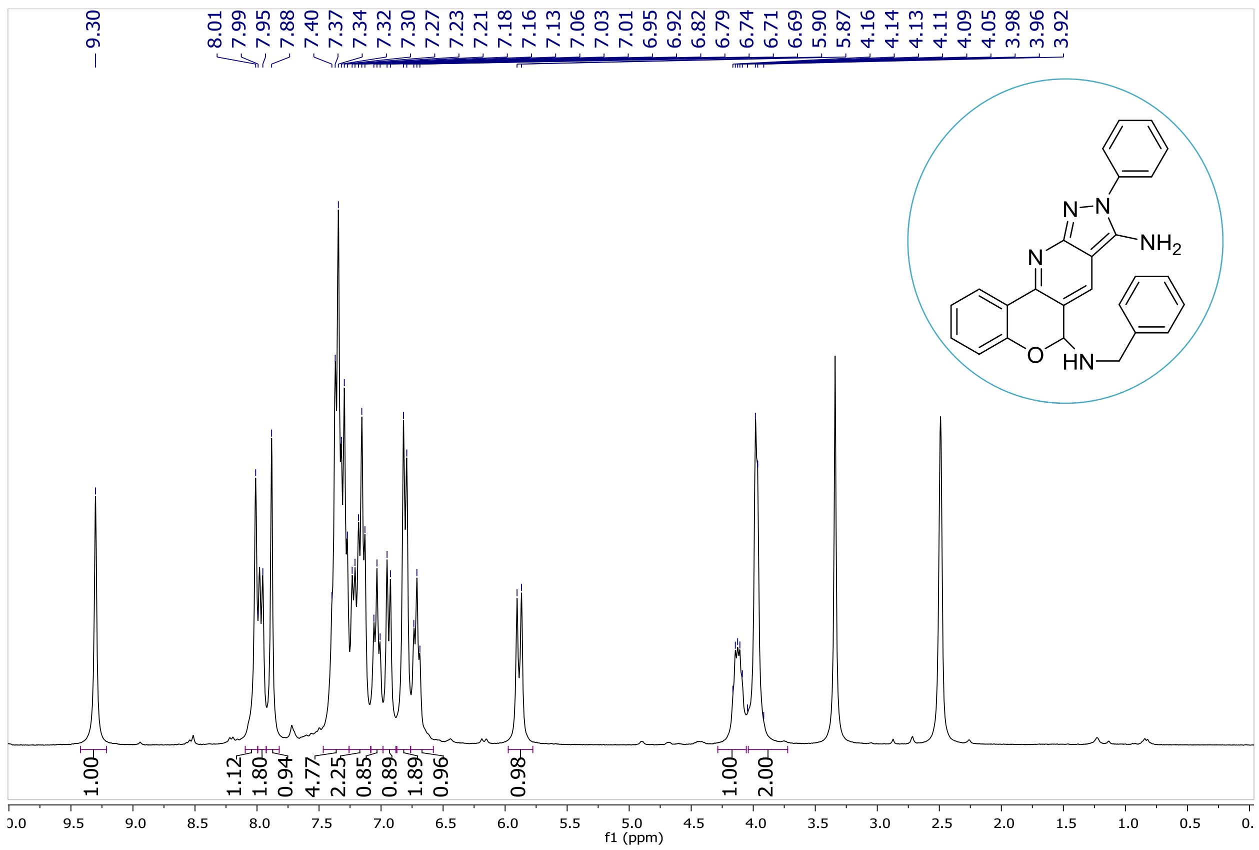

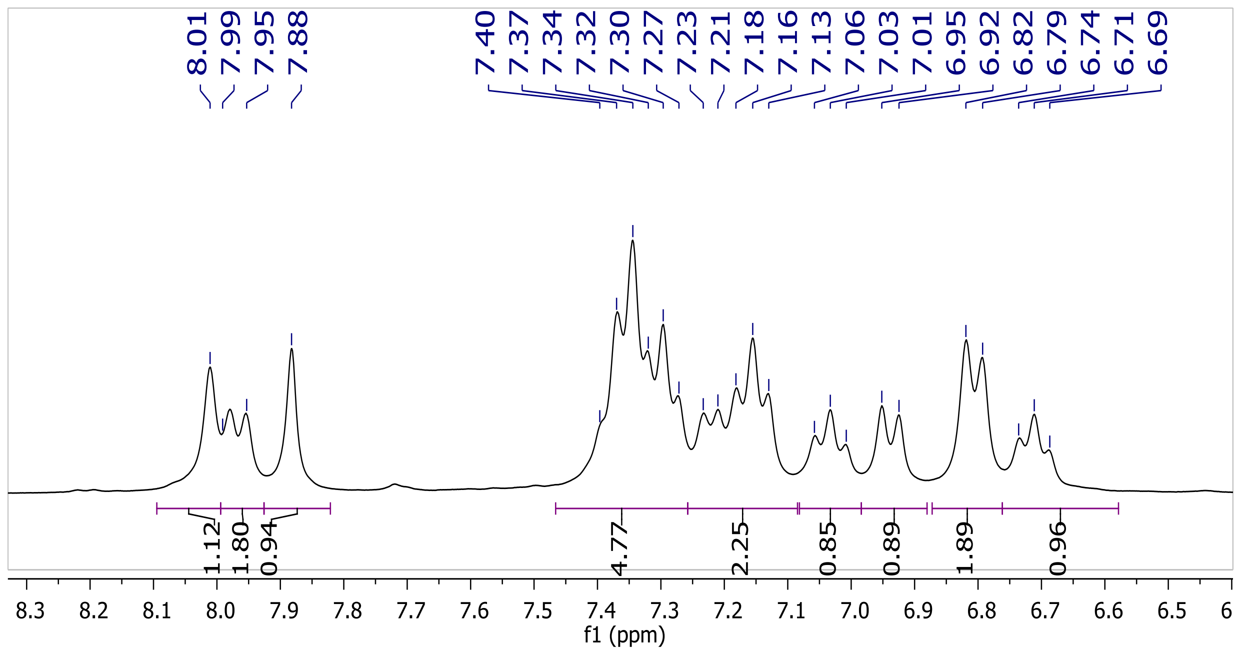

^{1}H-NMR（300 MHz, DMSO- d_6） (**4a**)

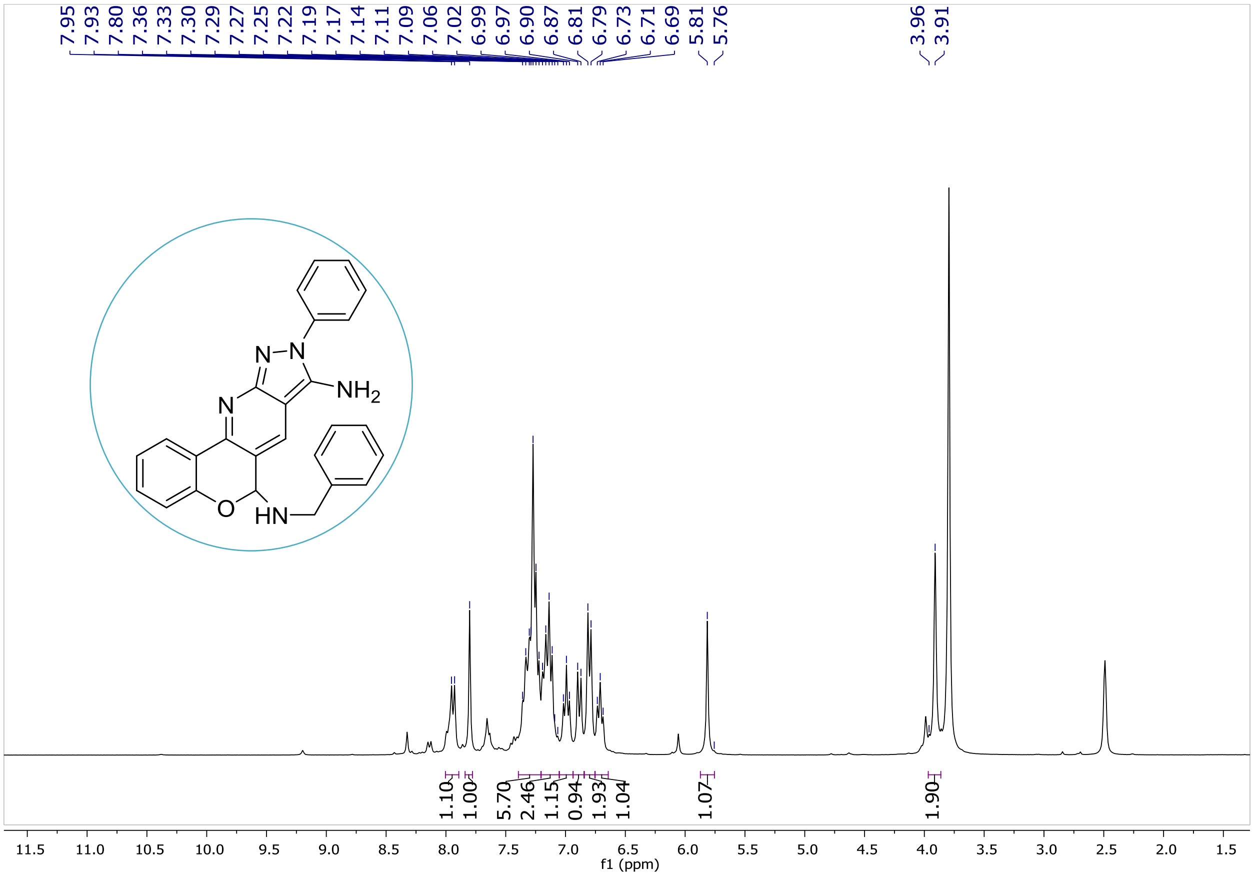

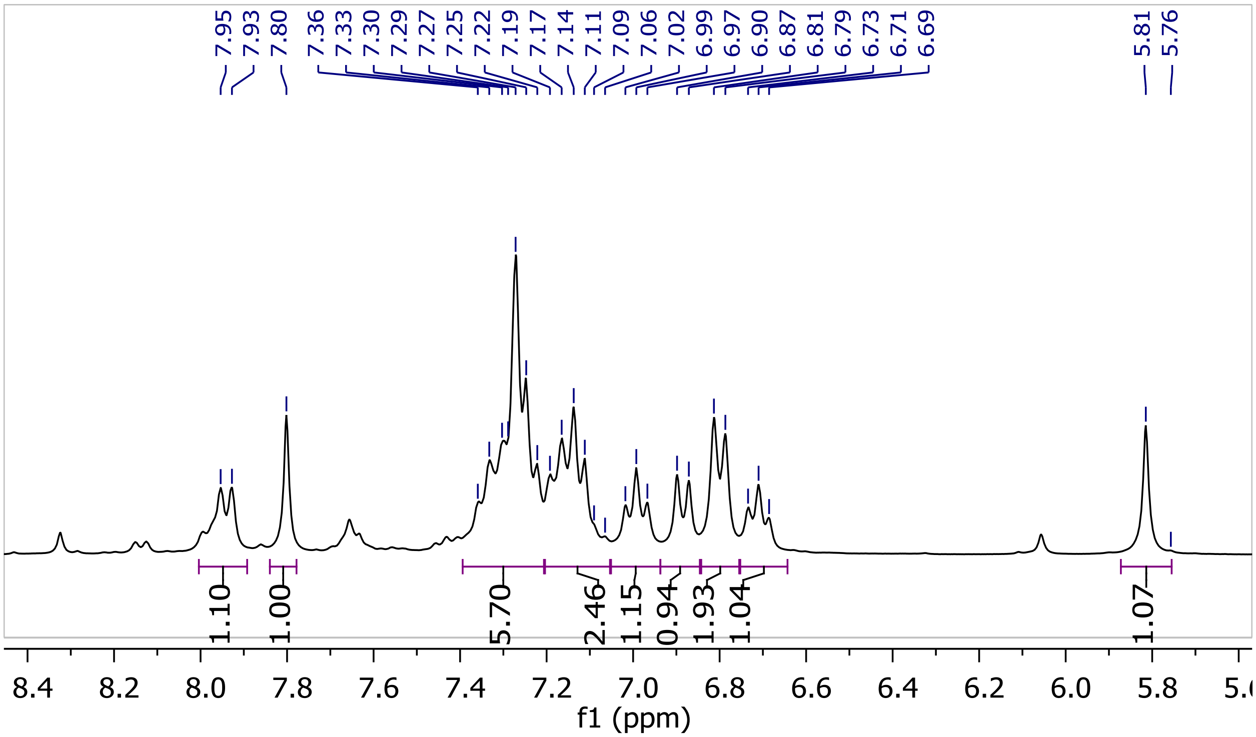

^{1}H-NMR, D_2O （300 MHz, DMSO- d_6） (**4a**)

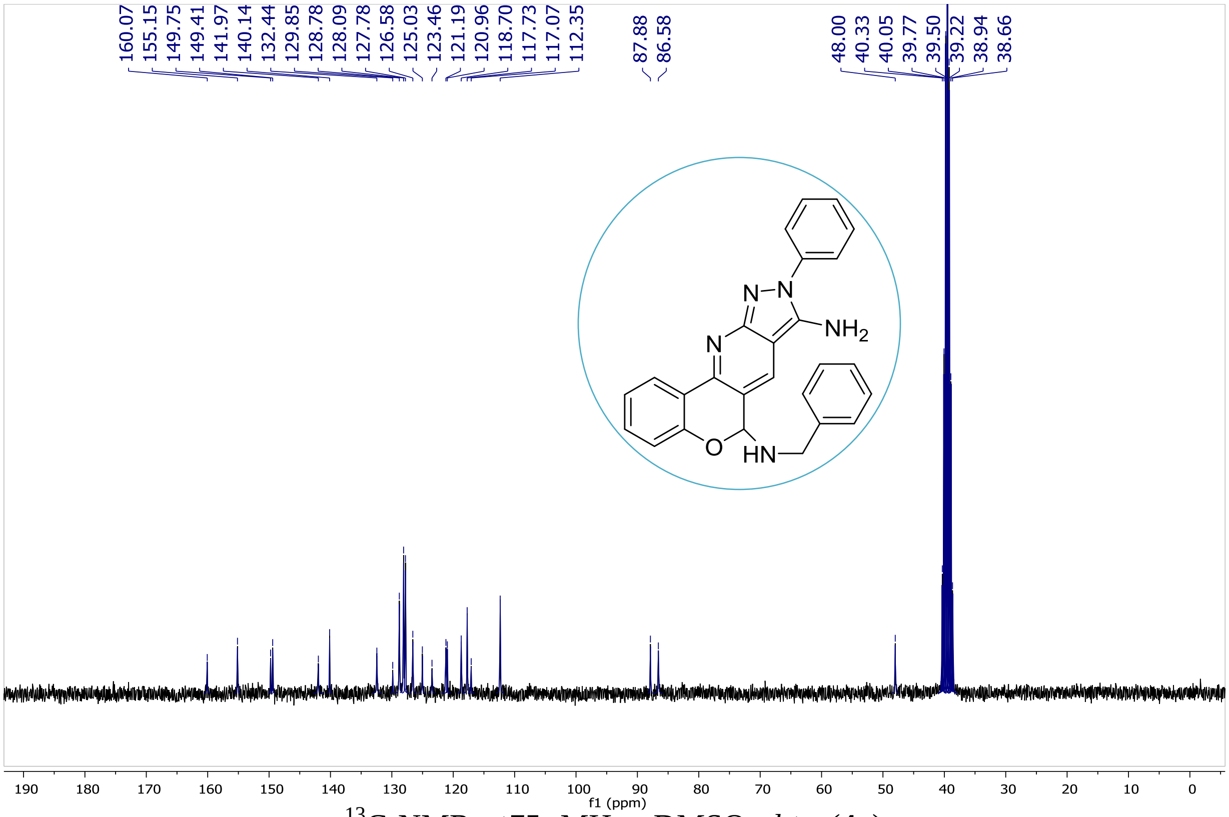

^{13}C-NMR （75 MHz, DMSO- d_6） (**4a**)

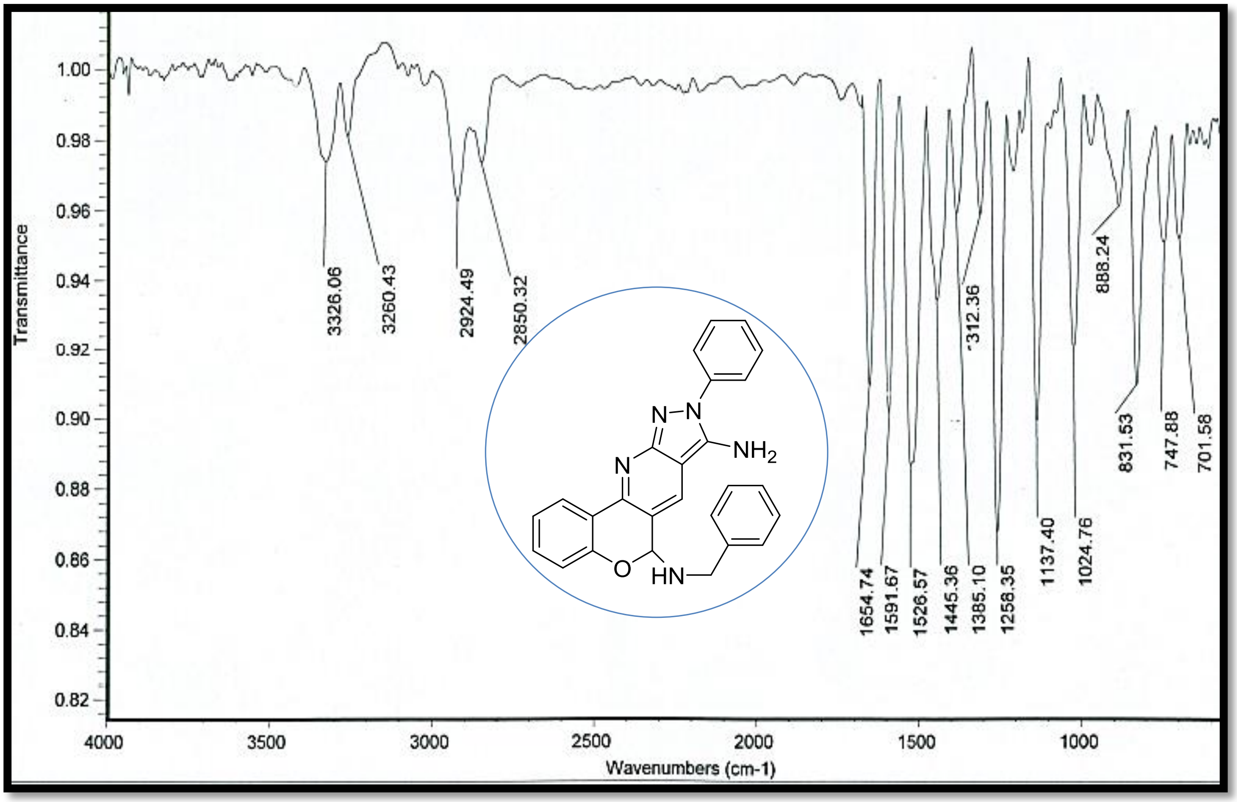

IR (KBr) (**4a**)

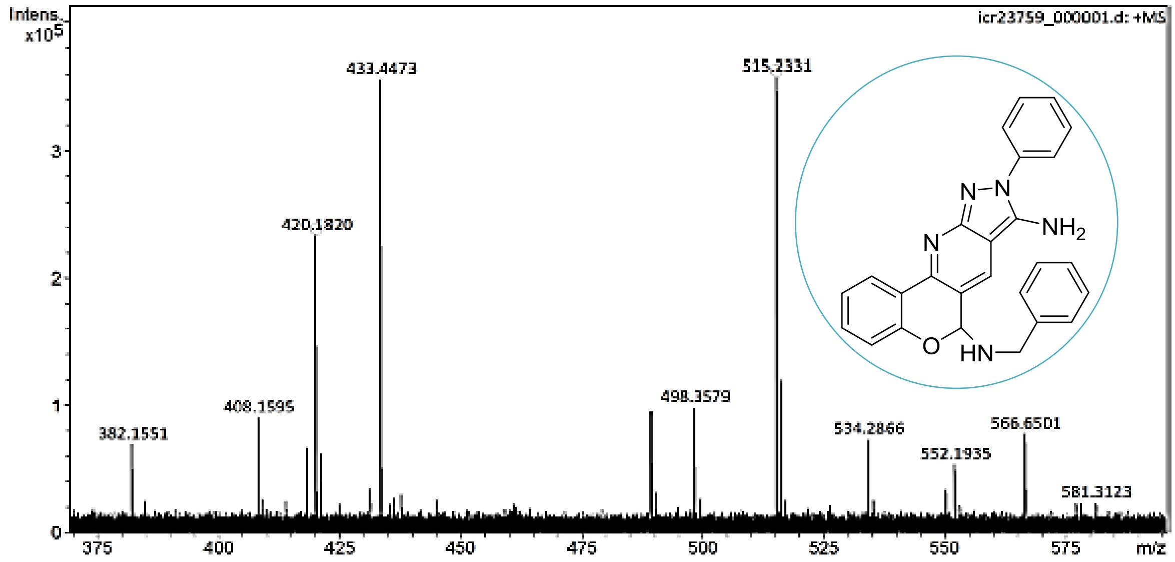

Meas. m/z	Ion Formula	m/z	err [ppm]	mSigma	rdb	e⁻ Conf	N-Rule
420.1820	C24H20N8	420.1805	-3.6	22.3	19.0	odd	ok
	C26H22N5O	420.1819	-0.4	23.6	18.5	even	ok
	C25H26NO5	420.1805	-3.6	27.2	13.5	even	ok
	C28H24N2O2	420.1832	2.8	31.1	18.0	odd	ok
515.2331	C32H29N5O2	515.2316	-2.9	11.0	21.0	odd	ok
	C34H31N2O3	515.2329	-0.3	18.0	20.5	even	ok

HR-Mass (ESI) (**4a**)

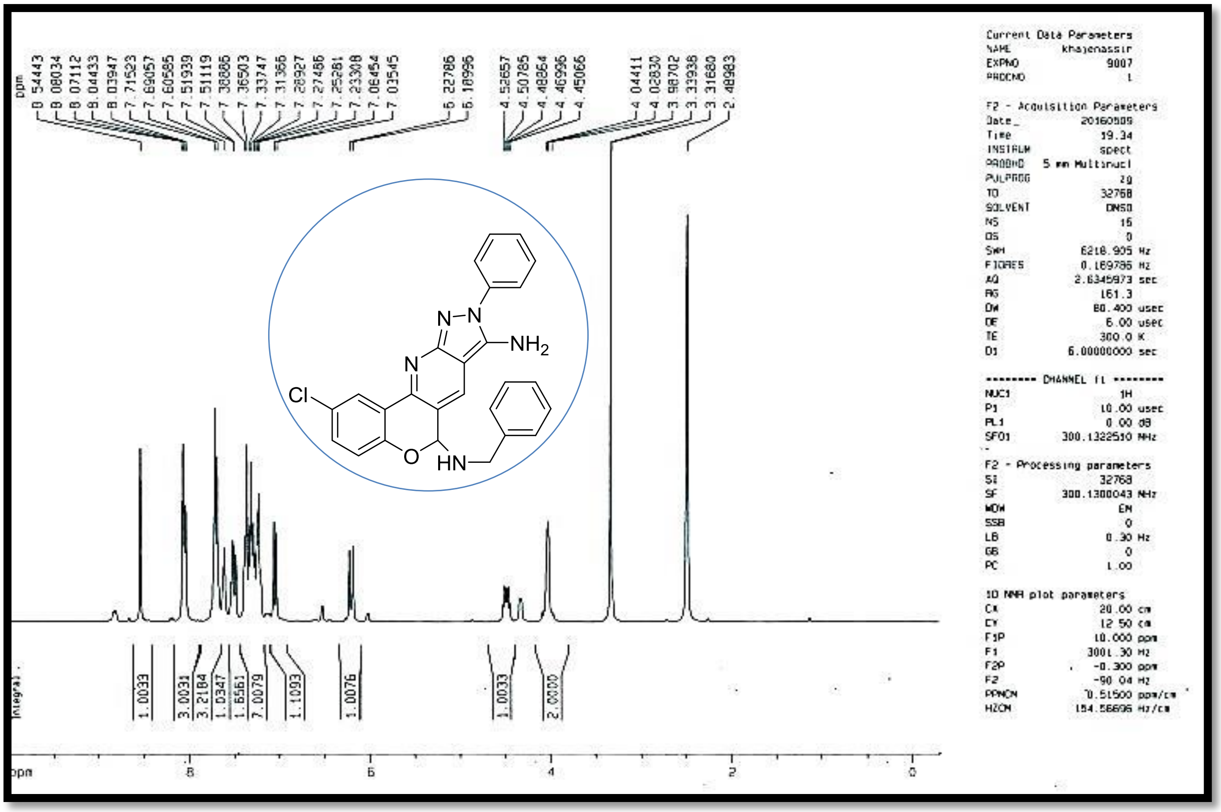

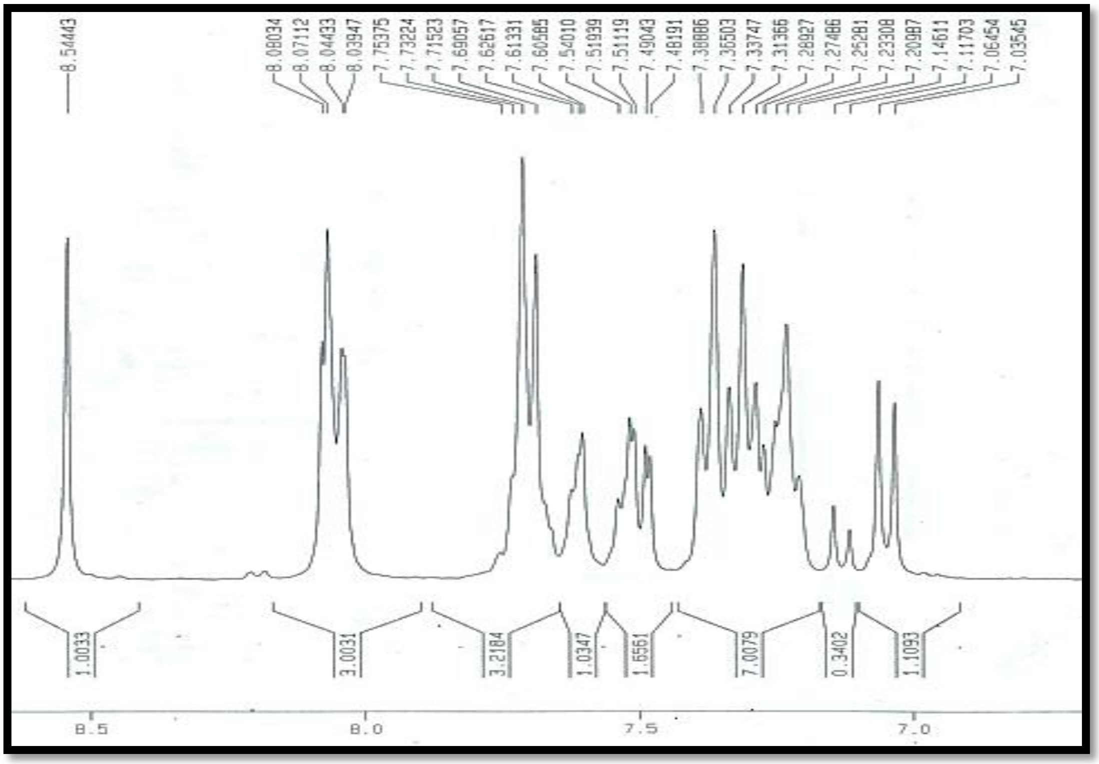

^{1}H-NMR （300 MHz, DMSO- d_6） (**4b**)

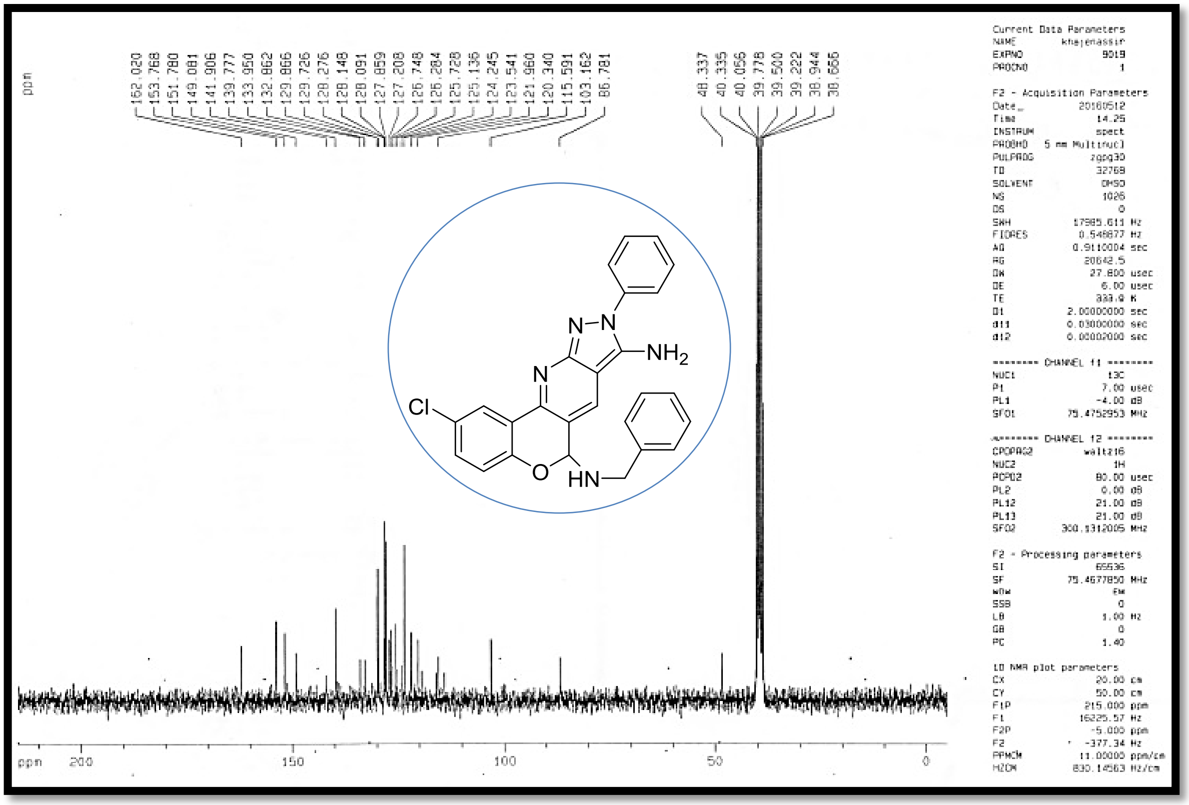

^{13}C-NMR（75 MHz, DMSO- d_6） (**4b**)

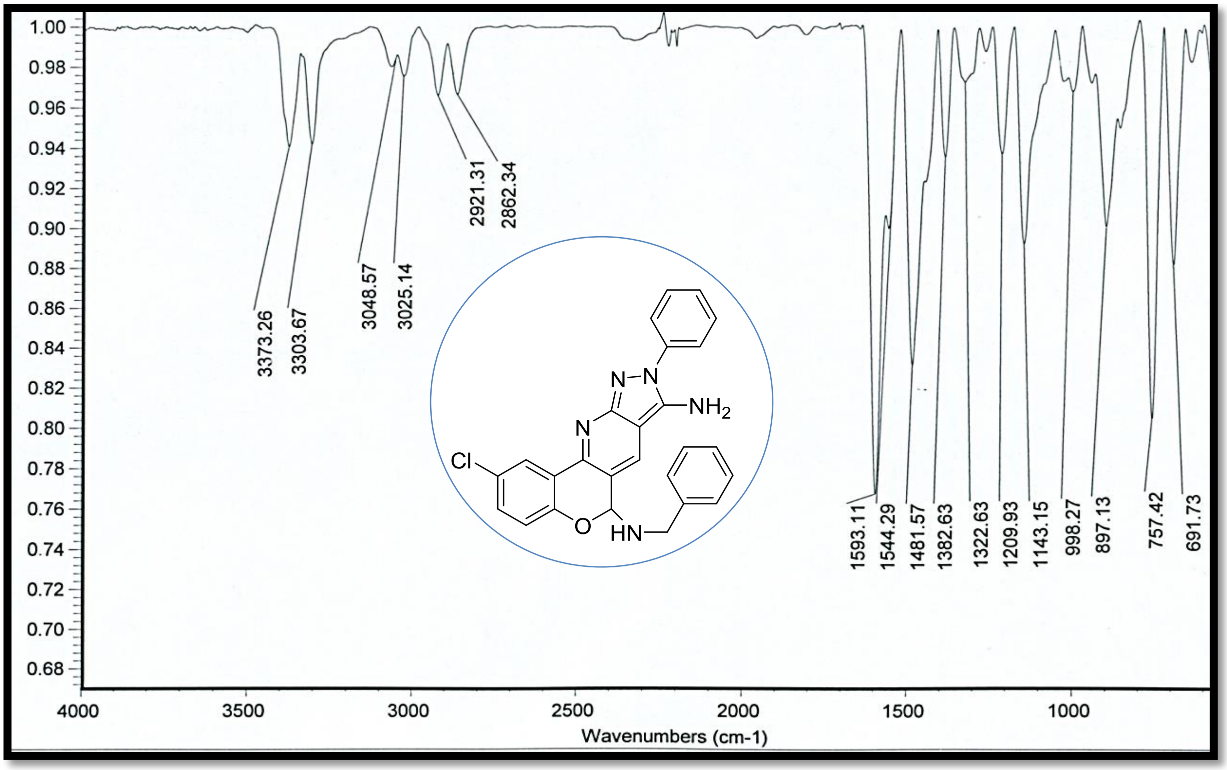

IR (KBr) (**4b**)

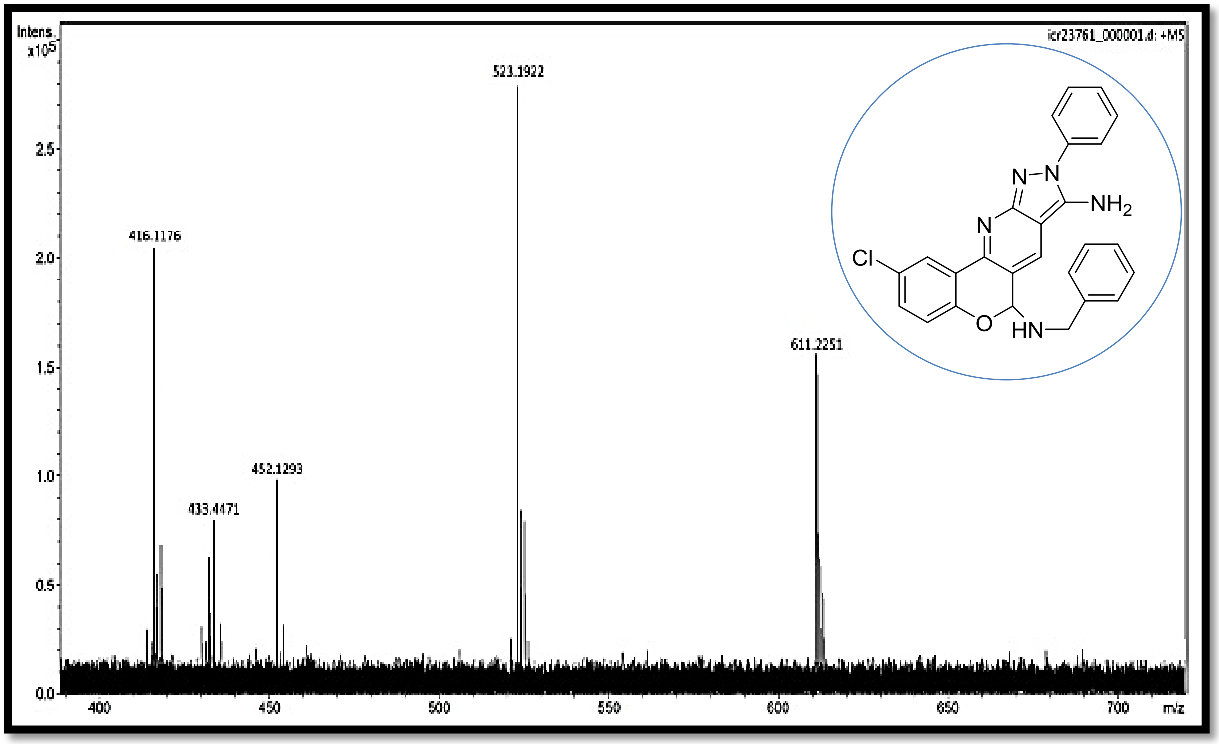

HR-Mass (ESI) (**4b**)

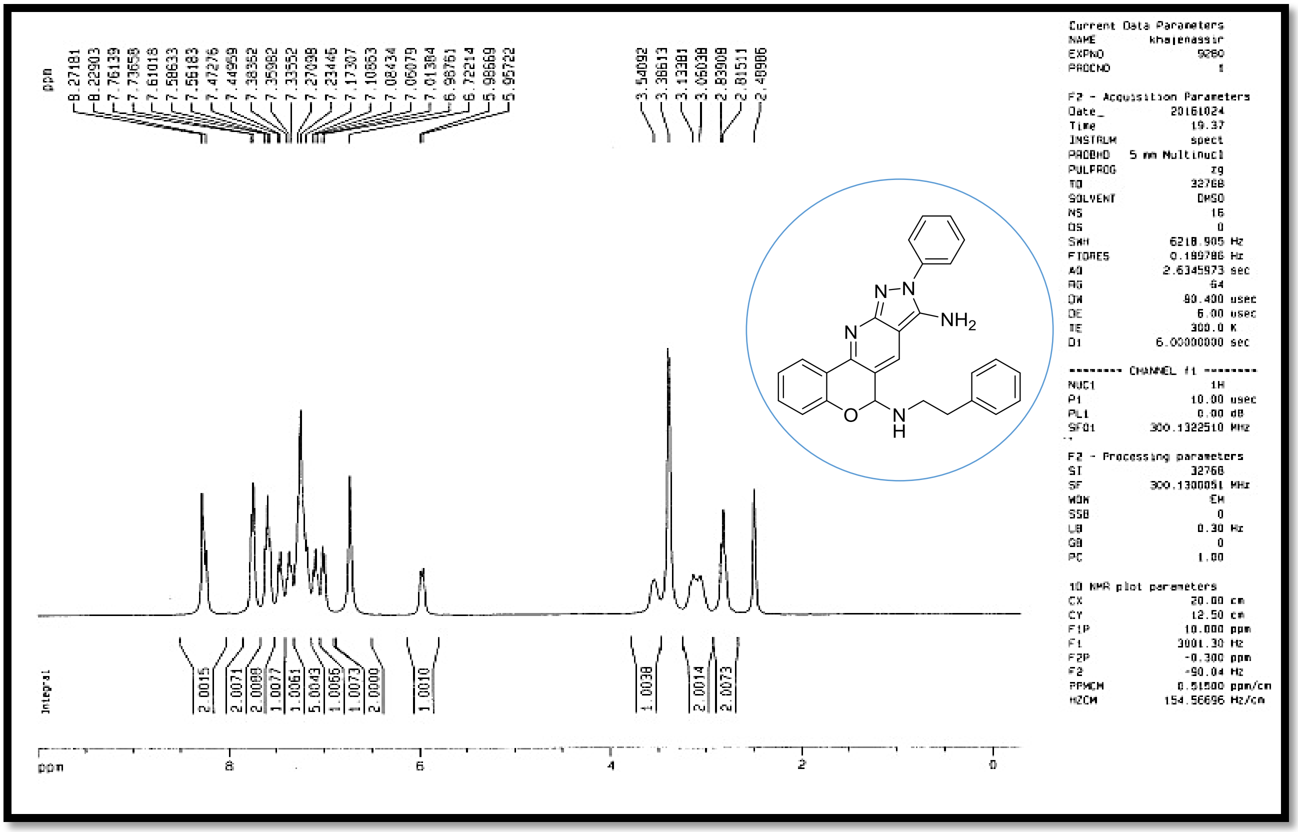

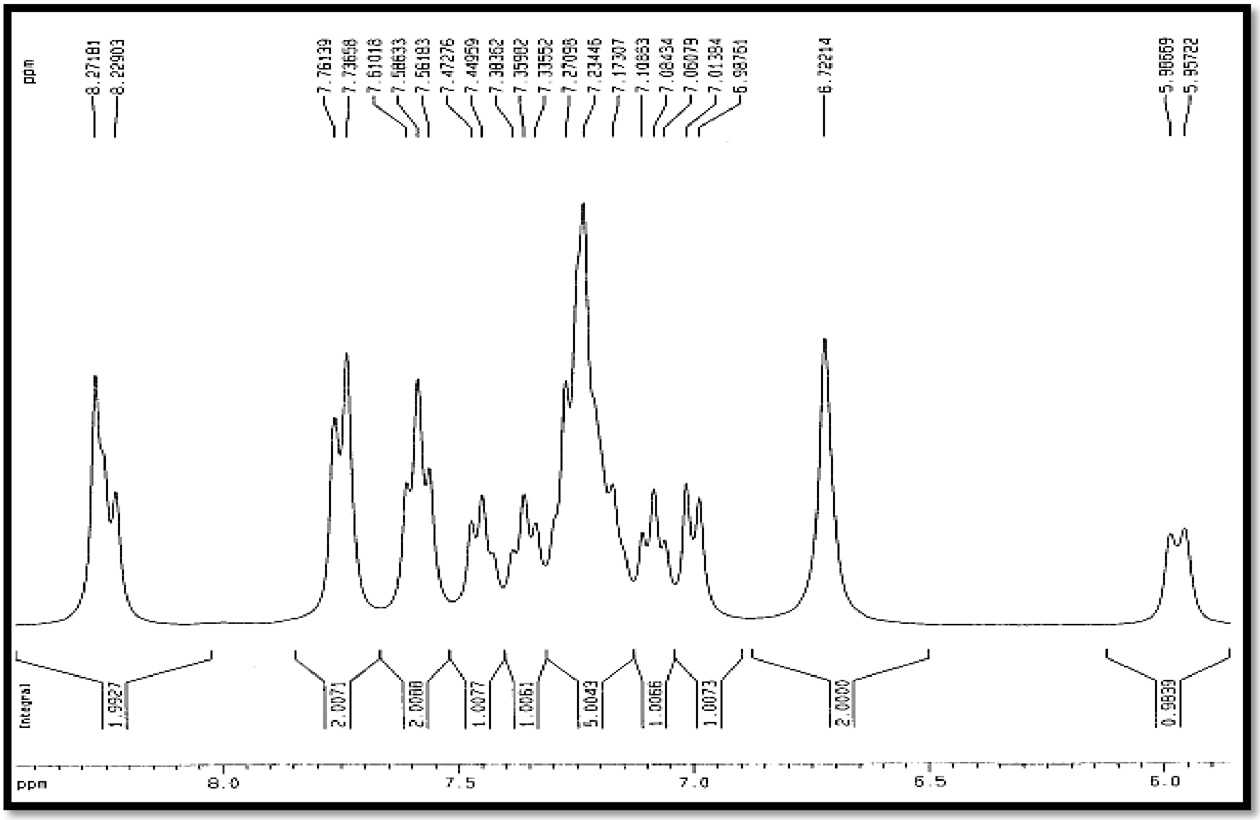

^{1}H-NMR （300 MHz, DMSO- d_6） (**4c**)

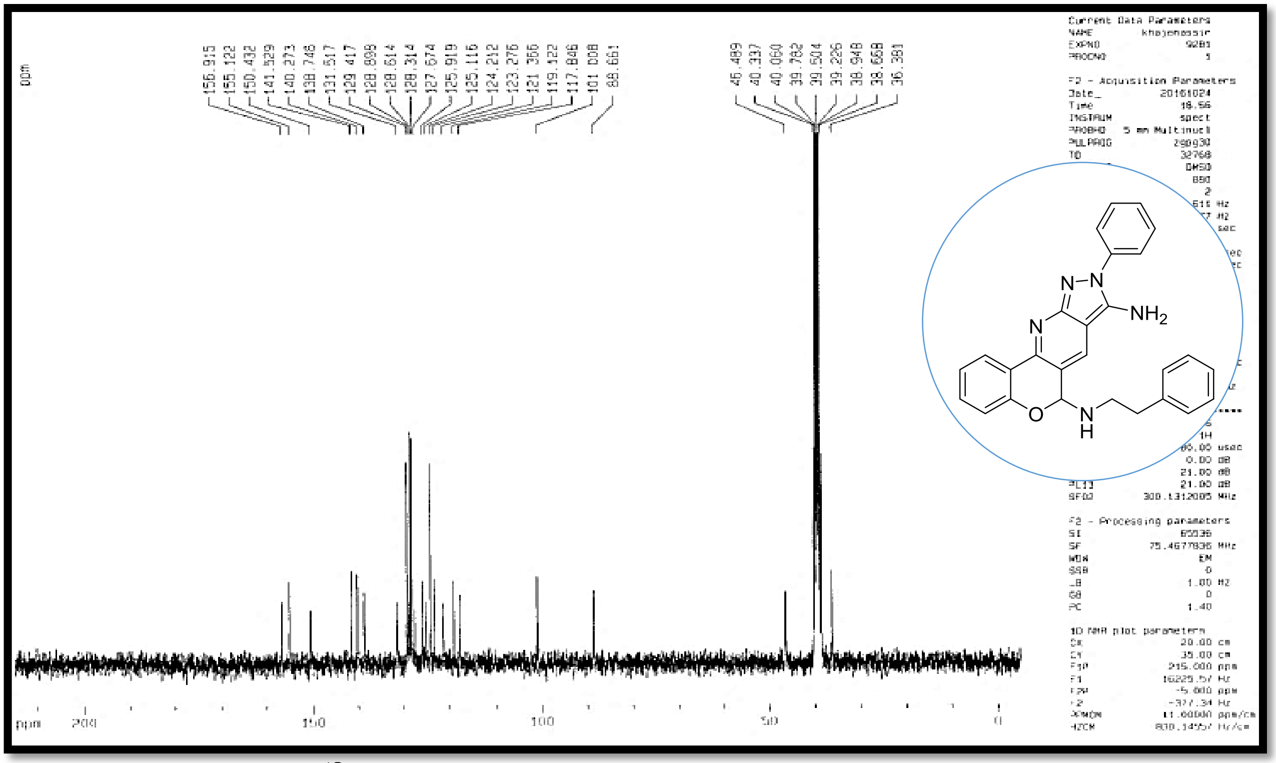

^{13}C-NMR (75 MHz, DMSO- d_6) (**4c**)

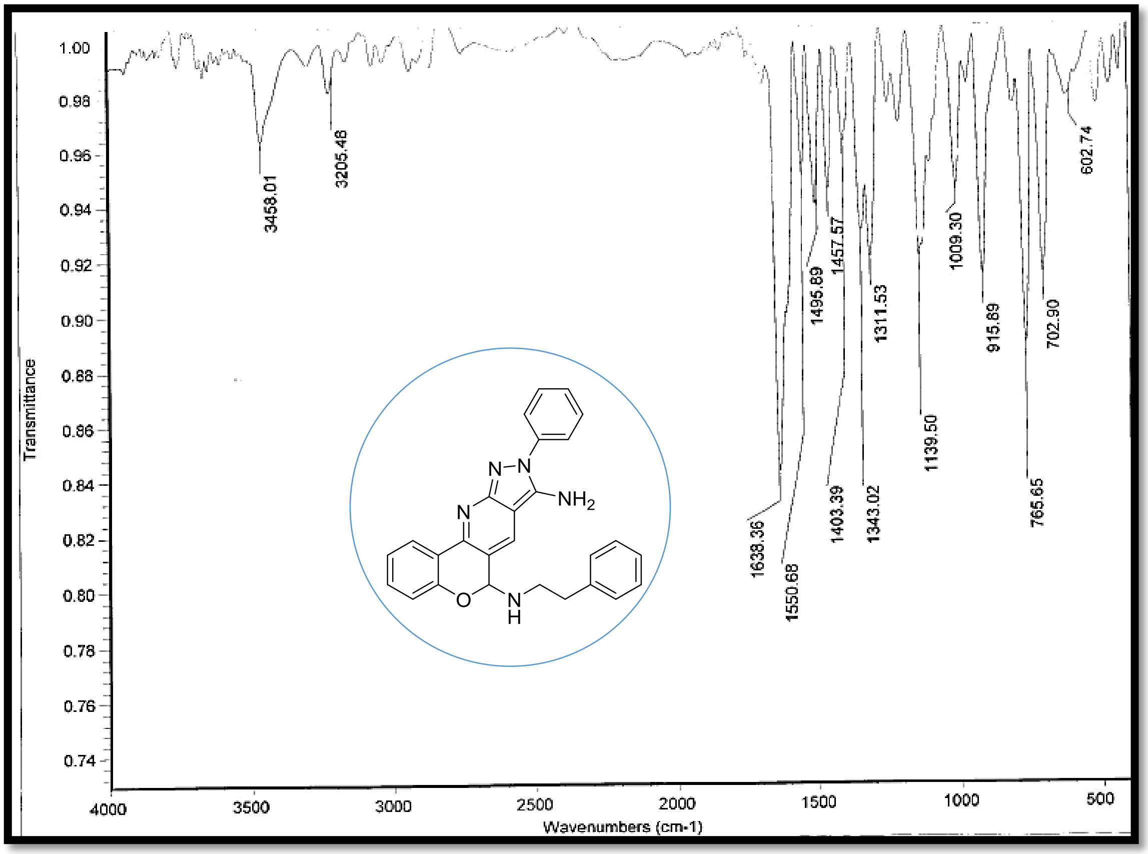

IR (KBr) (**4c**)

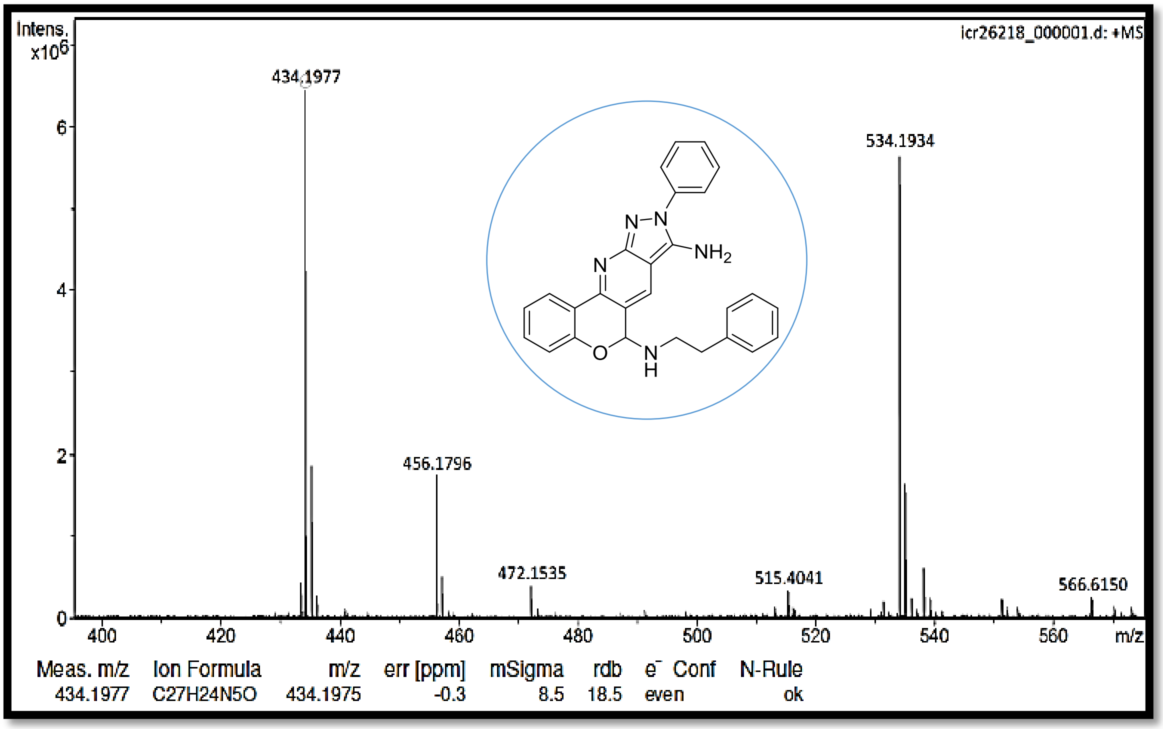

HR-Mass (ESI) (**4c**)

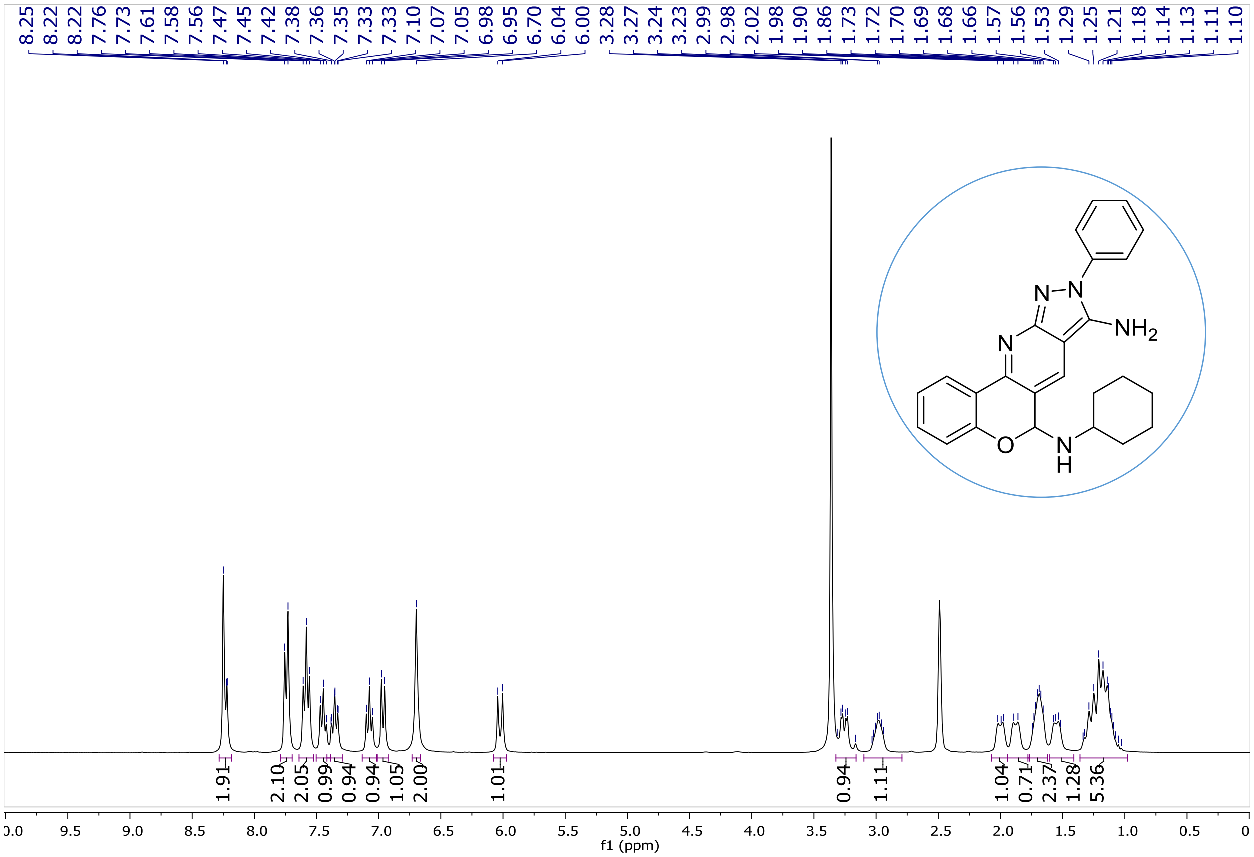

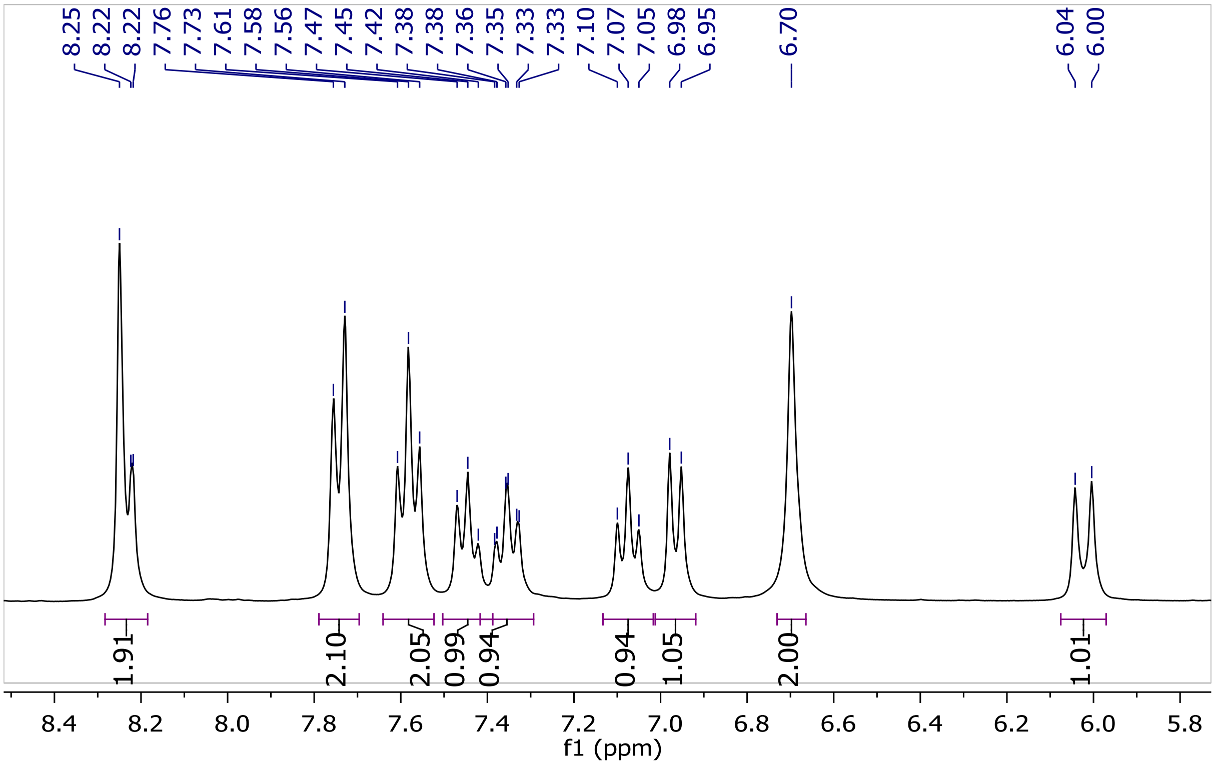

^{1}H-NMR （300 MHz, DMSO- d_6） (**4d**)

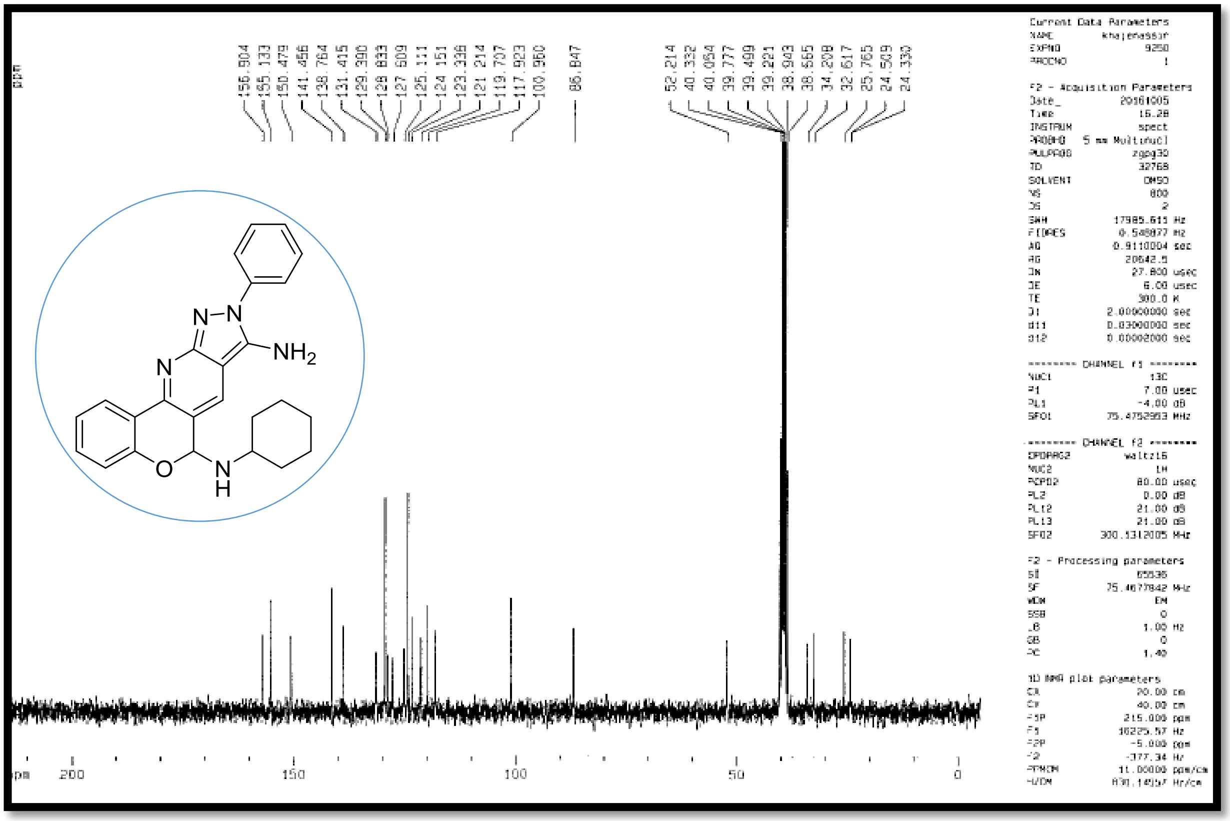

^{13}C-NMR（75 MHz, DMSO- d_6） (**4d**)

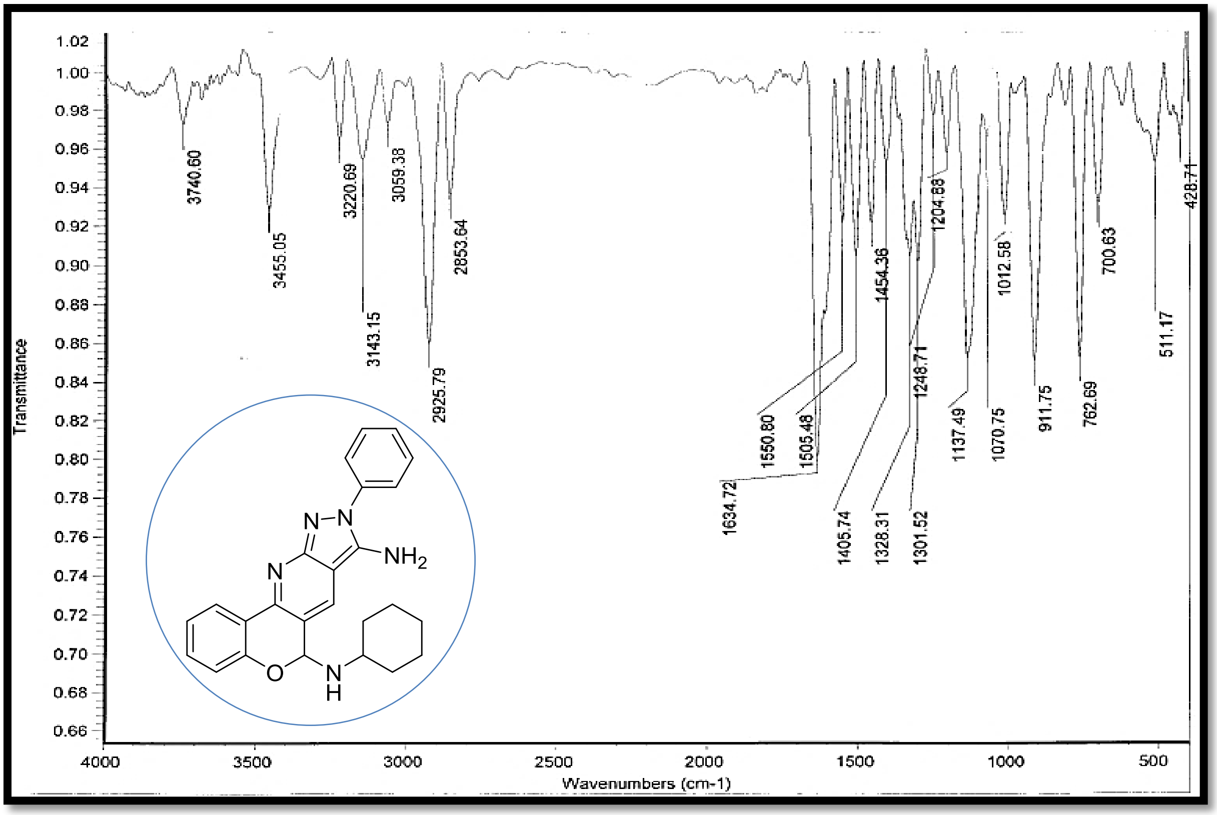

IR (KBr) (**4d**)

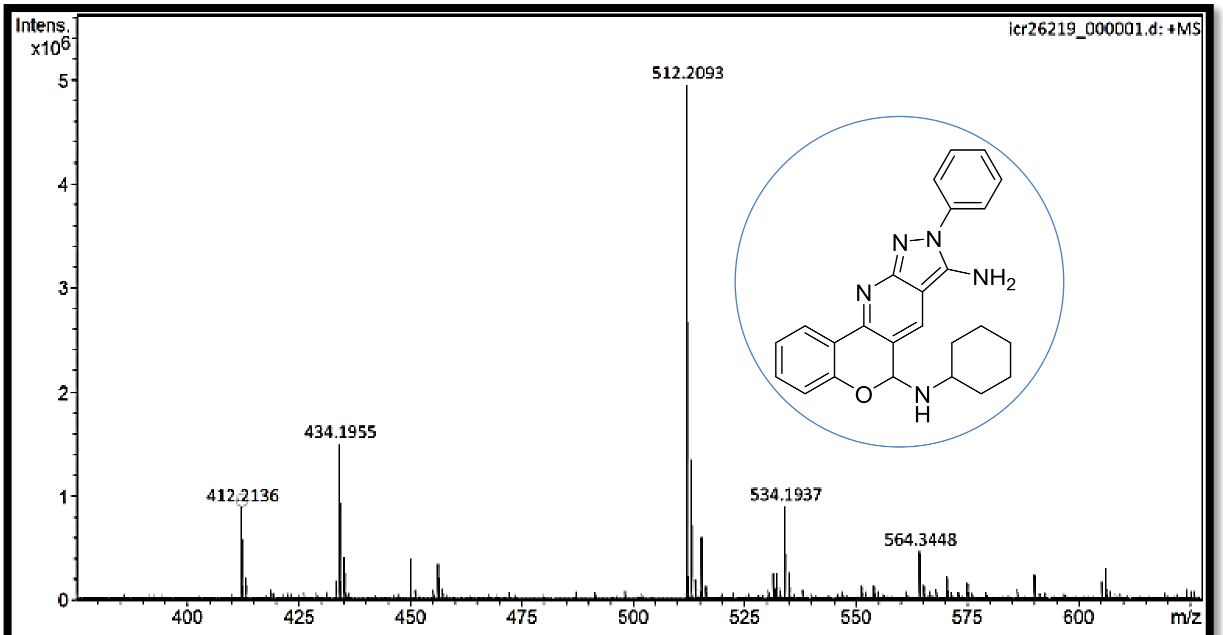

Meas. m/z	Ion Formula	m/z	err [ppm]	mSigma	rdb	e⁻ Conf	N-Rule
412.2136	C10H22N17O2	412.2137	0.3	n.a.	8.5	even	ok
	C11H28N10O7	412.2137	0.3	n.a.	3.0	odd	ok
	C25H26N5O	412.2132	-0.9	n.a.	15.5	even	ok

HR-Mass (ESI) (**4d**)

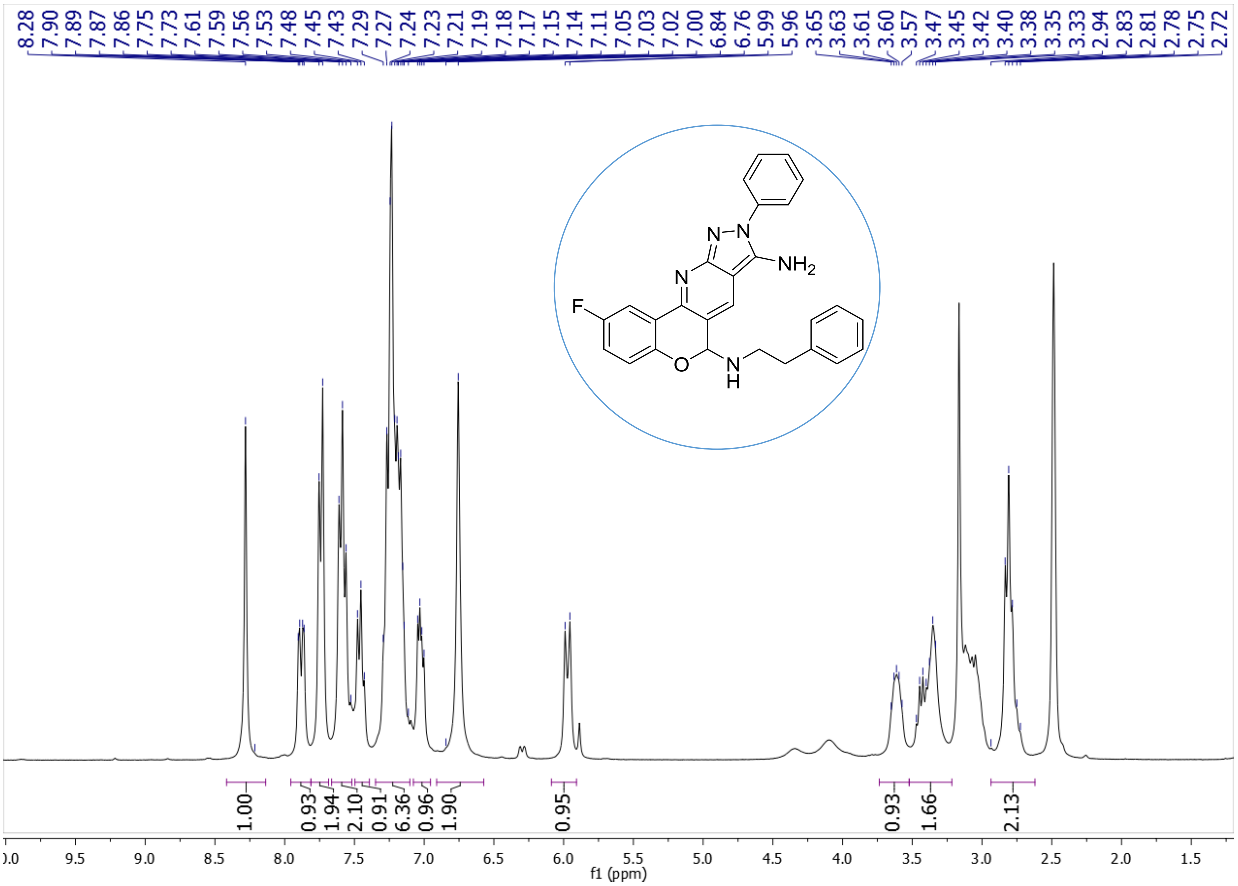

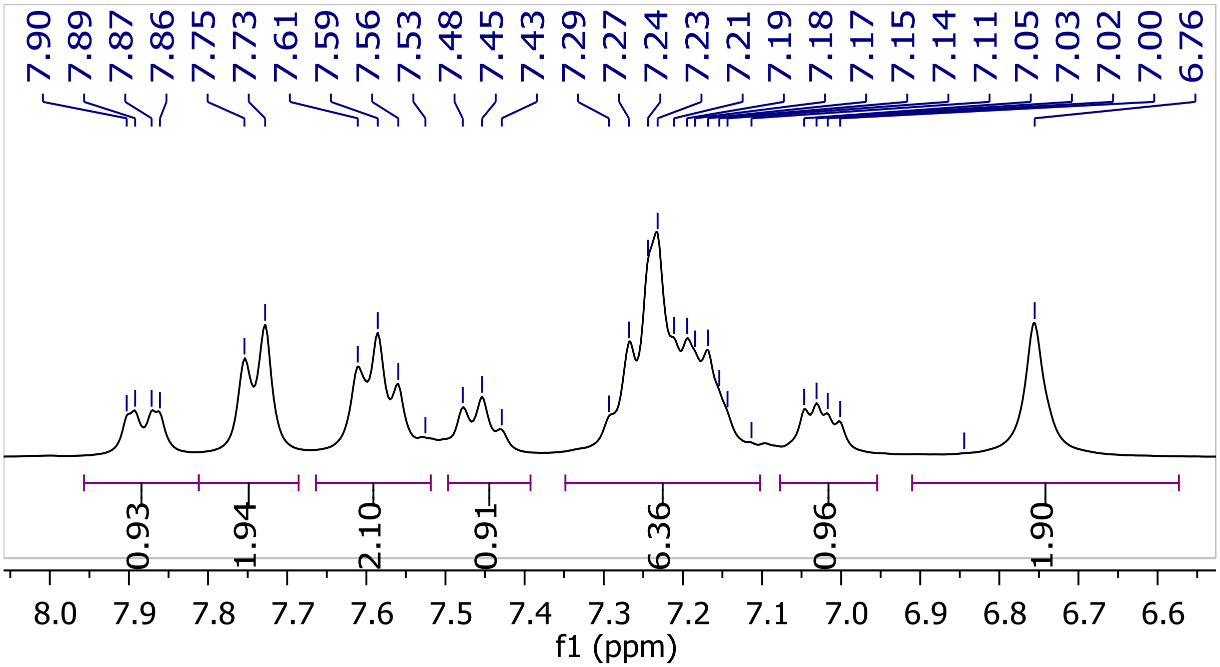

^{1}H-NMR （300 MHz, DMSO- d_6） (**4e**)

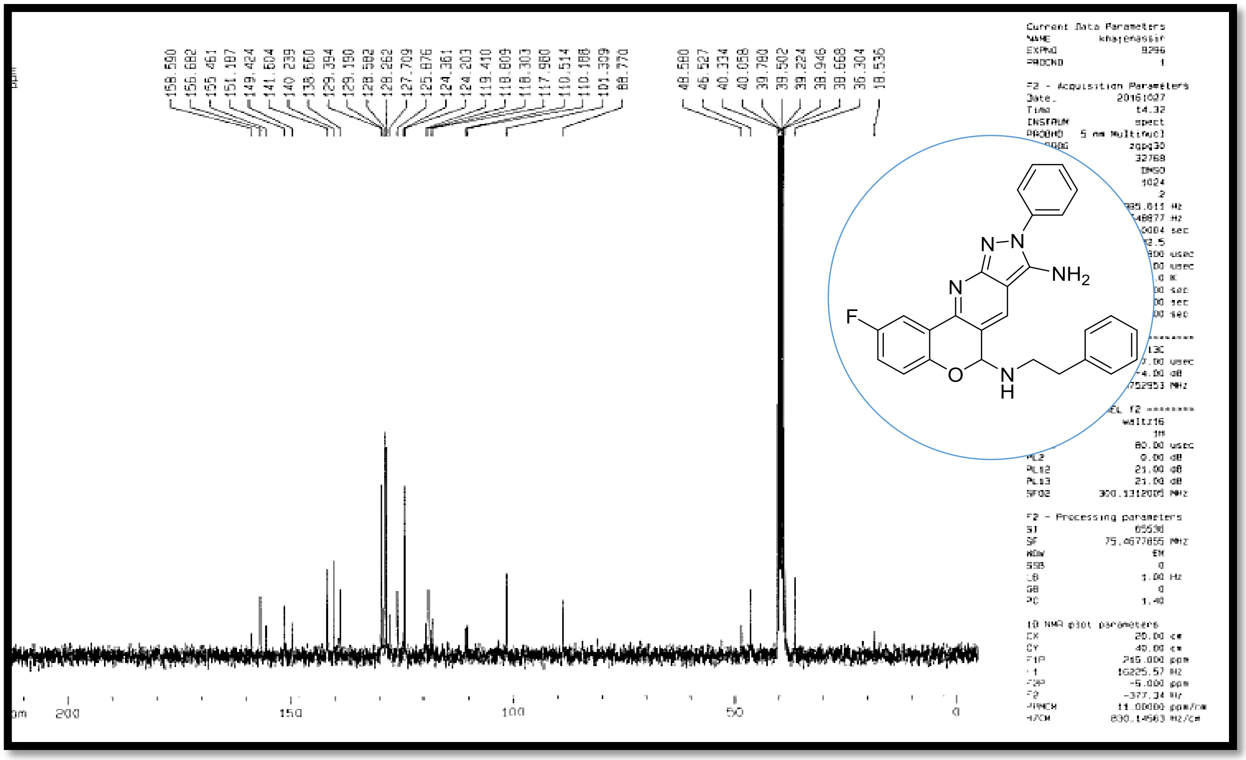

^{13}C-NMR（75 MHz, DMSO-d_6） (**4e**)

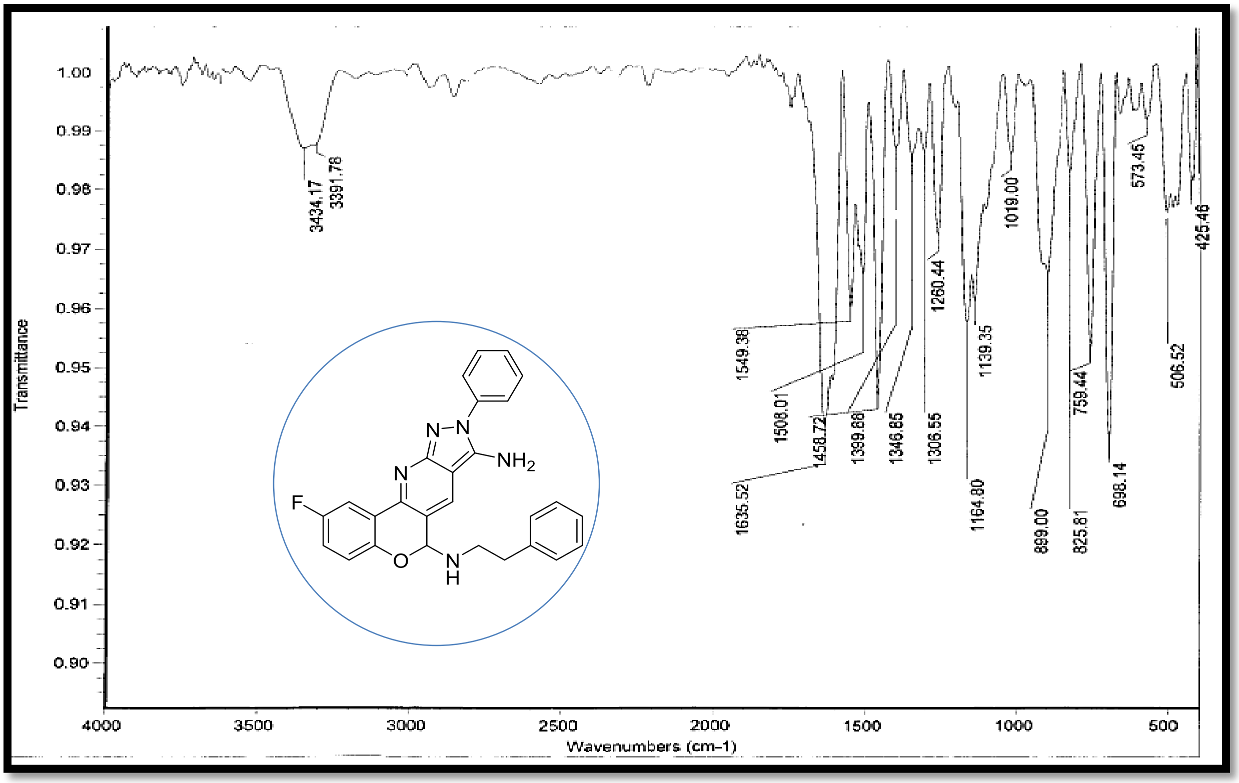

IR(KBr) (**4e**)

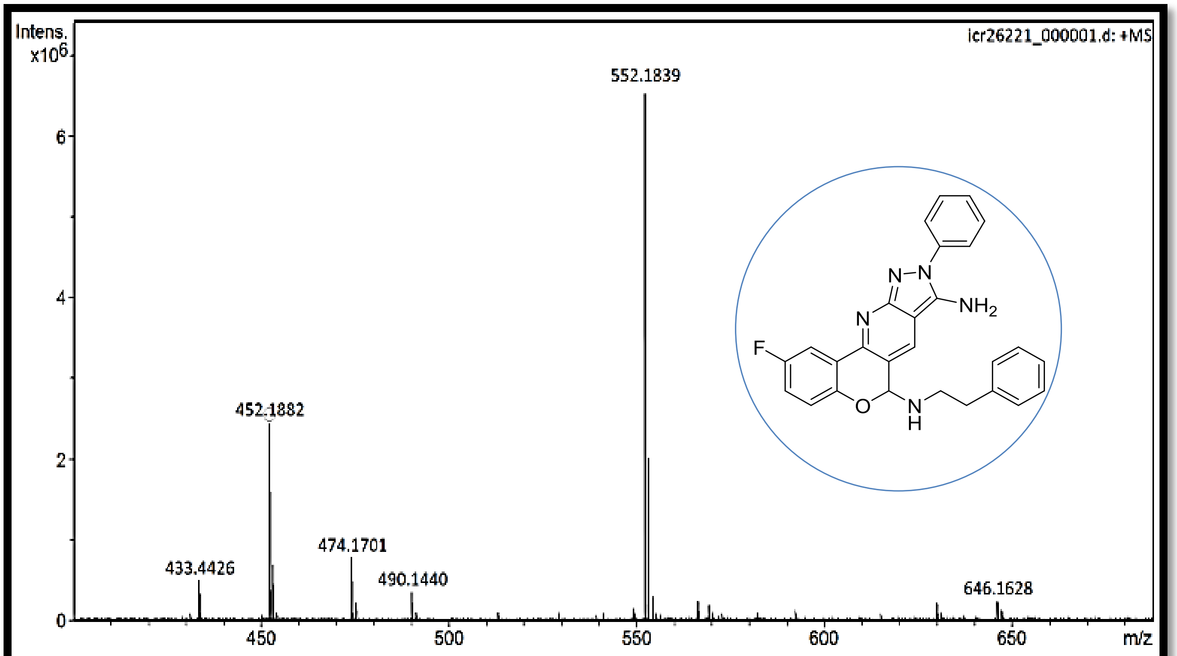

Meas. m/z	Ion Formula	m/z	err [ppm]	mSigma	rdb	e⁻ Conf	N-Rule
452.1882	C27H23FN5O	452.1881	-0.2	20.8	18.5	even	ok
	C19H32O12	452.1888	1.3	39.7	4.0	odd	ok
	C32H24N2O	452.1883	0.2	43.4	22.0	odd	ok

HR-Mass (ESI) (**4e**)

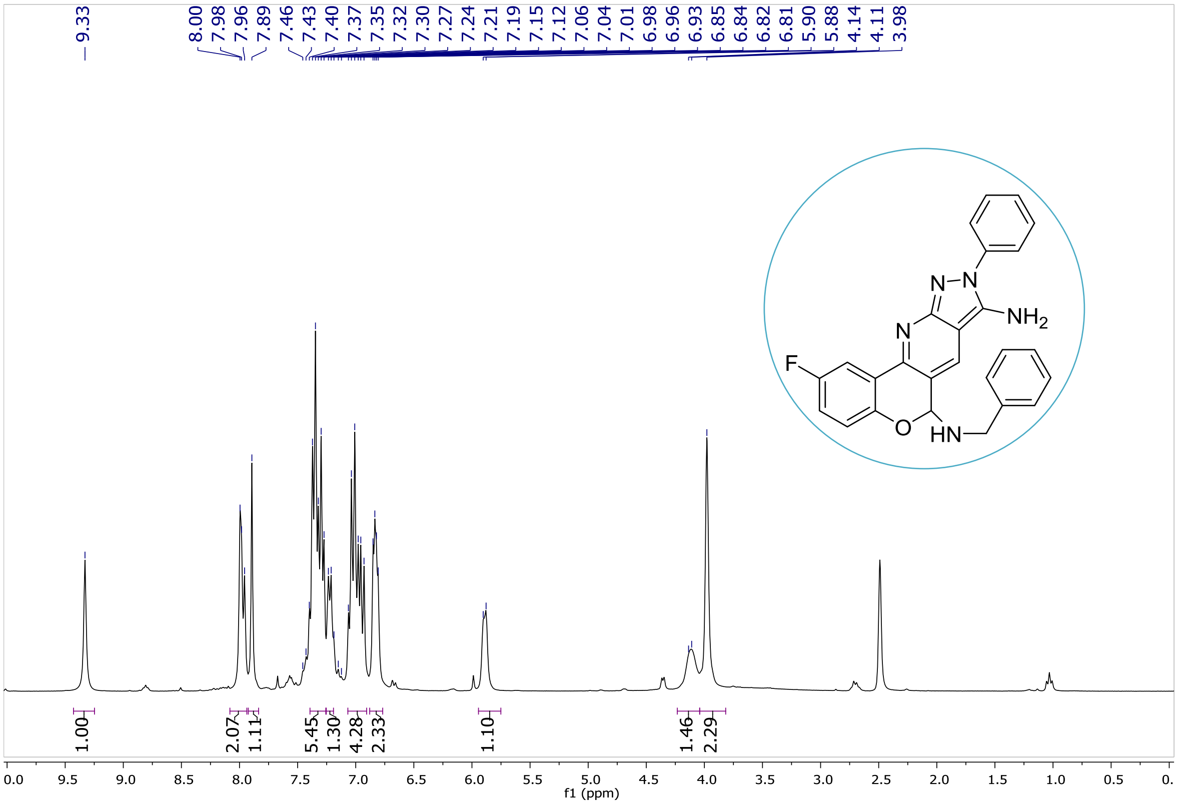

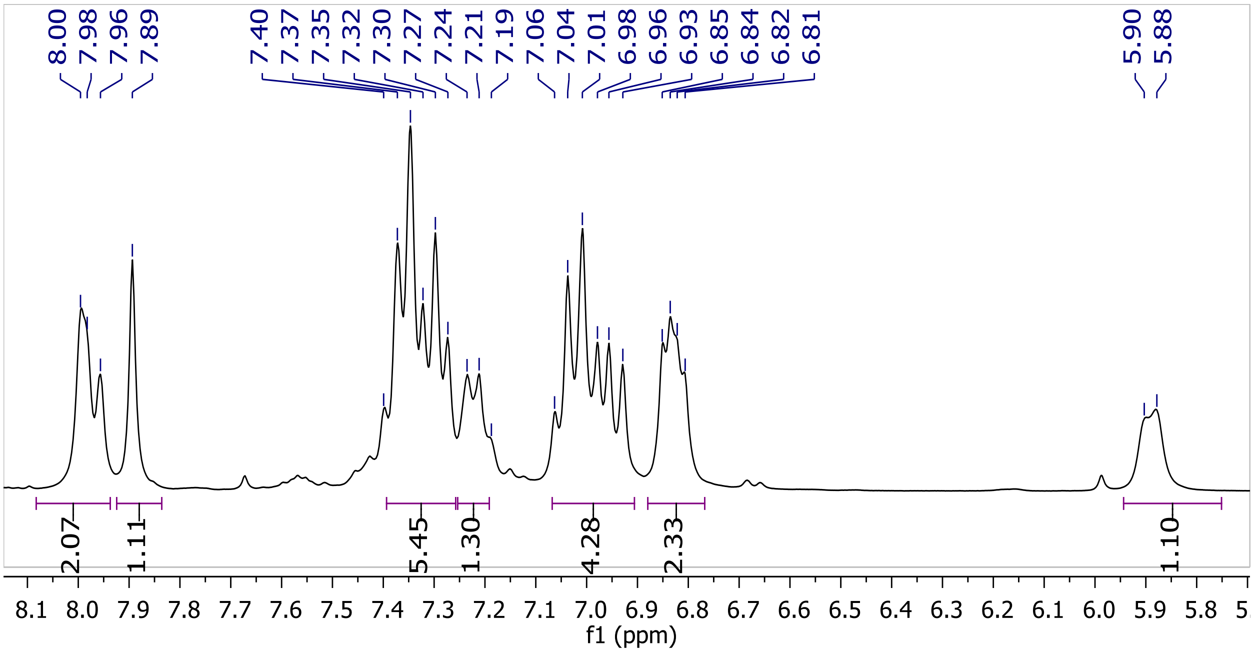

^{1}H-NMR （300 MHz, DMSO- d_6） (**4f**)

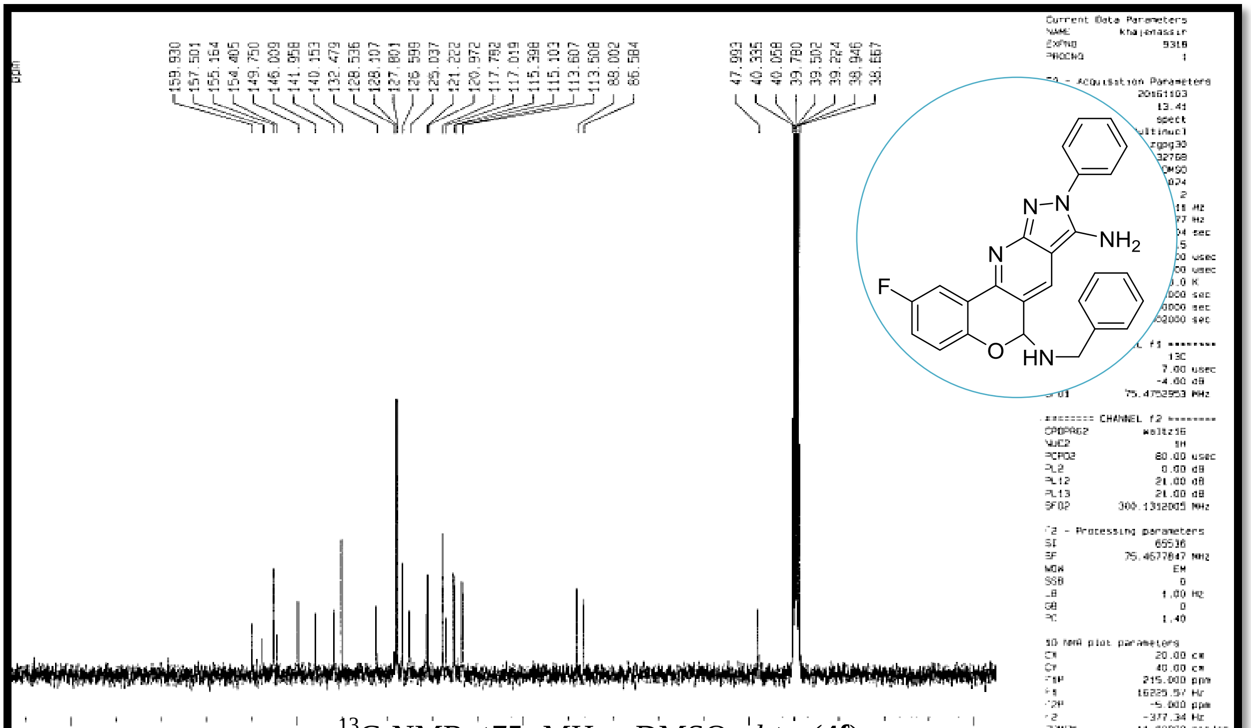

^{13}C-NMR（75 MHz, DMSO- d_6） (**4f**)

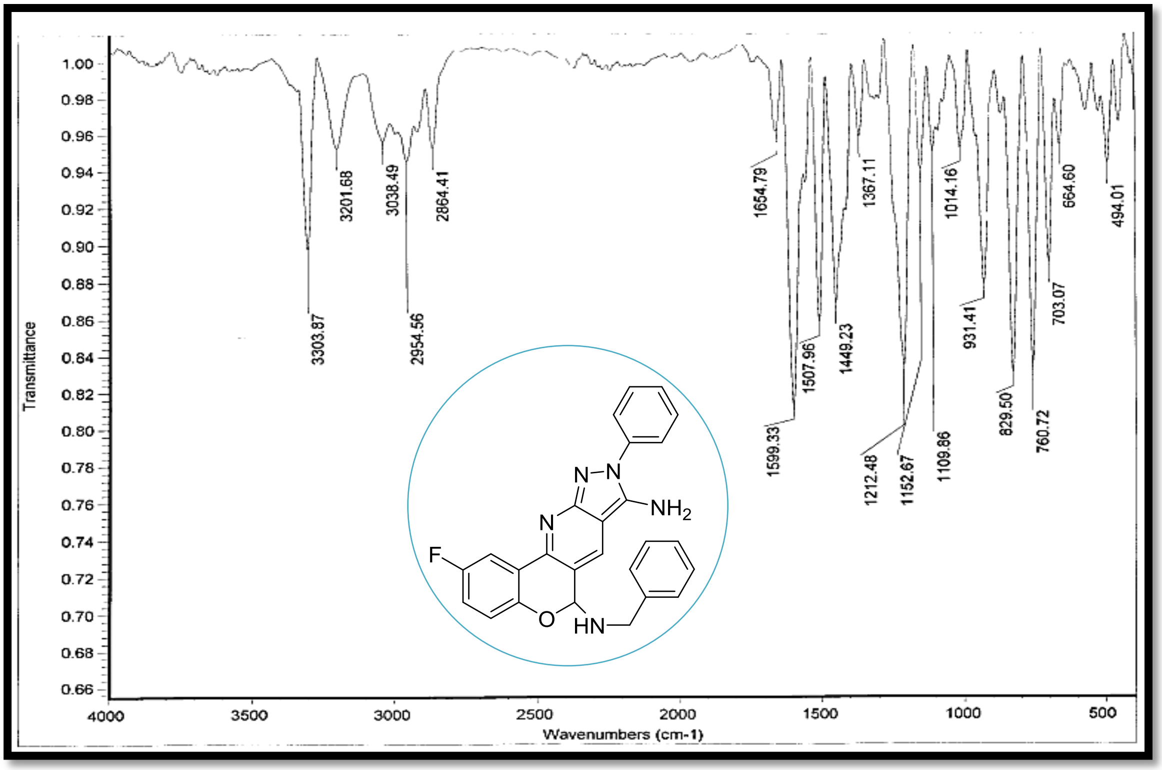

IR (KBr) (**4f**)

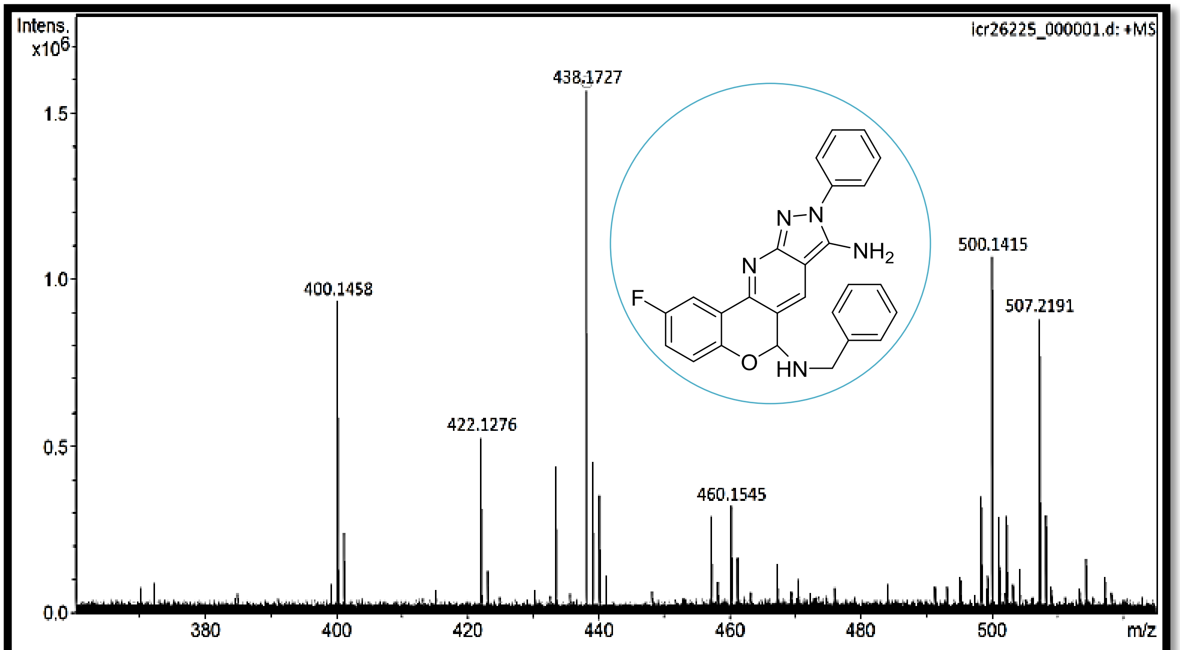

Meas. m/z	Ion Formula	m/z	err [ppm]	mSigma	rdb	e⁻ Conf	N-Rule
438.1727	C26H21FN5O	438.1725	-0.5	21.7	18.5	even	ok
	C31H22N2O	438.1727	-0.0	35.2	22.0	odd	ok

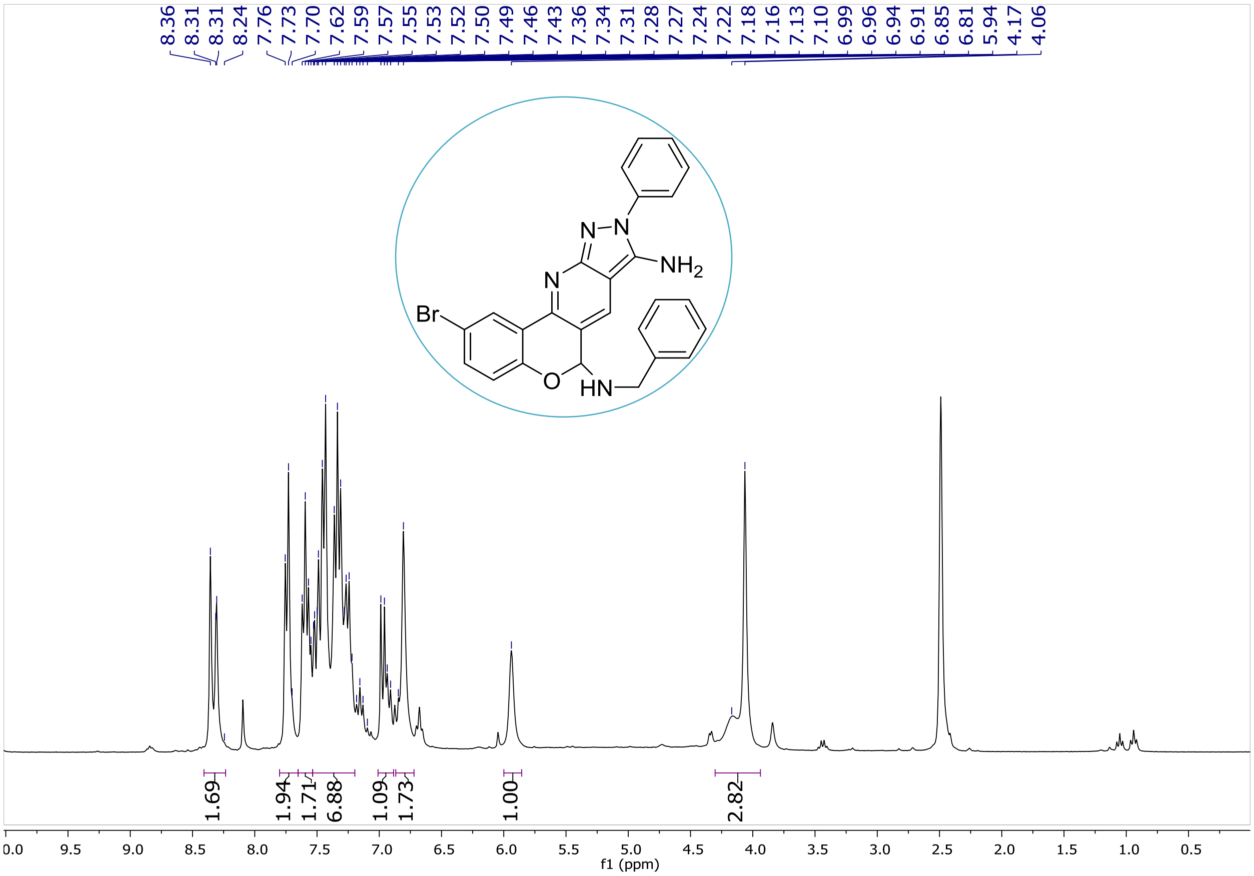

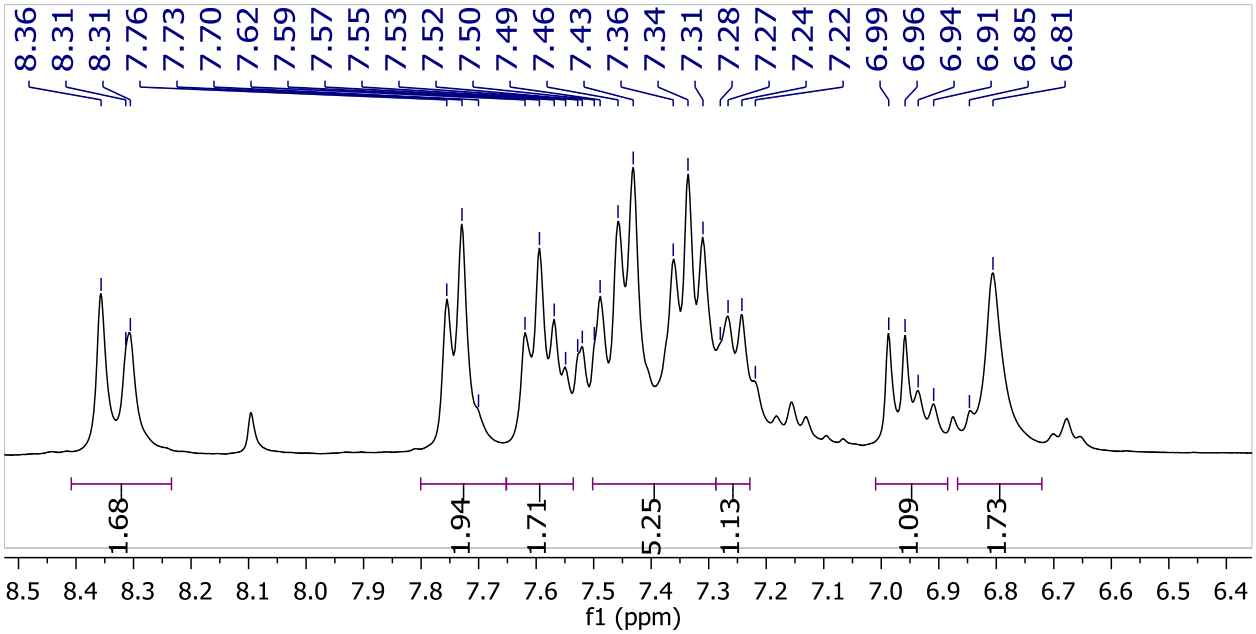

^{1}H-NMR （300 MHz, DMSO- d_6） (**4g**)

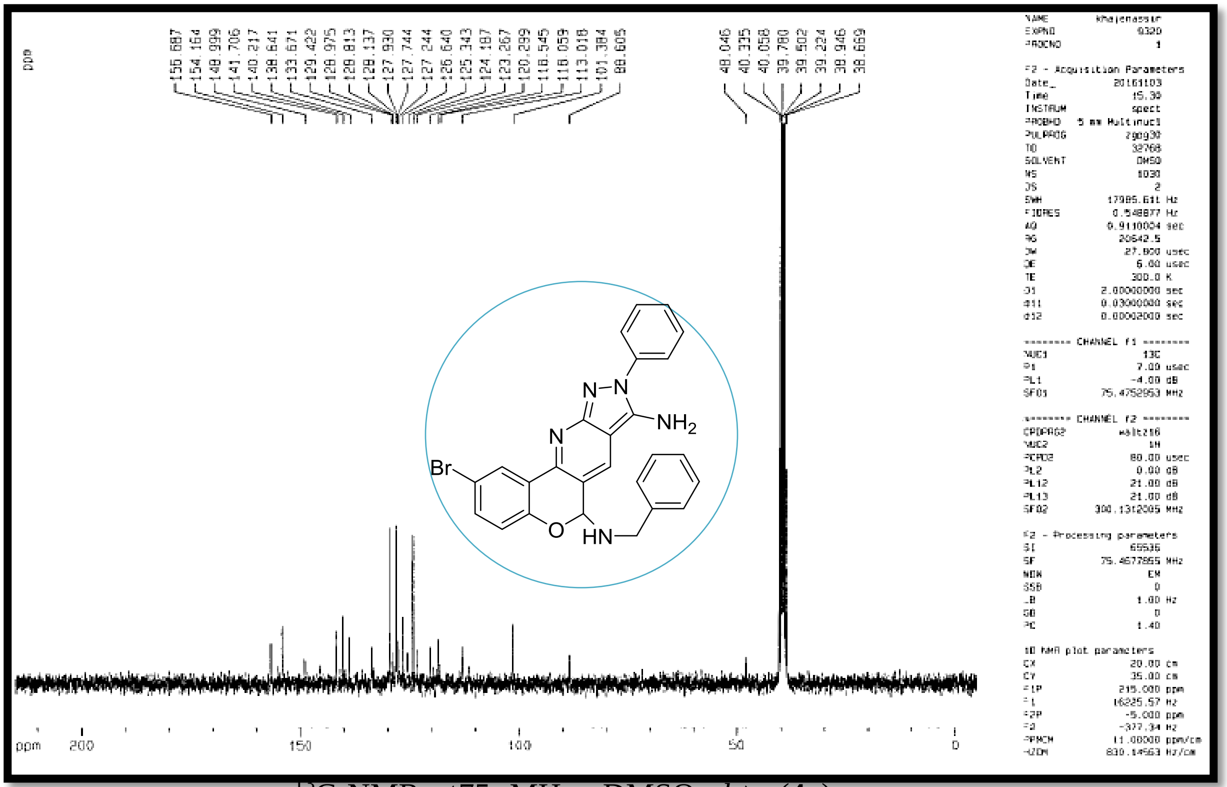

^{13}C-NMR (75 MHz, DMSO-d_6) (**4g**)

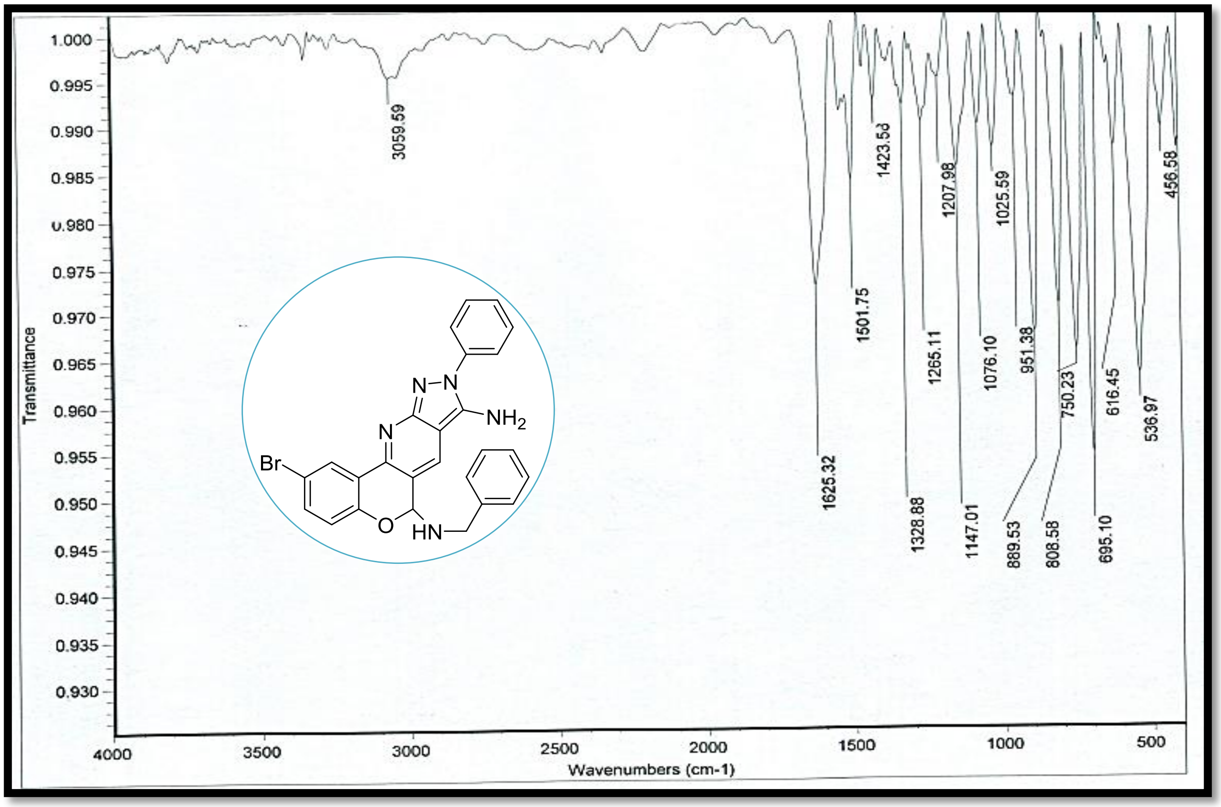

IR (KBr) (**4g**)

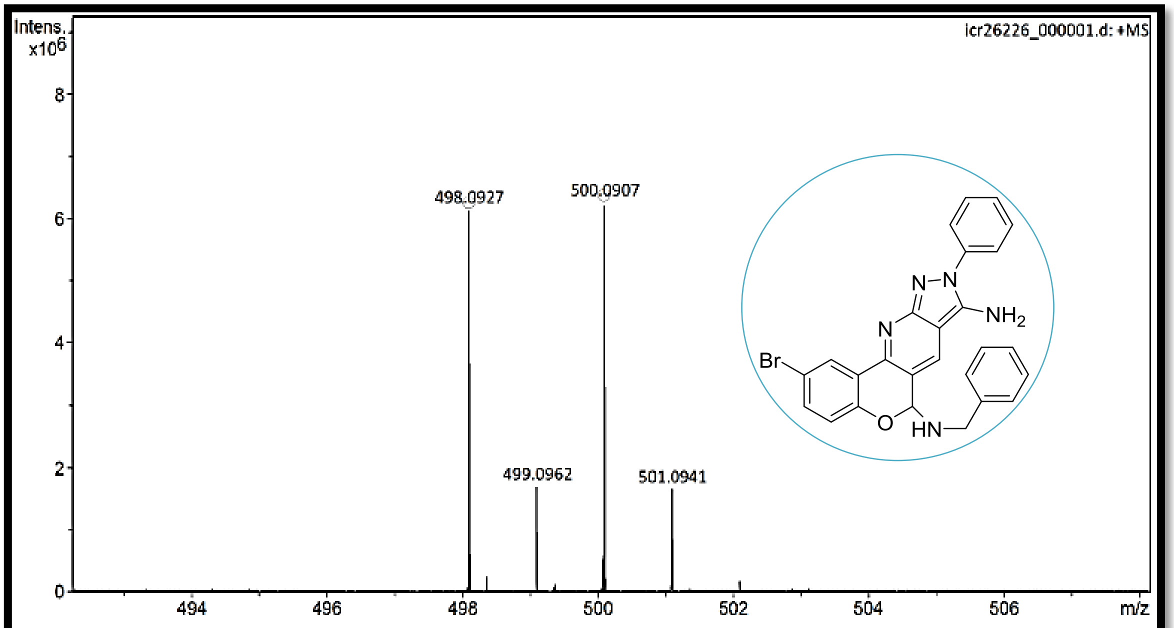

Meas. m/z	Ion Formula	m/z	err [ppm]	mSigma	rdb	e⁻ Conf	N-Rule
498.0927	C26H16N3O8	498.0932	1.0	440.3	20.5	even	ok
	C26H21N5O^79Br	498.0924	-0.6	481.1	18.5	even	ok
500.0907	C26H21N5O^81Br	500.0904	-0.7	22.4	18.5	even	ok
	C28H23N2O2^81Br	500.0917	2.0	26.1	18.0	odd	ok
	C32H12N4O3	500.0904	-0.6	41.2	29.0	odd	ok
	C34H14NO4	500.0917	2.1	54.8	28.5	even	ok

HR-Mass (ESI) (**4g**)

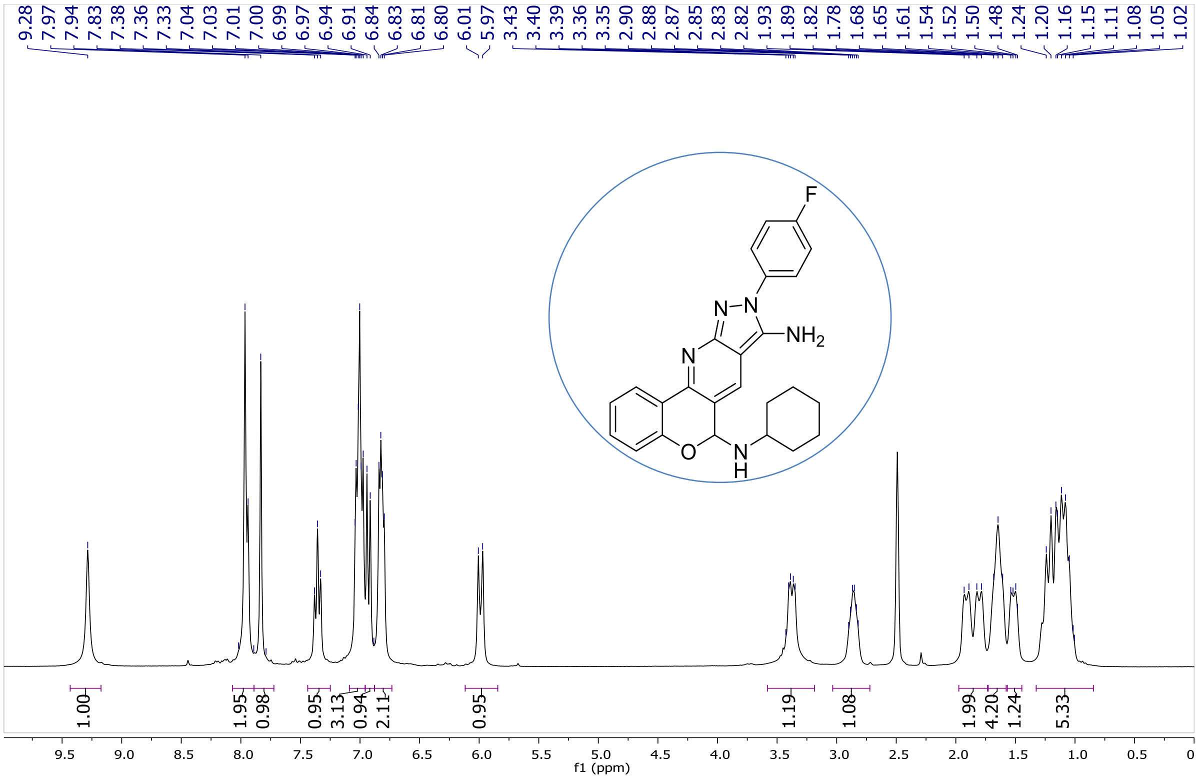

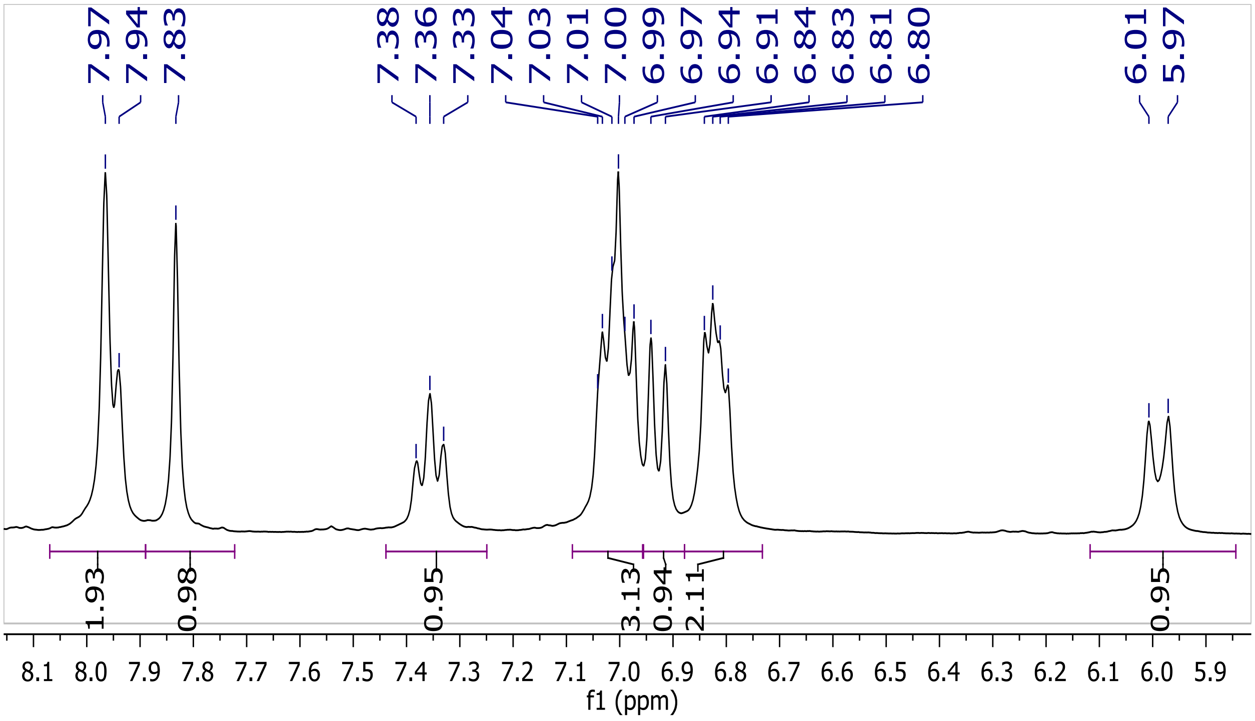

^{1}H-NMR （300 MHz, DMSO- d_6） (**4h**)

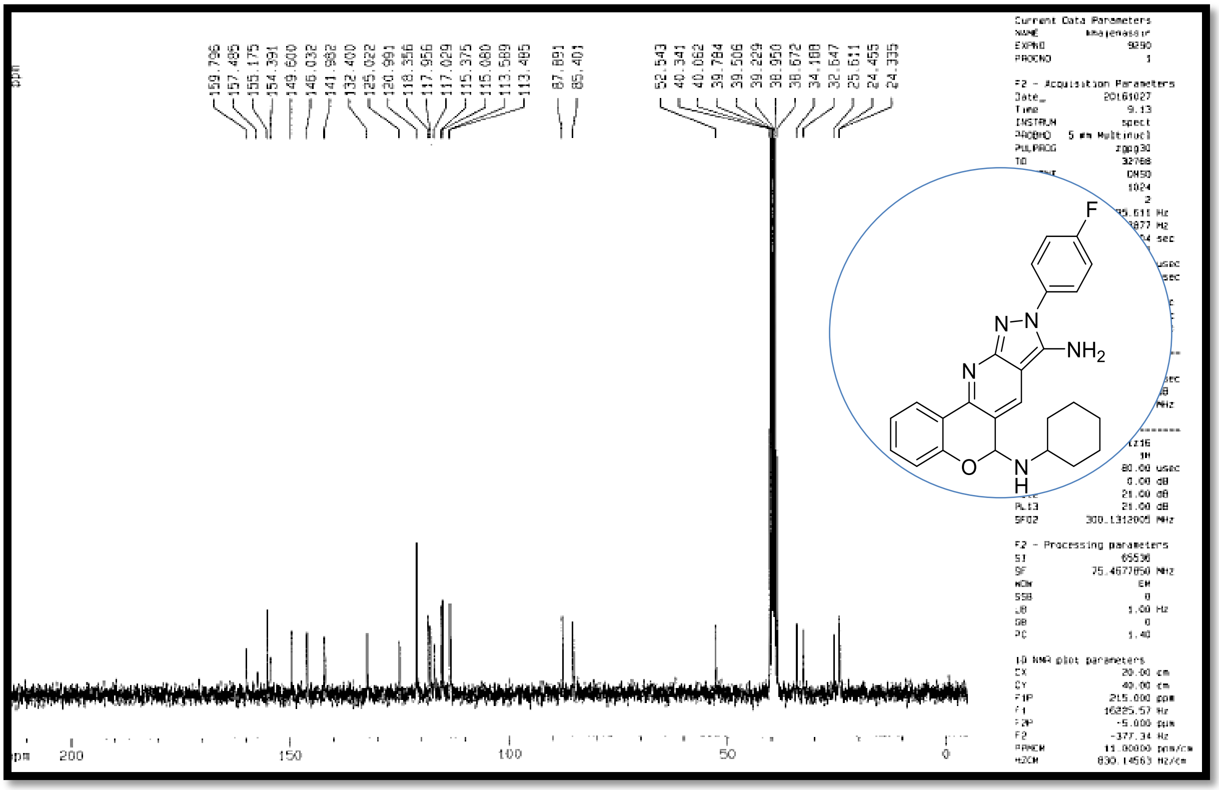

^{13}C-NMR （75 MHz, DMSO- d_6） (**4h**)

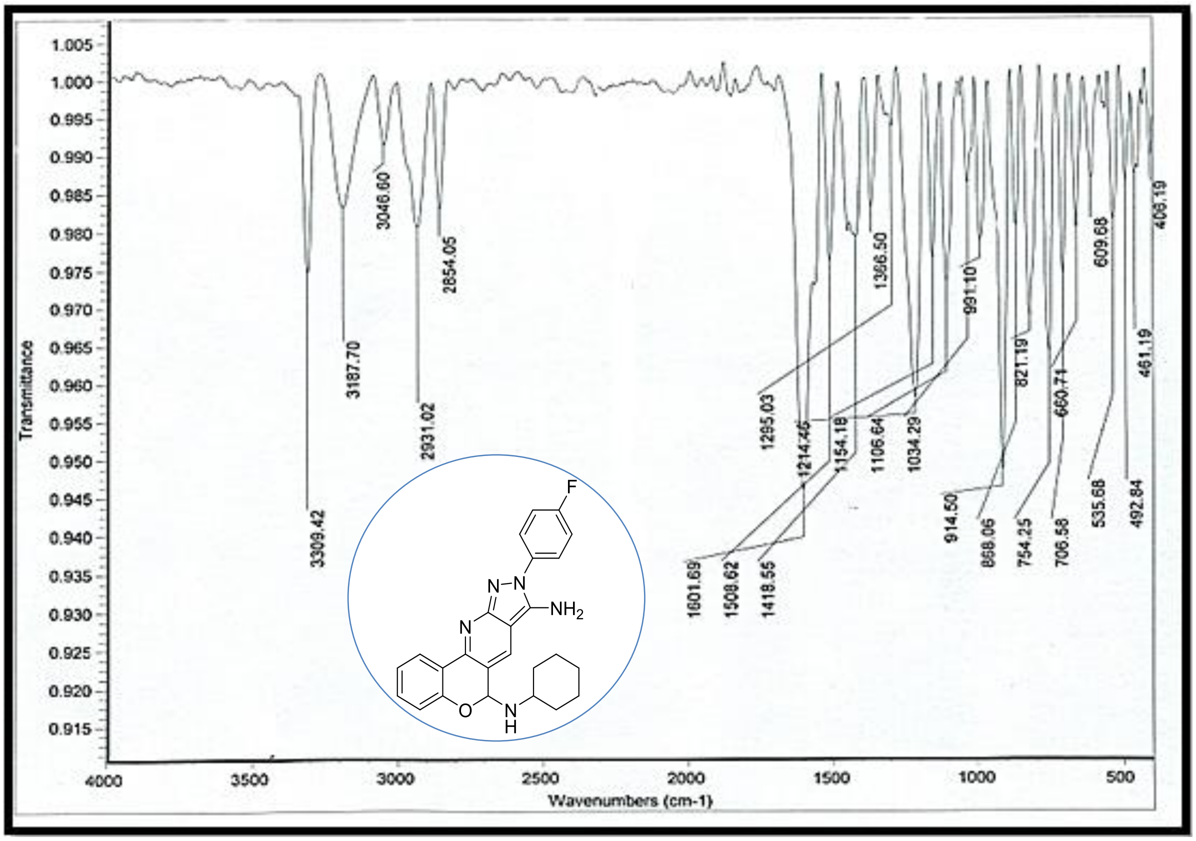

IR (KBr) (**4h**)

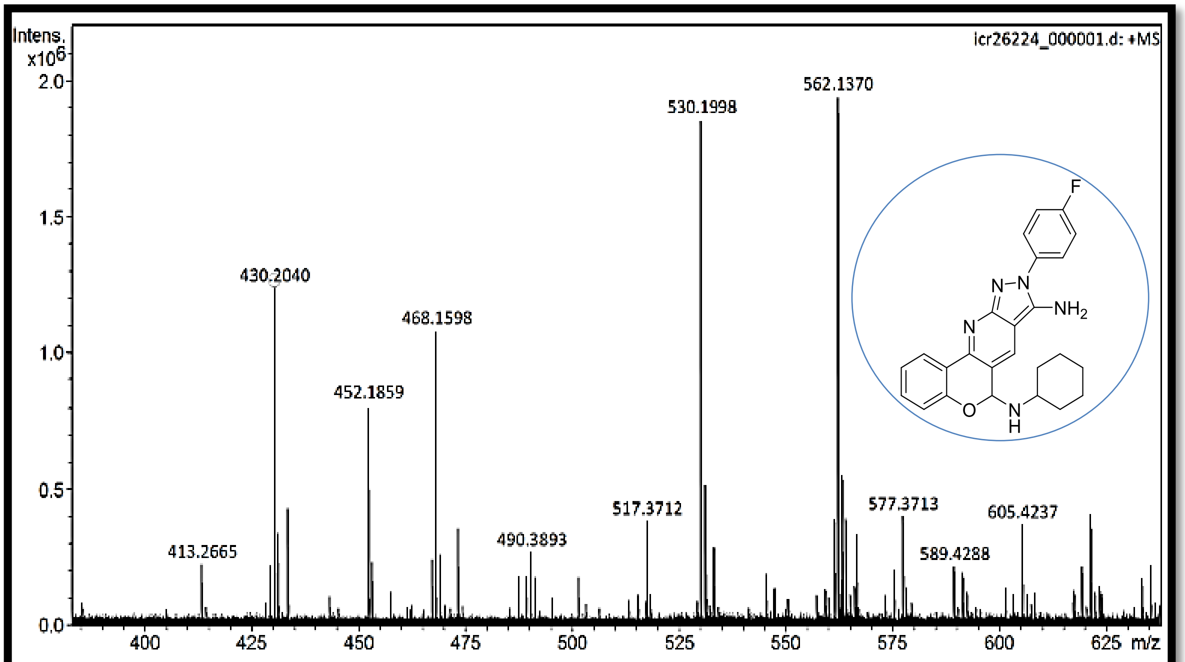

Meas. m/z	Ion Formula	m/z	err [ppm]	mSigma	rdb	e⁻ Conf	N-Rule
430.2040	C25H25FN5O	430.2038	-0.6	20.1	15.5	even	ok
	C30H26N2O	430.2040	-0.1	35.0	19.0	odd	ok

HR-Mass (ESI) (**4h**)

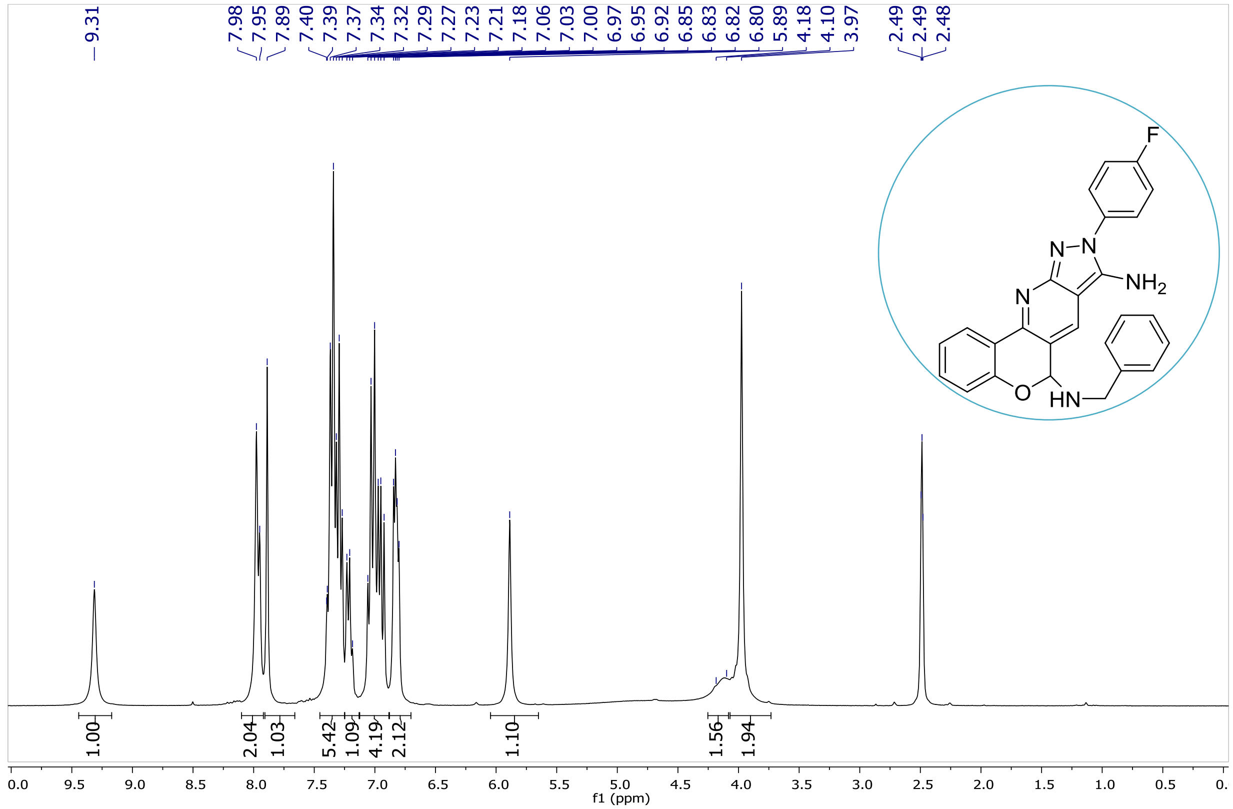

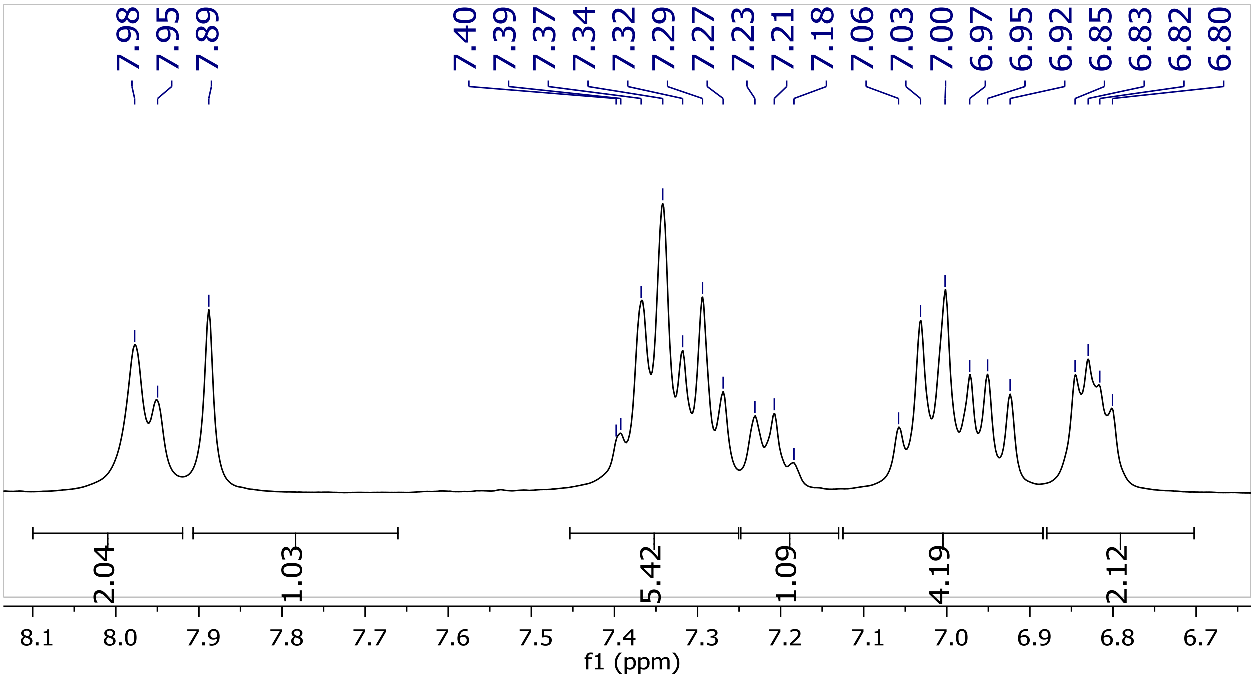

^{1}H-NMR (300 MHz, DMSO- d_6) (**4i**)

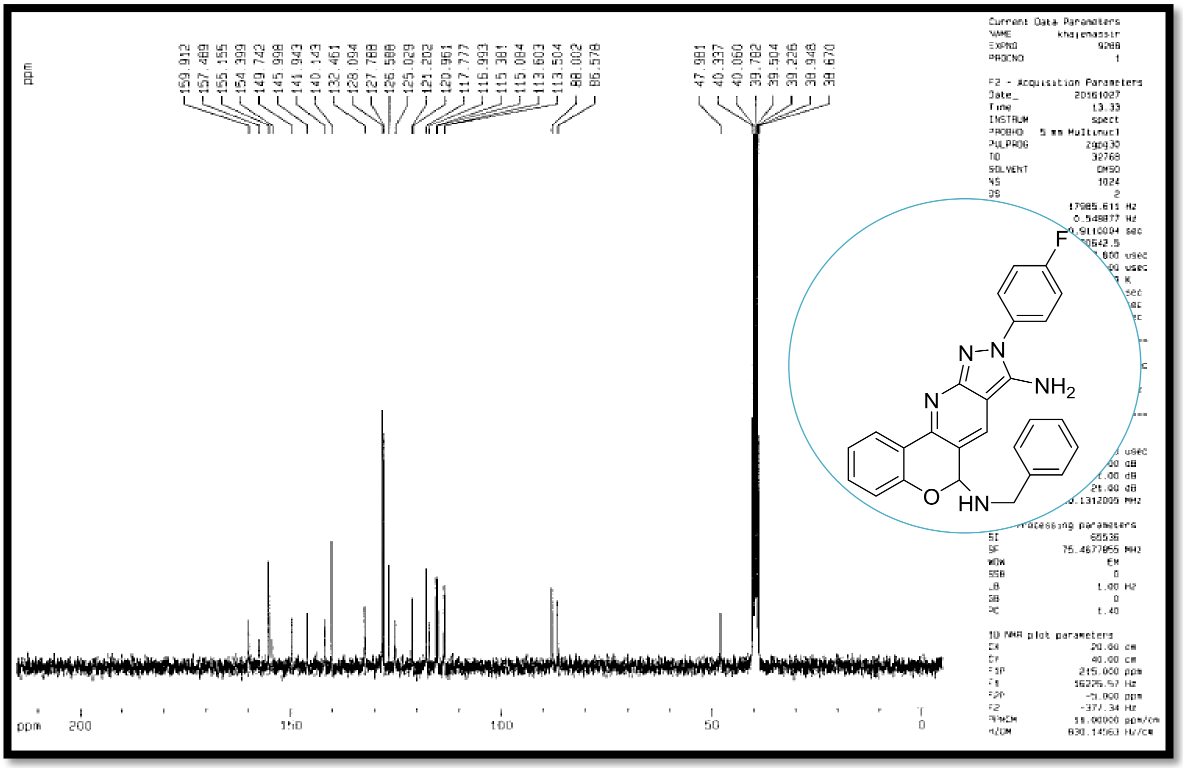

^{13}C-NMR （75 MHz, DMSO- d_6） (**4i**)

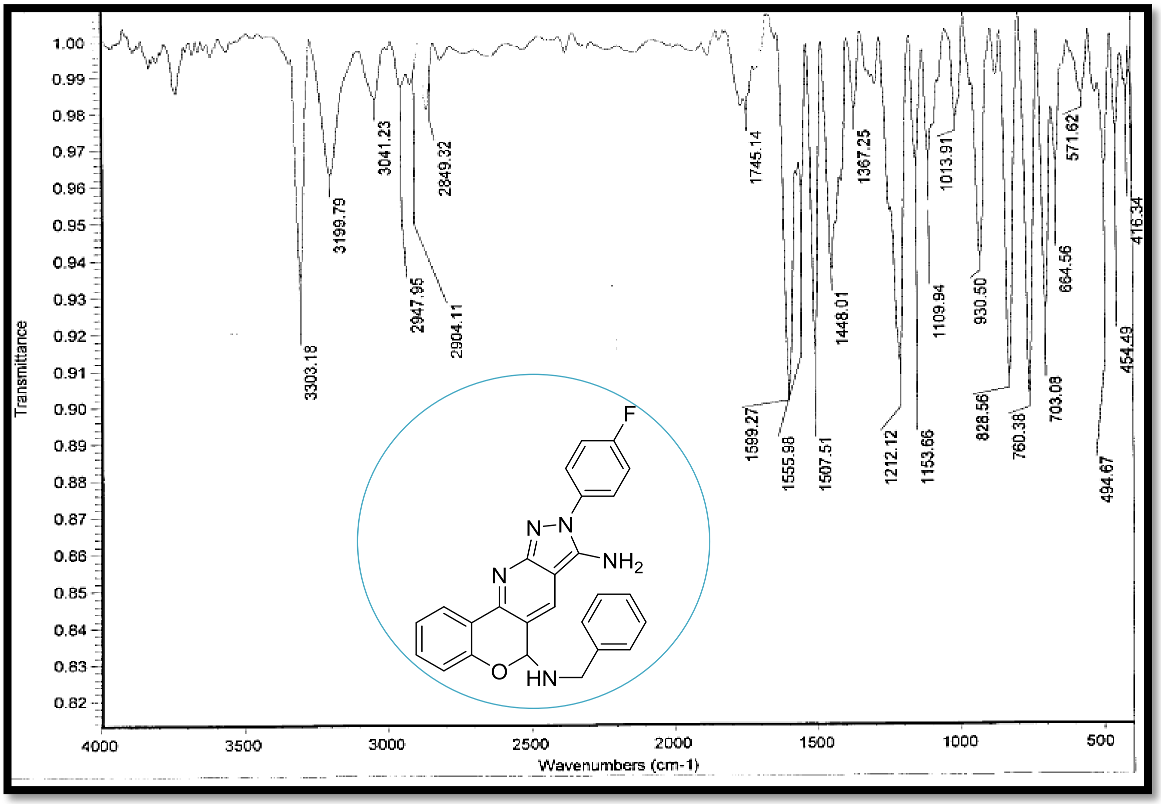

IR (KBr) (**4i**)

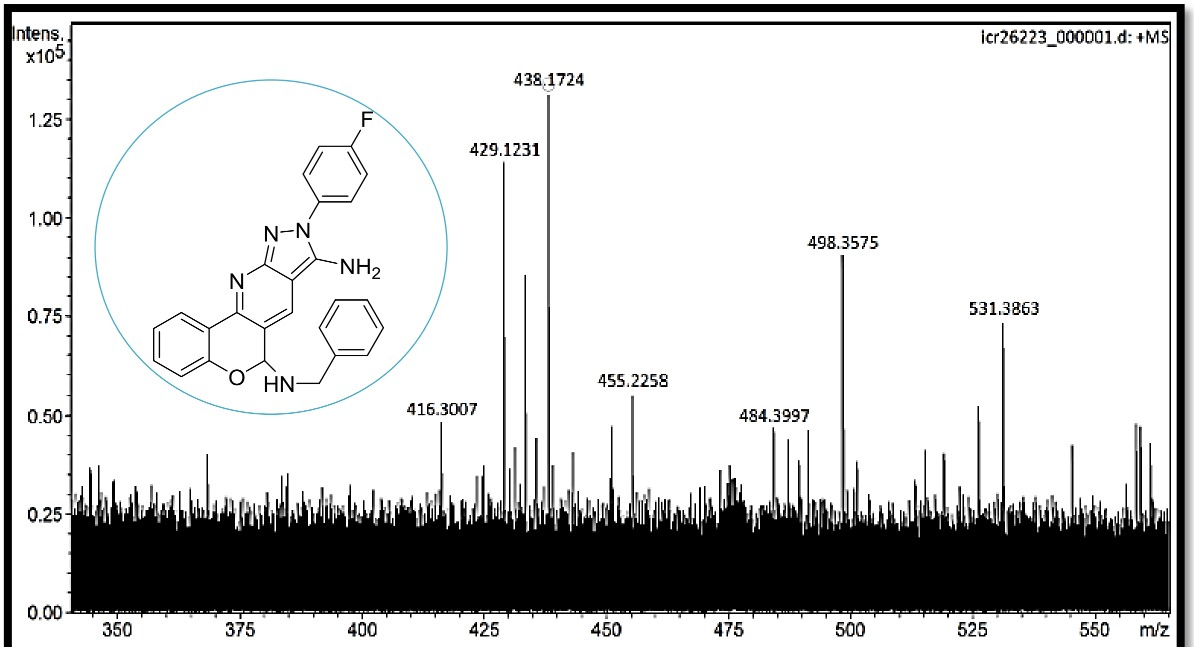

Meas. m/z	Ion Formula	m/z	err [ppm]	mSigma	rdb	e⁻ Conf	N-Rule
438.1724	C26H21FN5O	438.1725	0.2	n.a.	18.5	even	ok
	C31H22N2O	438.1727	0.6	n.a.	22.0	odd	ok
	C29H20N5	438.1713	-2.5	n.a.	22.5	even	ok
	C28H23FN2O2	438.1738	3.2	n.a.	18.0	odd	ok
	C28H24NO4	438.1700	-5.5	n.a.	17.5	even	ok

HR-Mass (ESI) (**4i**)

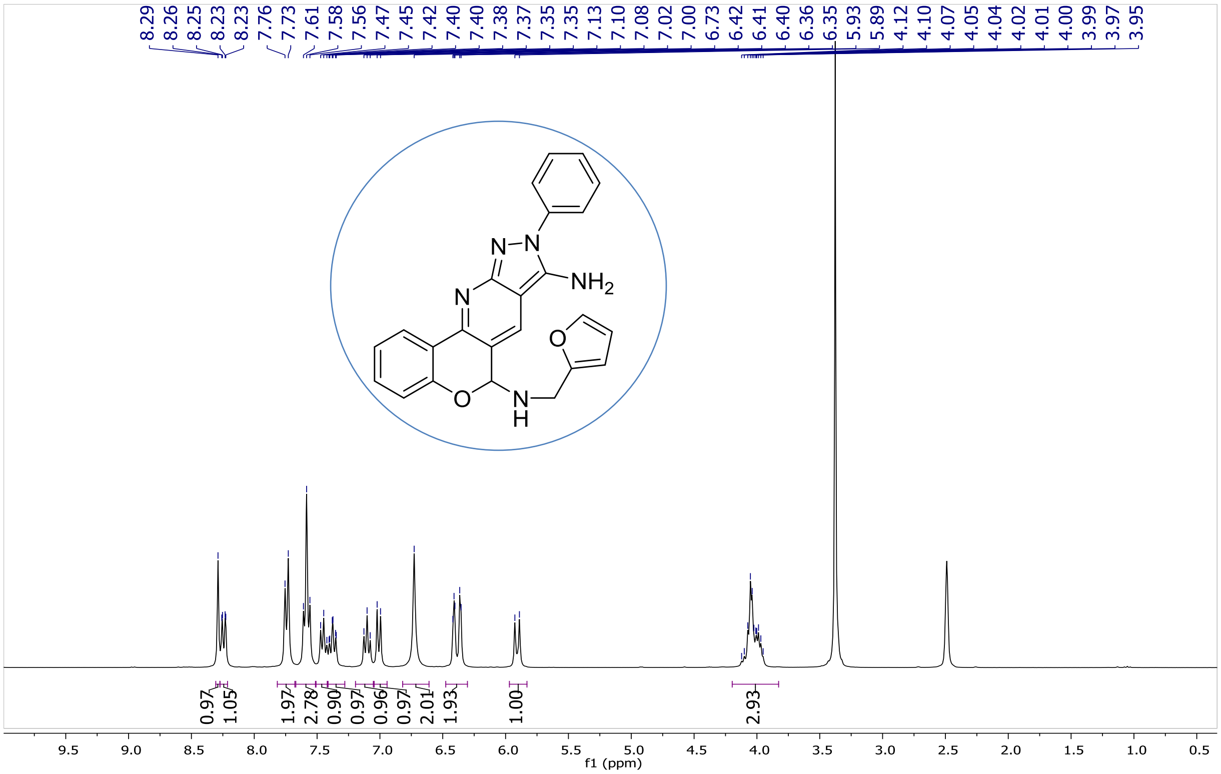

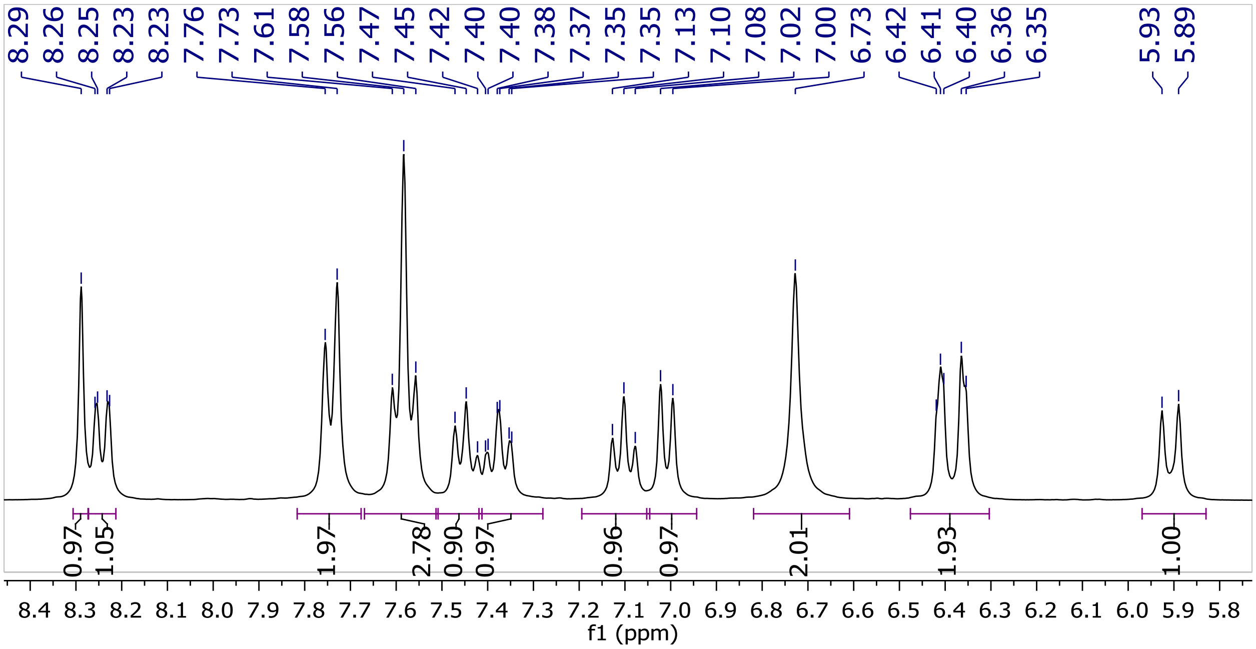

^{1}H-NMR （300 MHz, DMSO- d_6） (**4j**)

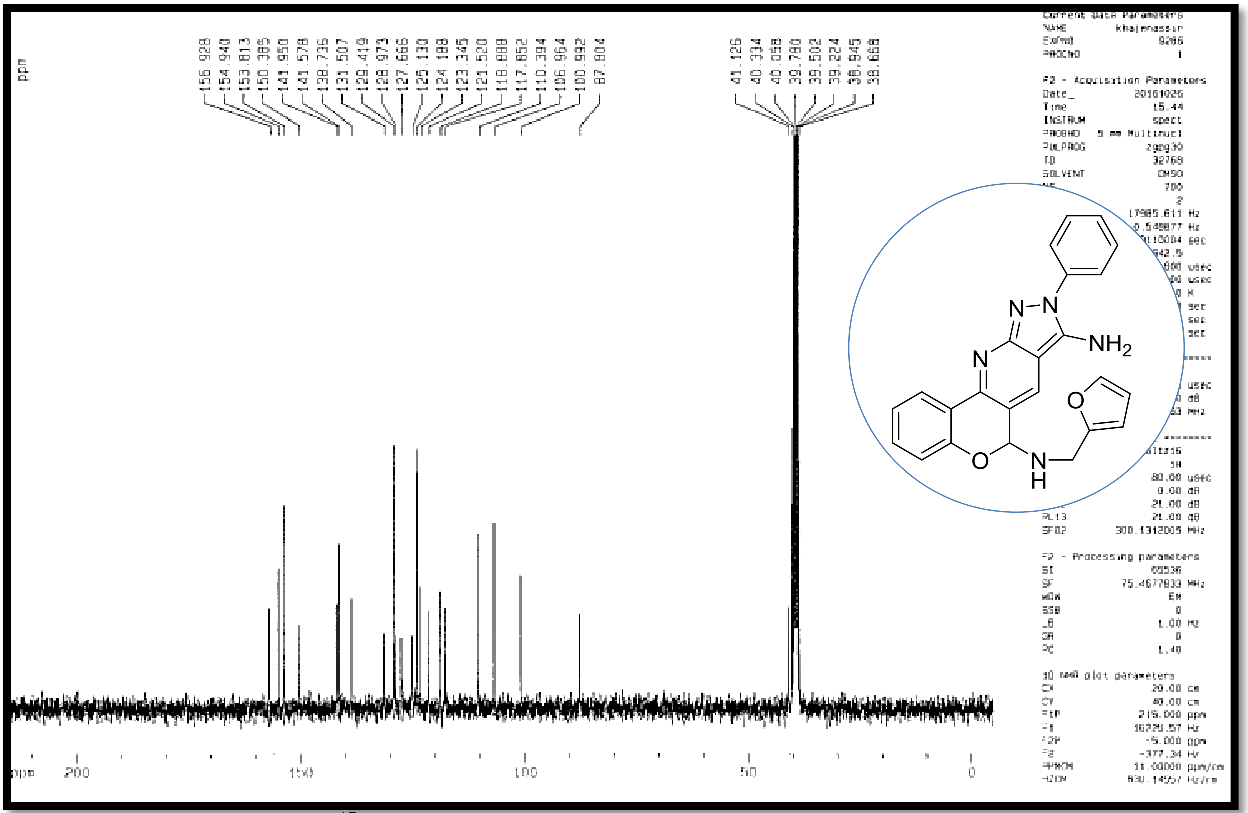

^{13}C-NMR（75 MHz, DMSO- d_6） (**4j**)

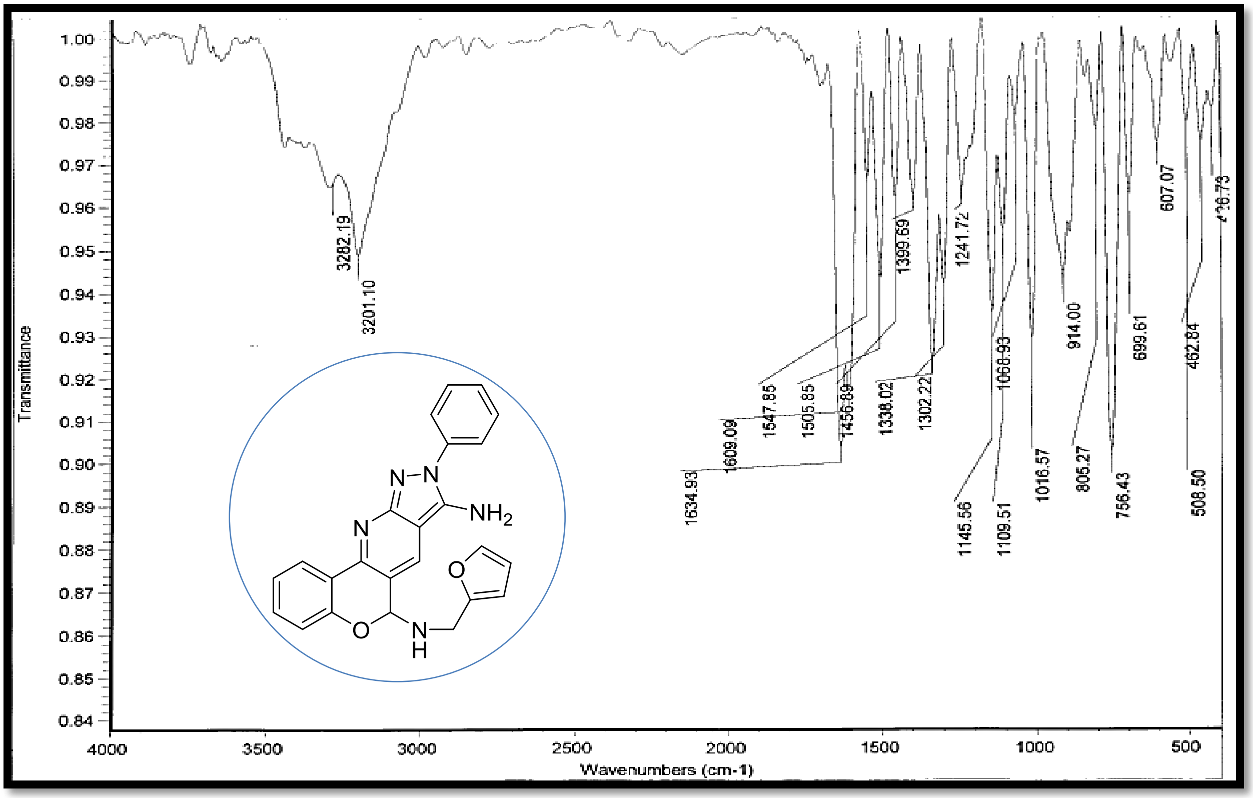

IR (KBr) (**4j**)

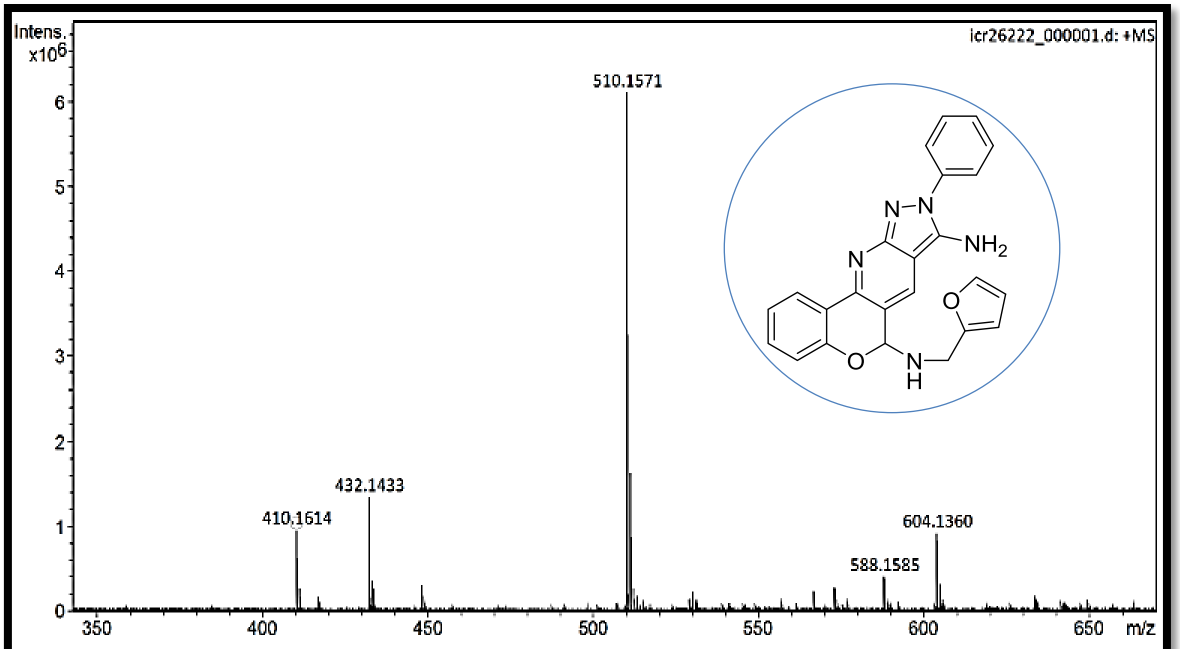

Meas. m/z	Ion Formula	m/z	err [ppm]	mSigma	rdb	e⁻ Conf	N-Rule
410.1614	C24H20N5O2	410.1612	-0.6	n.a.	17.5	even	ok
	C9H16N17O3	410.1617	0.6	n.a.	10.5	even	ok
	C10H22N10O8	410.1617	0.6	n.a.	5.0	odd	ok
	C11H28N3O13	410.1617	0.7	n.a.	-0.5	even	ok

HR-Mass (ESI) (**4j**)

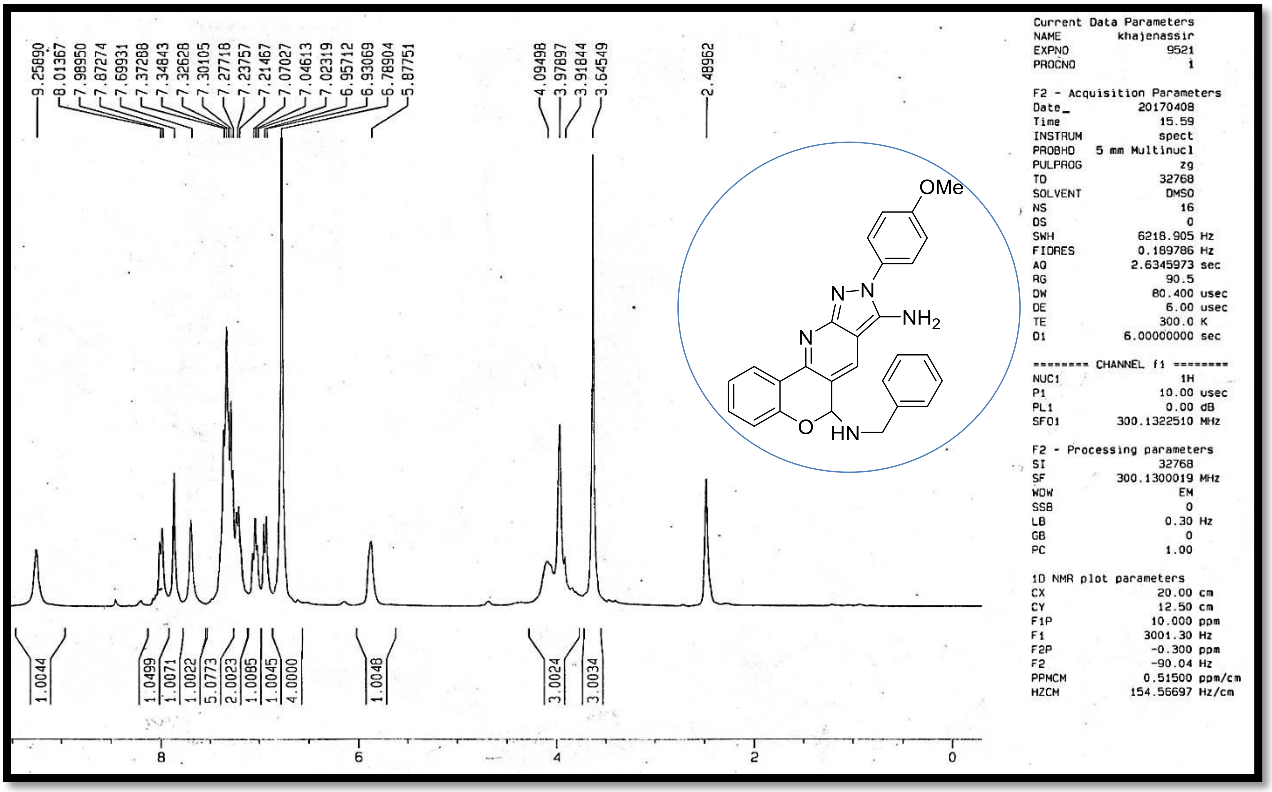

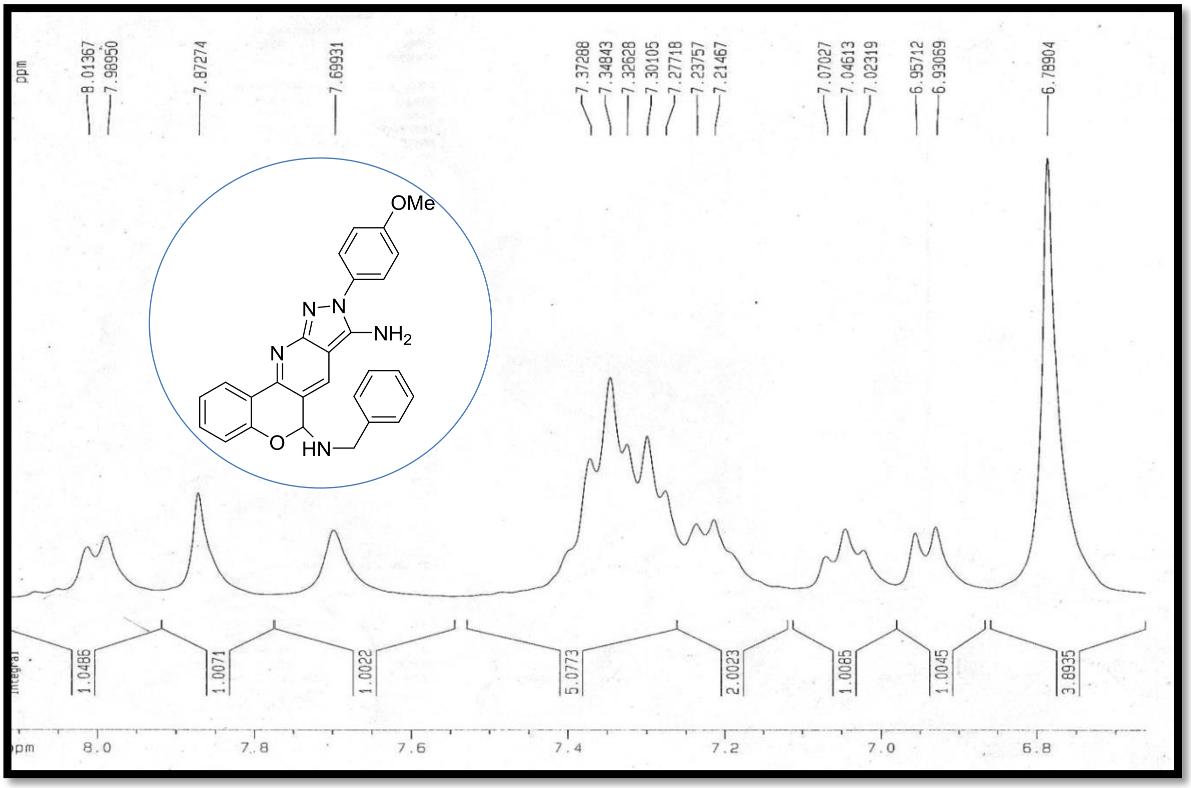

^{1}H-NMR (300 MHz, DMSO-d_6) (**4k**)

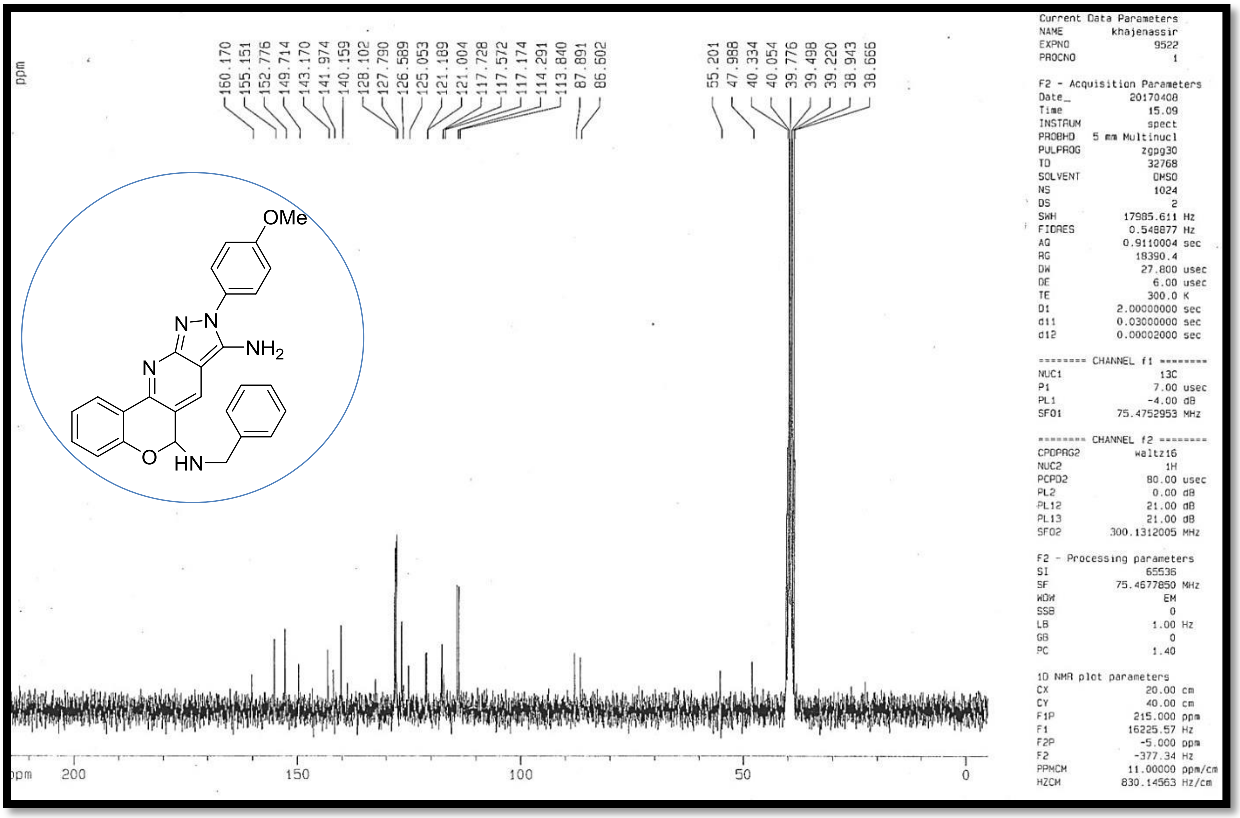

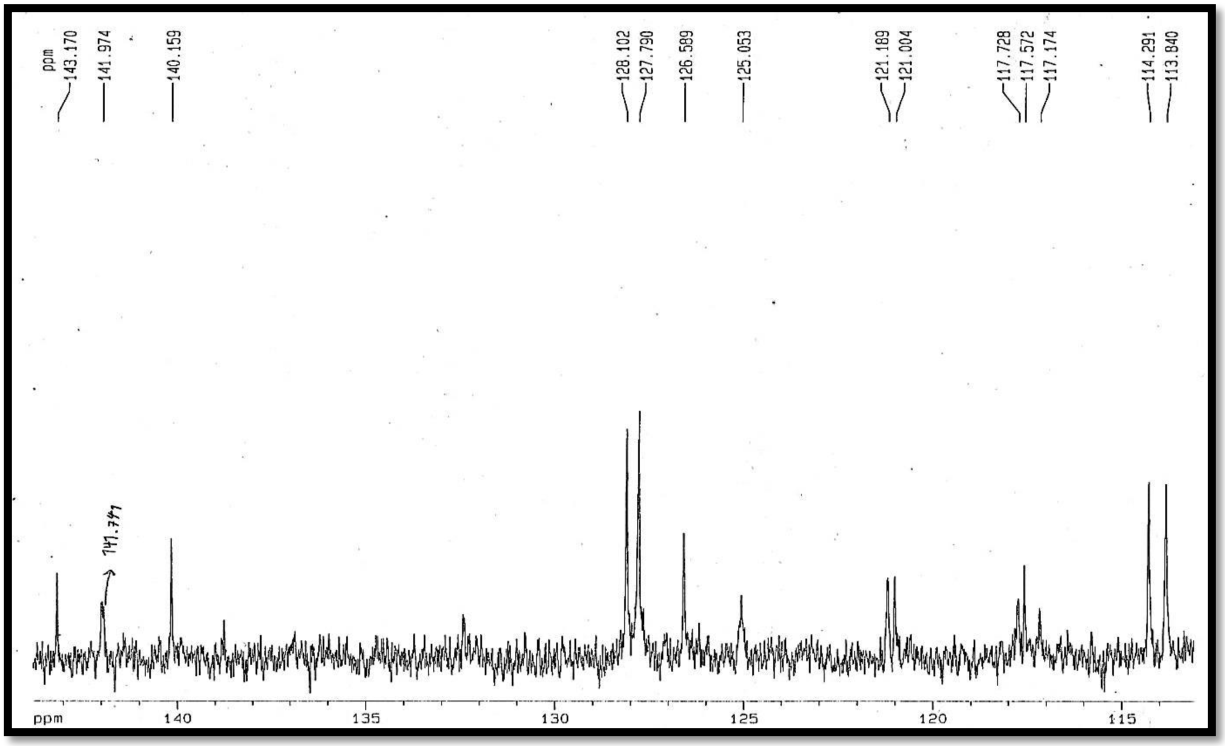

^{13}C-NMR（75 MHz, DMSO-d_6）(**4k**)

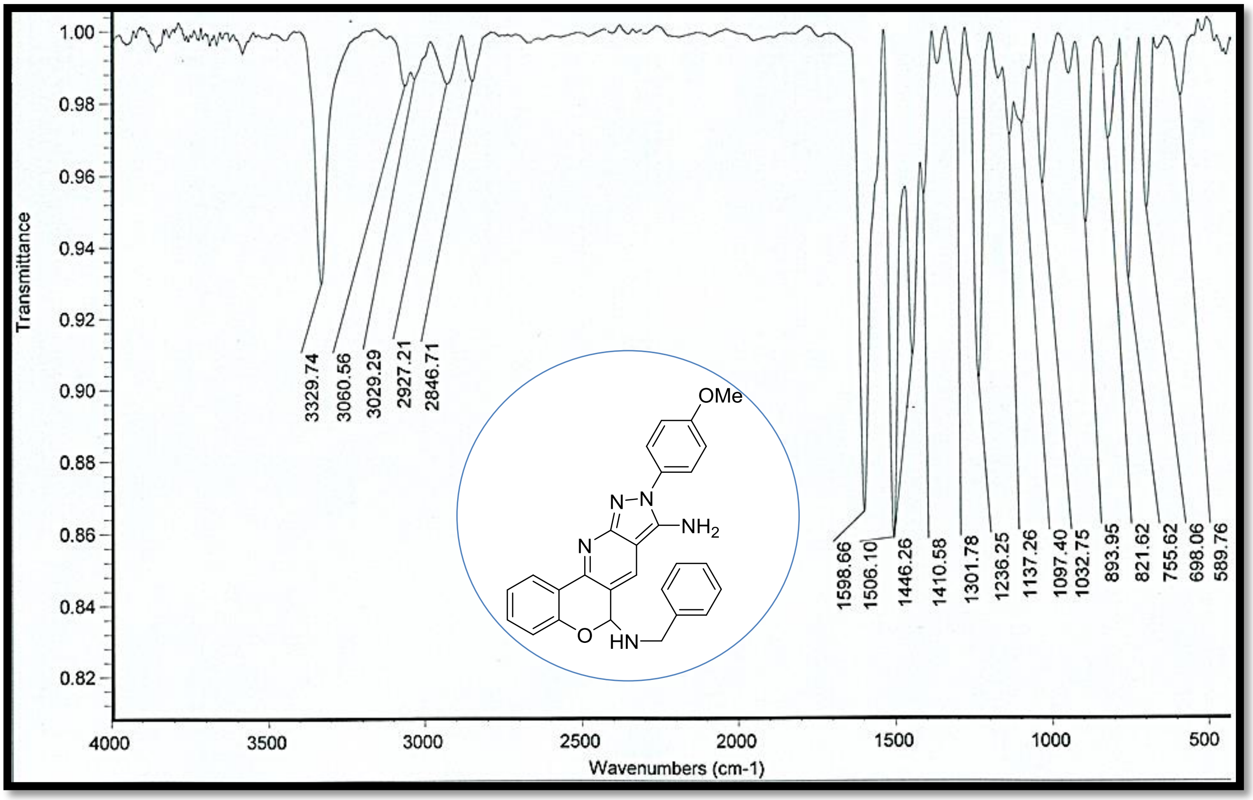

IR (KBr) (**4k**)

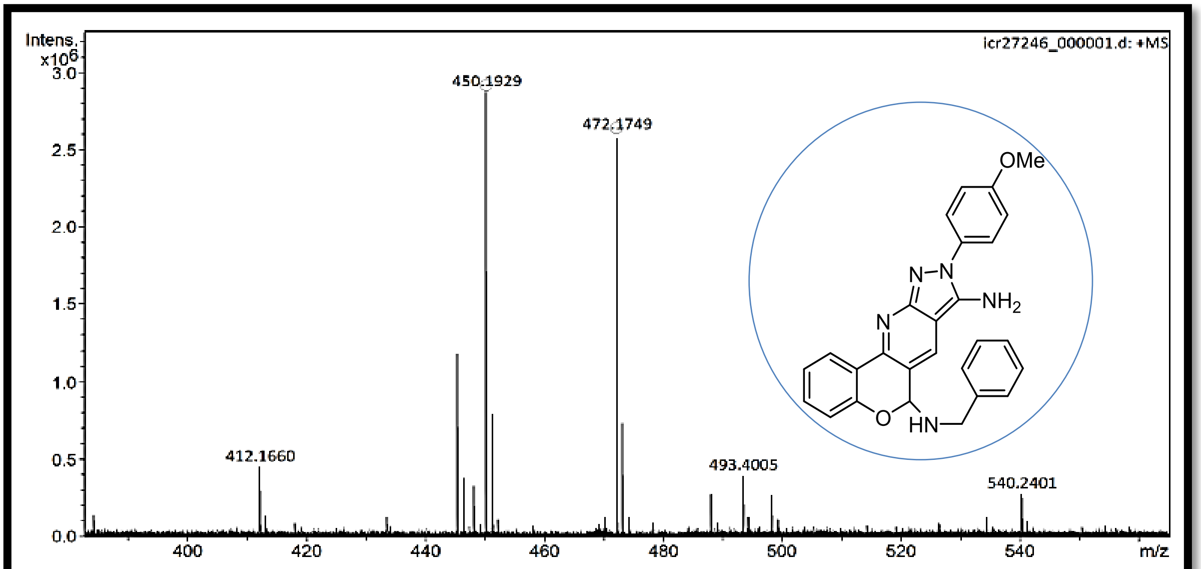

Meas. m/z	Ion Formula	m/z	err [ppm]	mSigma	rdb	e⁻ Conf	N-Rule
450.1929	C27H24N5O2	450.1925	-1.0	21.4	18.5	even	ok
	C29H26N2O3	450.1938	2.0	27.4	18.0	odd	ok
472.1749	C26H24N4O5	472.1741	-1.6	19.0	17.0	odd	ok
	C27H23N5NaO2	472.1744	-1.1	20.8	18.5	even	ok
	C28H26NO6	472.1755	1.2	23.1	16.5	even	ok
	C27H20N8O	472.1755	1.2	23.3	22.0	odd	ok
	C25H18N11	472.1741	-1.7	25.0	22.5	even	ok
	C29H25N2NaO3	472.1757	1.8	26.0	18.0	odd	ok

HR-Mass (ESI) (**4k**)

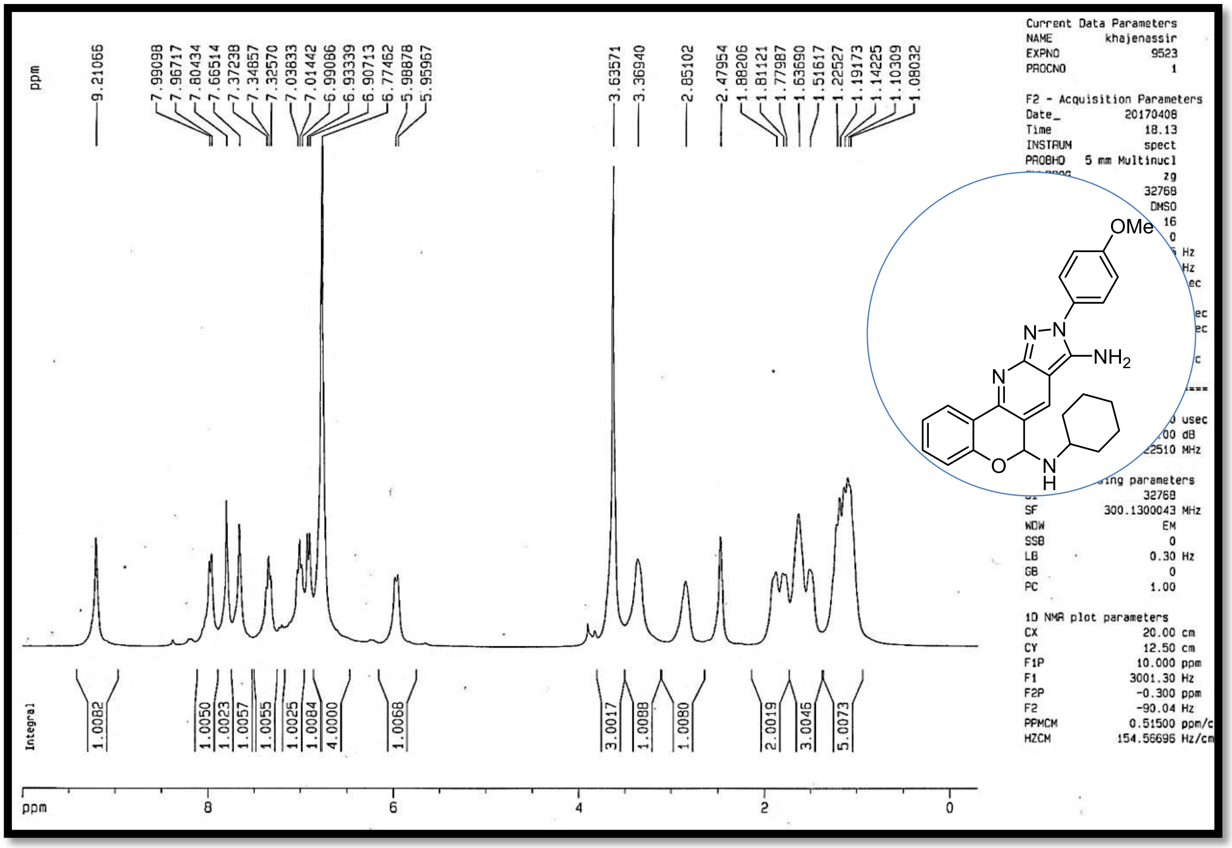

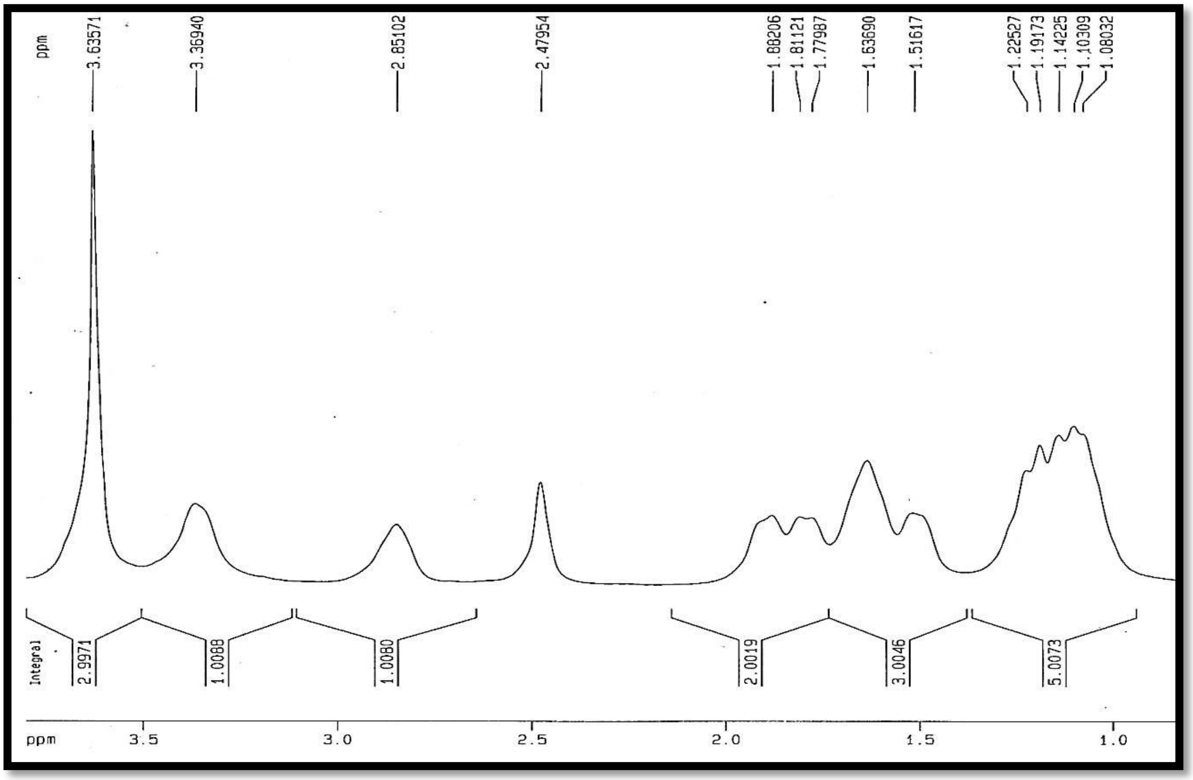

^{1}H-NMR (300 MHz, DMSO- d_6) (**4l**)

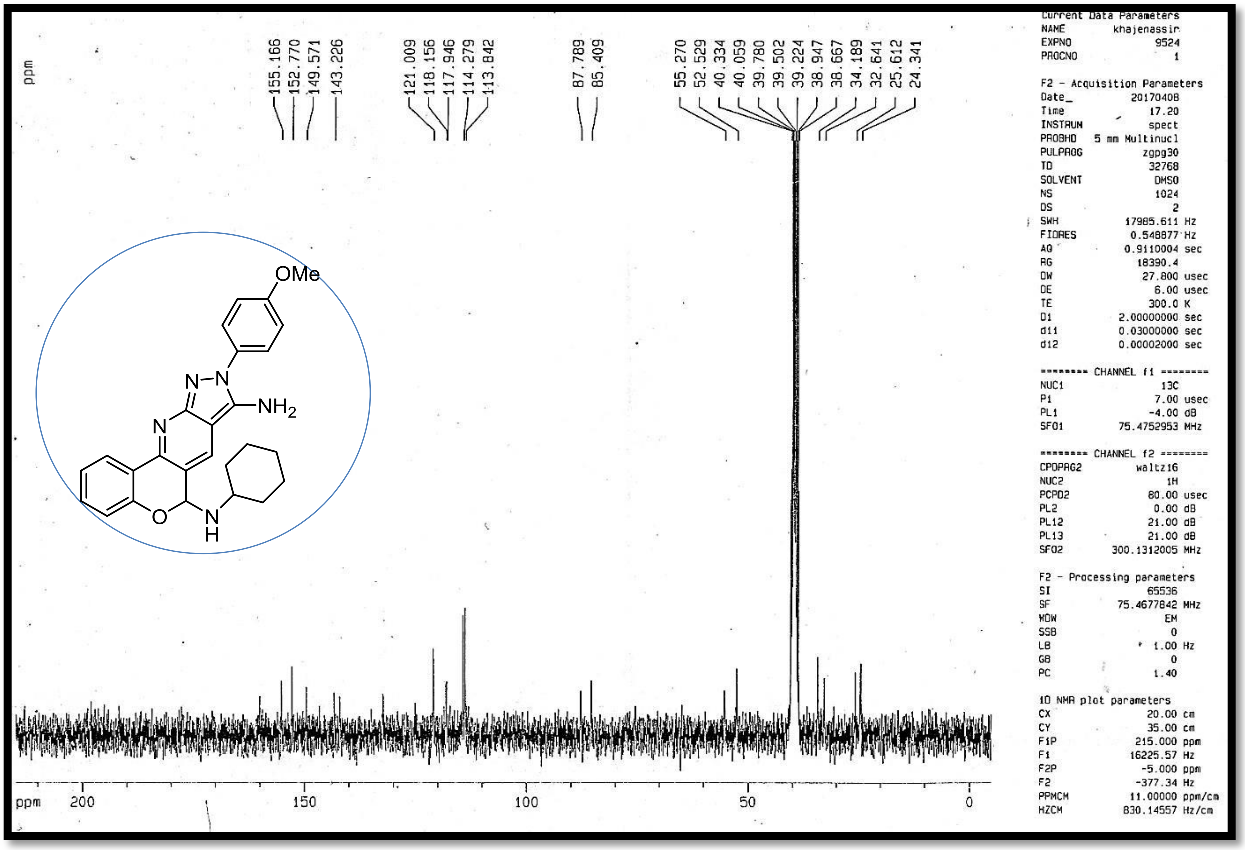

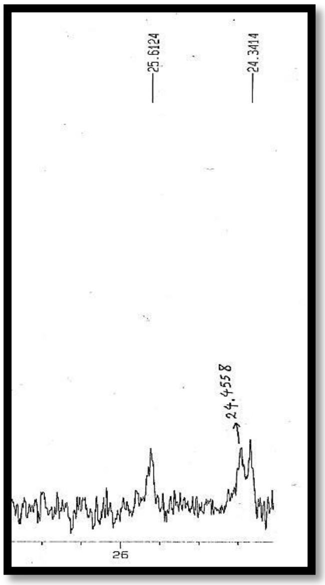

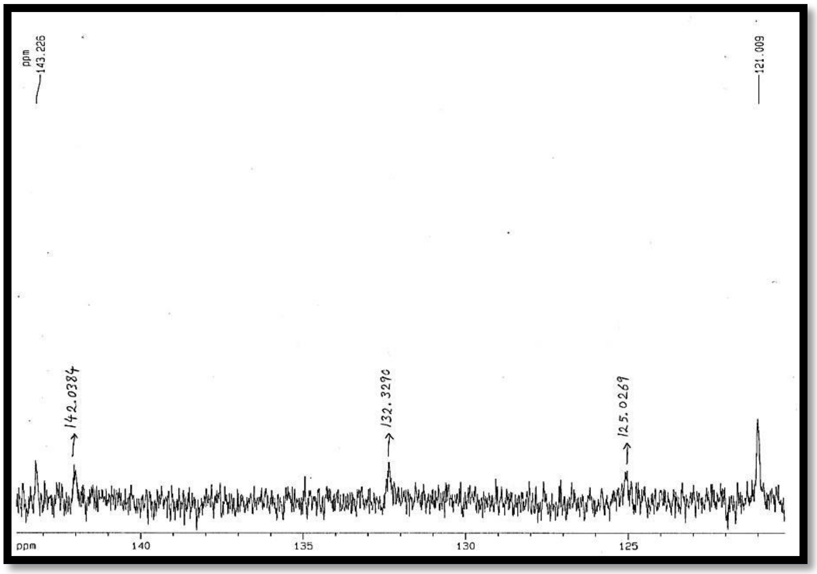

^{13}C-NMR（75 MHz, DMSO-d_6） (**4m**)

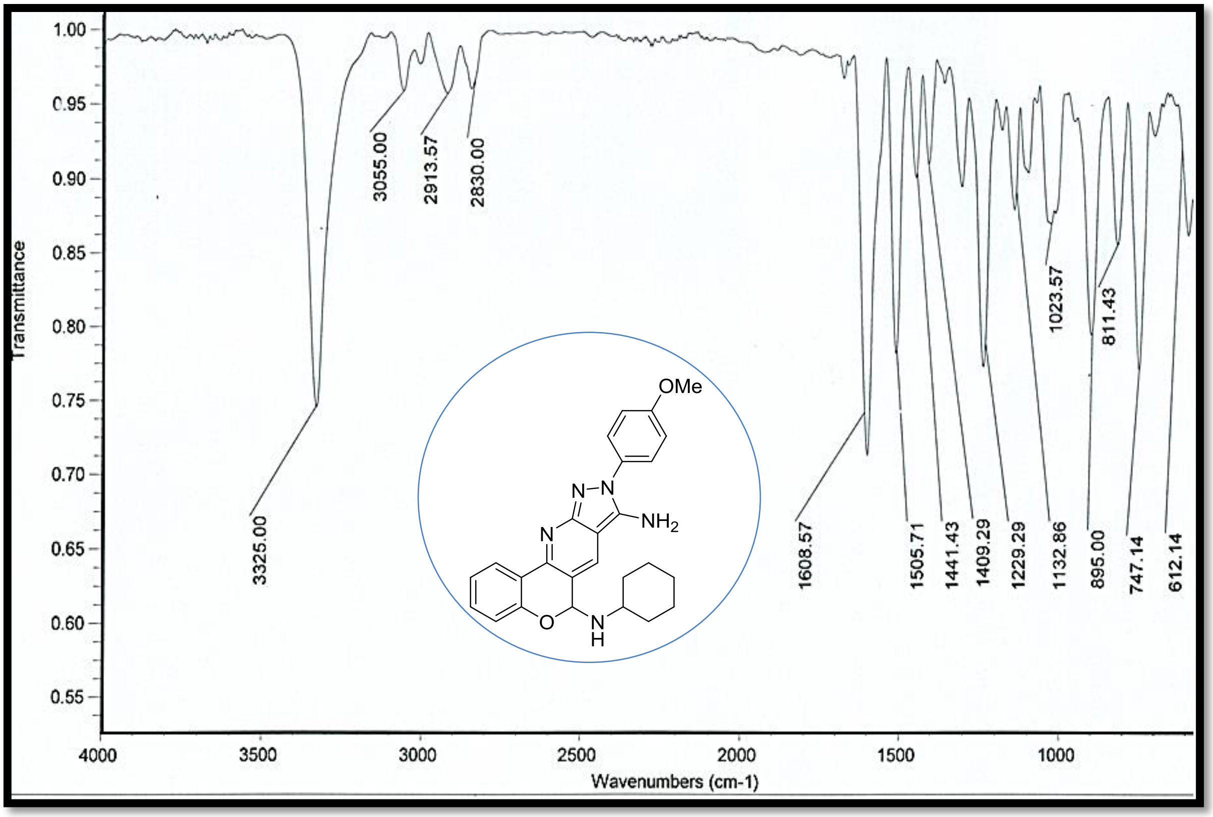

IR (KBr) (**4l**)

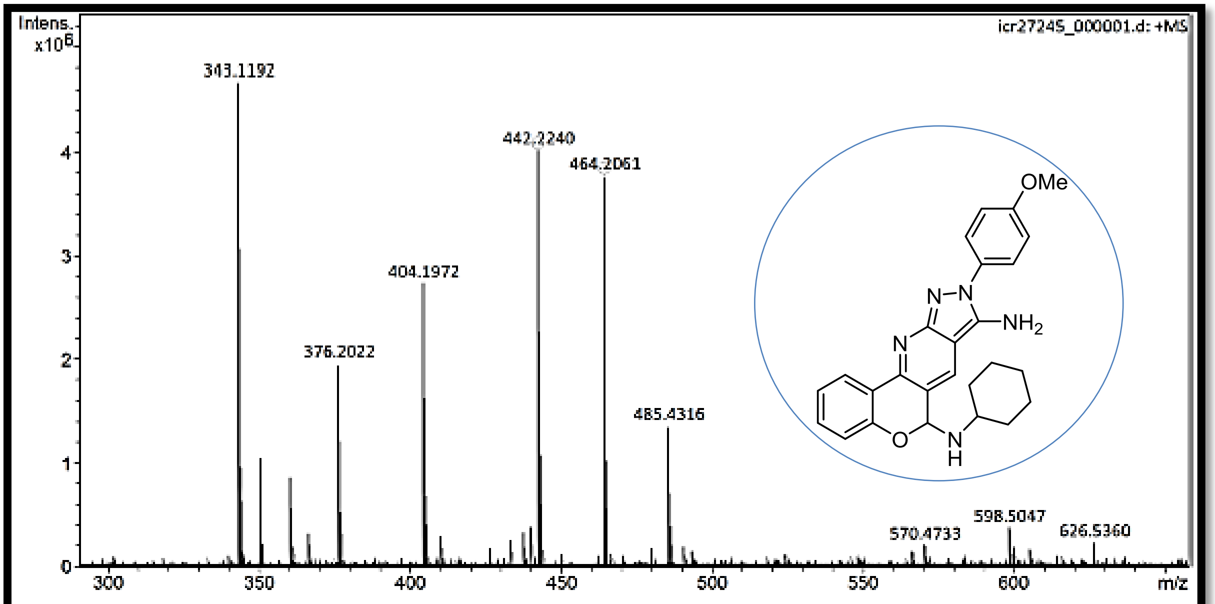

Meas. m/z	Ion Formula	m/z	err [ppm]	mSigma	rdb	e⁻ Conf	N-Rule
442.2240	C26H28N5O2	442.2238	-0.6	19.7	15.5	even	ok
464.2061	C25H28N4O5	464.2054	-1.4	17.8	14.0	odd	ok
	C26H27N5NaO2	464.2057	-0.8	19.5	15.5	even	ok
	C27H30NO6	464.2068	1.5	21.8	13.5	even	ok
	C26H24N8O	464.2068	1.5	22.1	19.0	odd	ok
	C24H22N11	464.2054	-1.4	24.3	19.5	even	ok

HR-Mass (ESI) (**4l**)

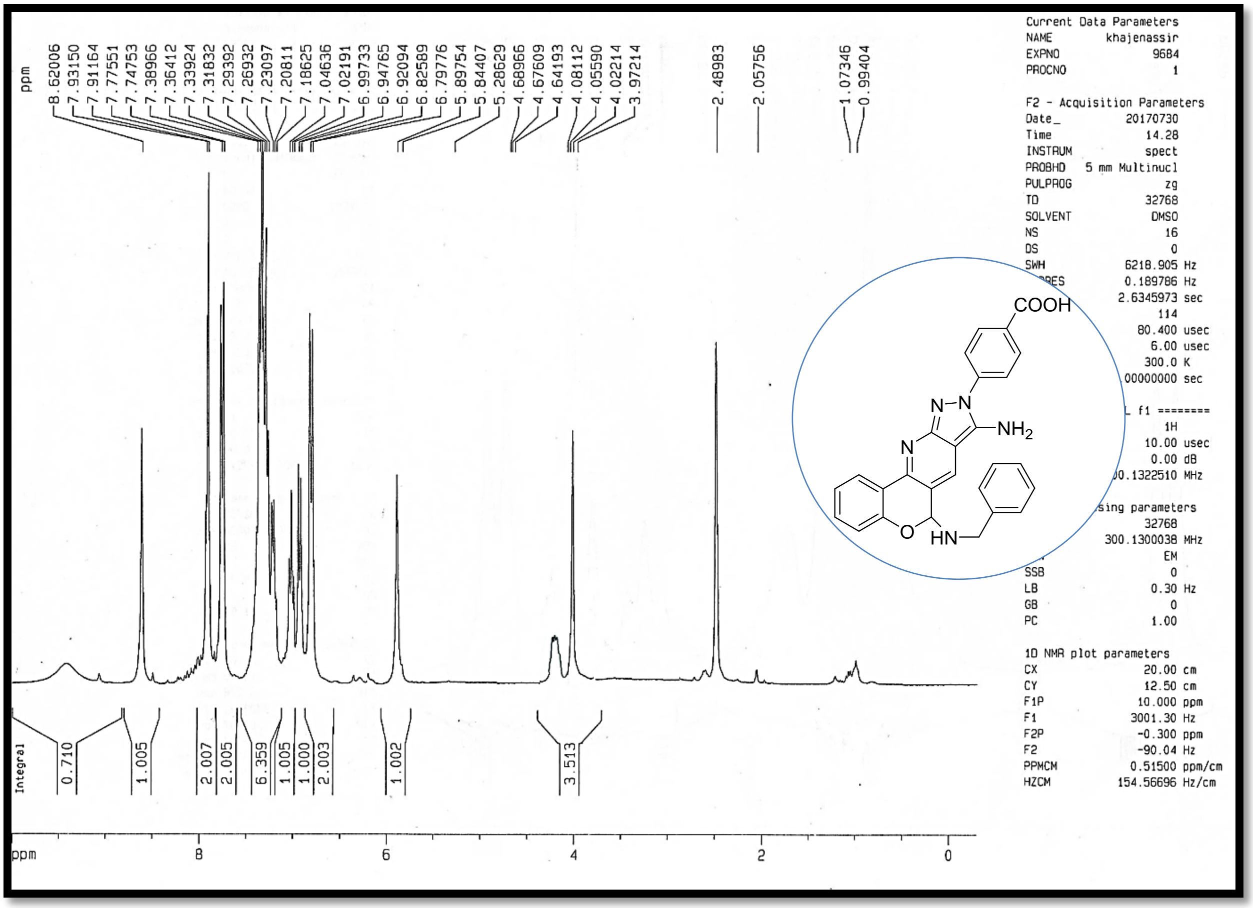

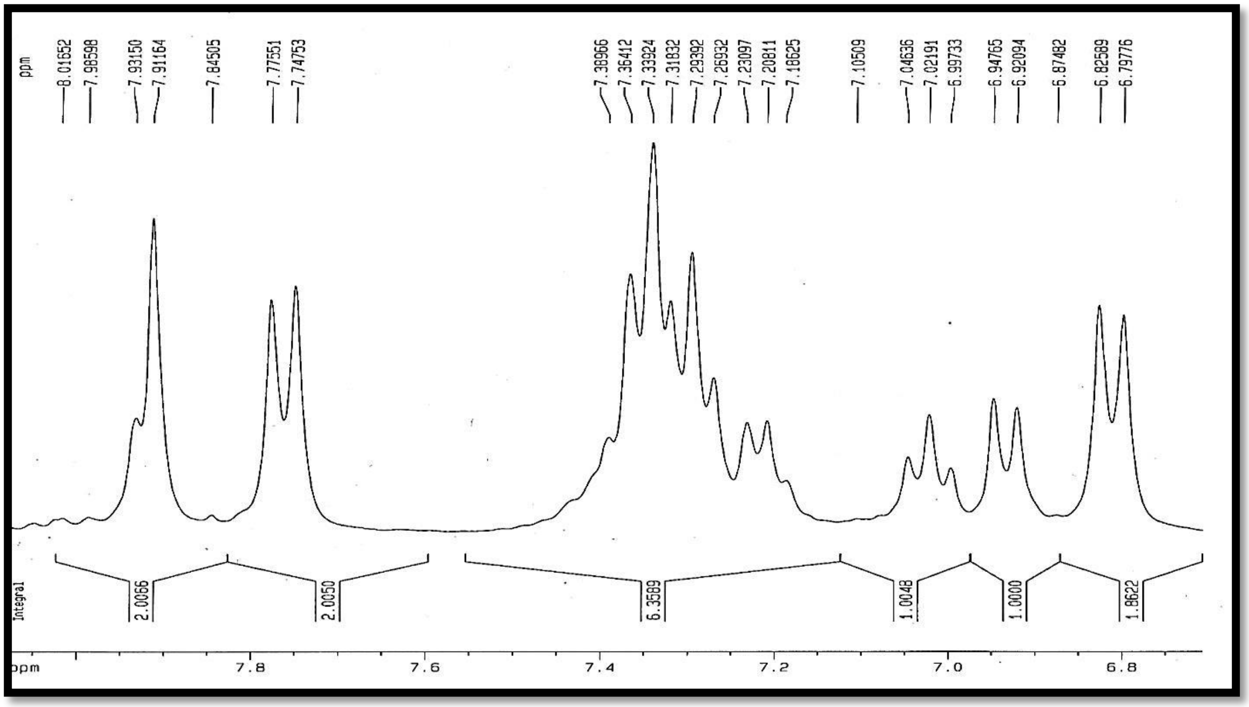

^{1}H-NMR （300 MHz, DMSO- d_6） (**4m**)

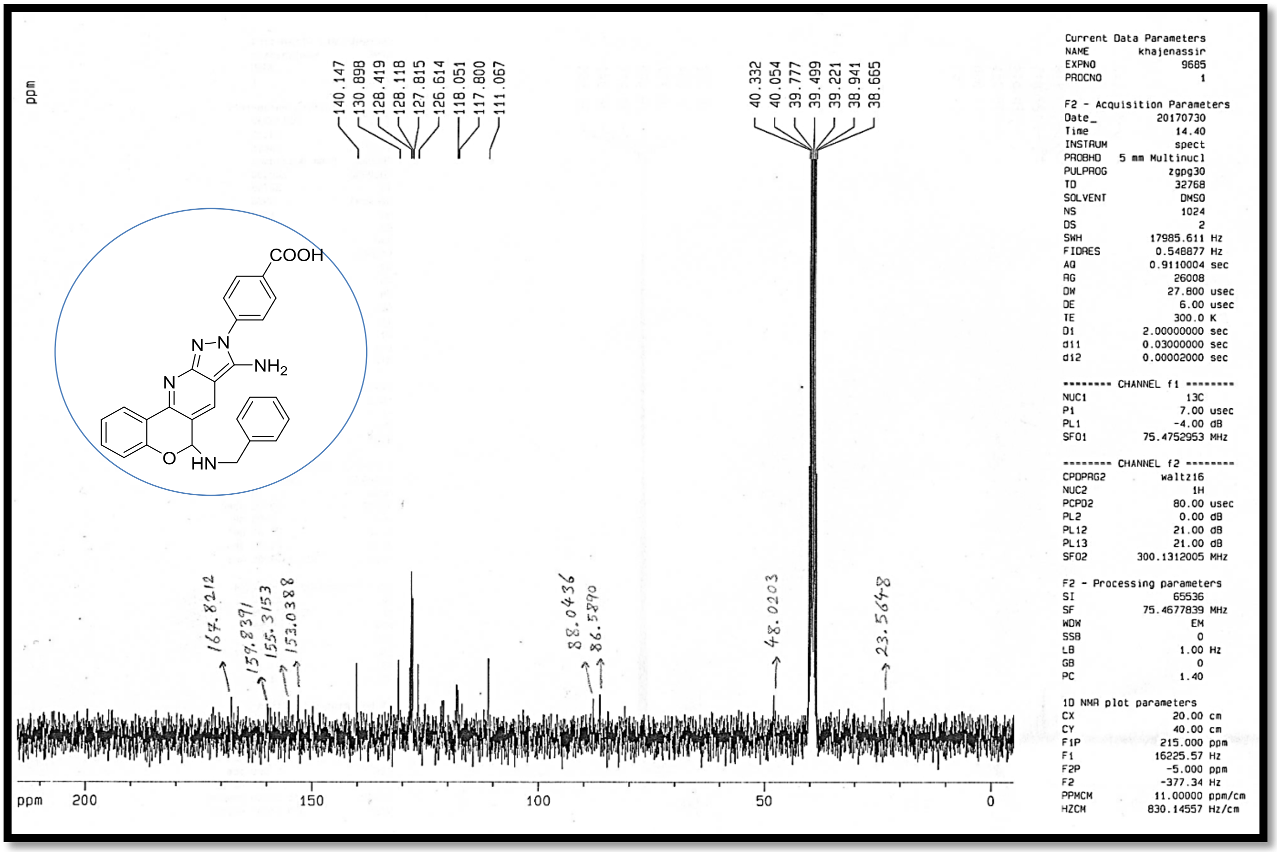

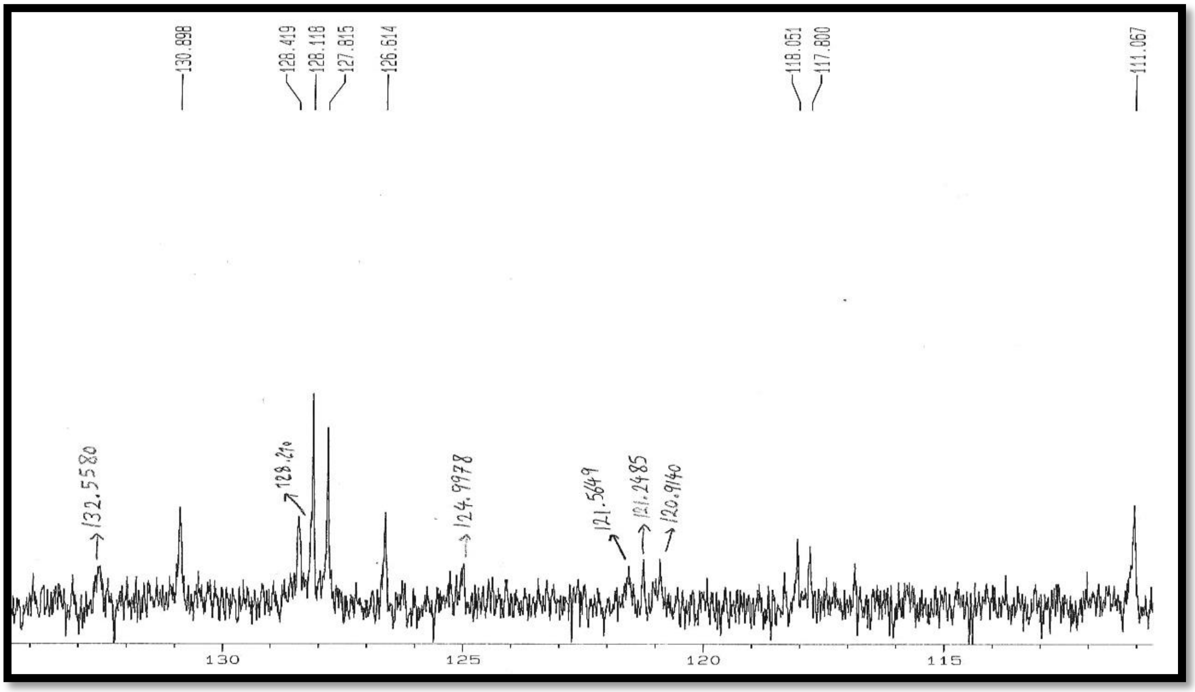

^{13}C-NMR（75 MHz, DMSO- d_6） (**4m**)

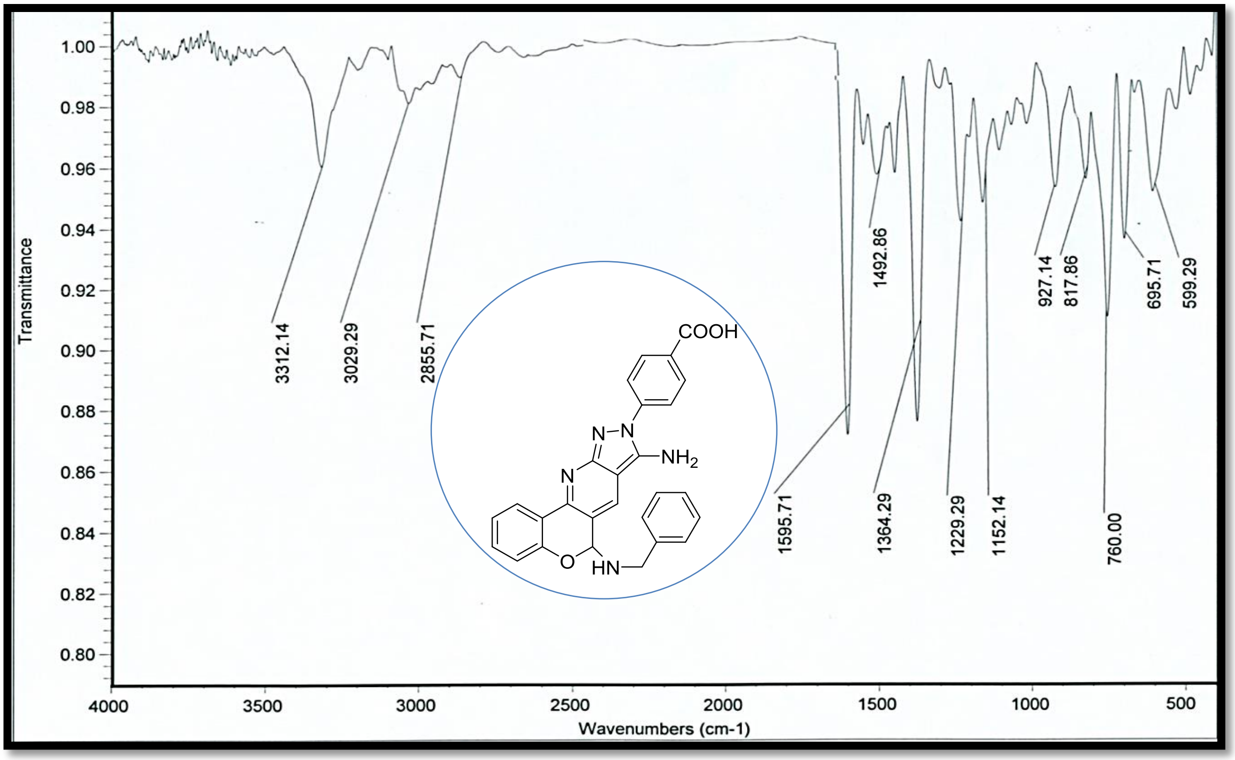

IR (KBr) (**4m**)

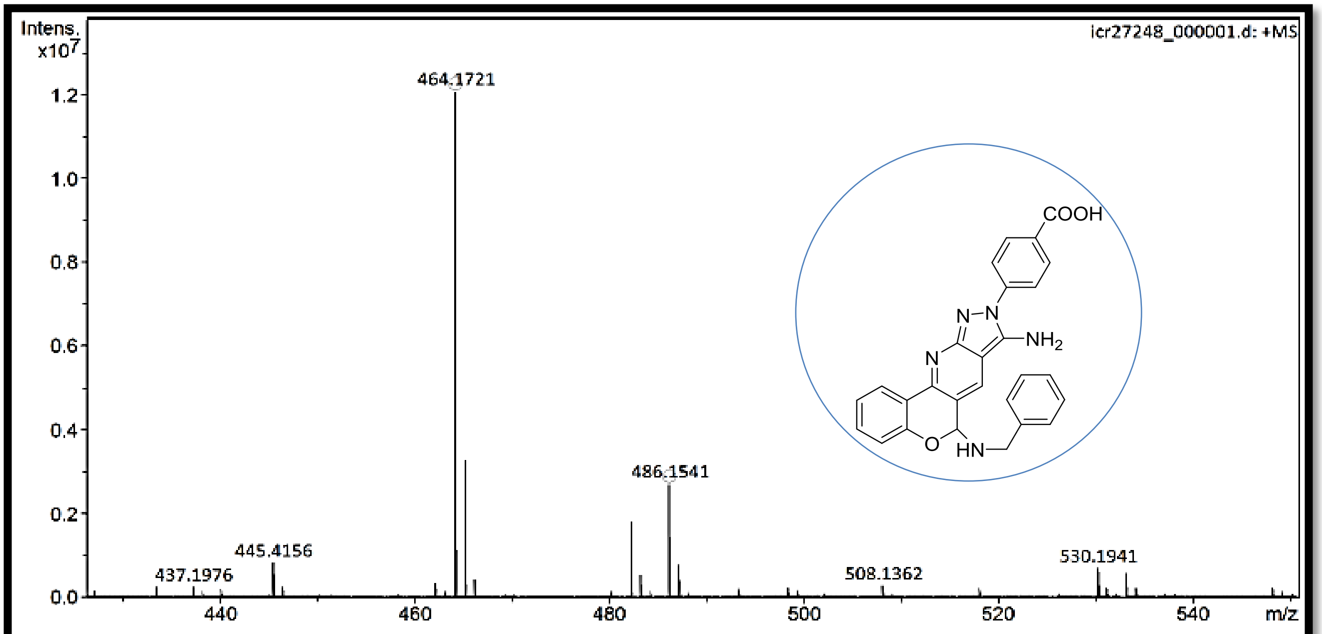

Meas. m/z	Ion Formula	m/z	err [ppm]	mSigma	rdb	e⁻ Conf	N-Rule
464.1721	C27H22N5O3	464.1717	-0.8	13.1	19.5	even	ok
486.1541	C26H22N4O6	486.1534	-1.5	19.4	18.0	odd	ok
	C27H21N5NaO3	486.1537	-1.0	20.5	19.5	even	ok
	C28H24NO7	486.1547	1.2	22.5	17.5	even	ok
	C27H18N8O2	486.1547	1.2	23.0	23.0	odd	ok
	C29H23N2NaO4	486.1550	1.8	25.0	19.0	odd	ok
	C25H16N11O	486.1534	-1.5	25.6	23.5	even	ok

HR-Mass (ESI) (**4m**)

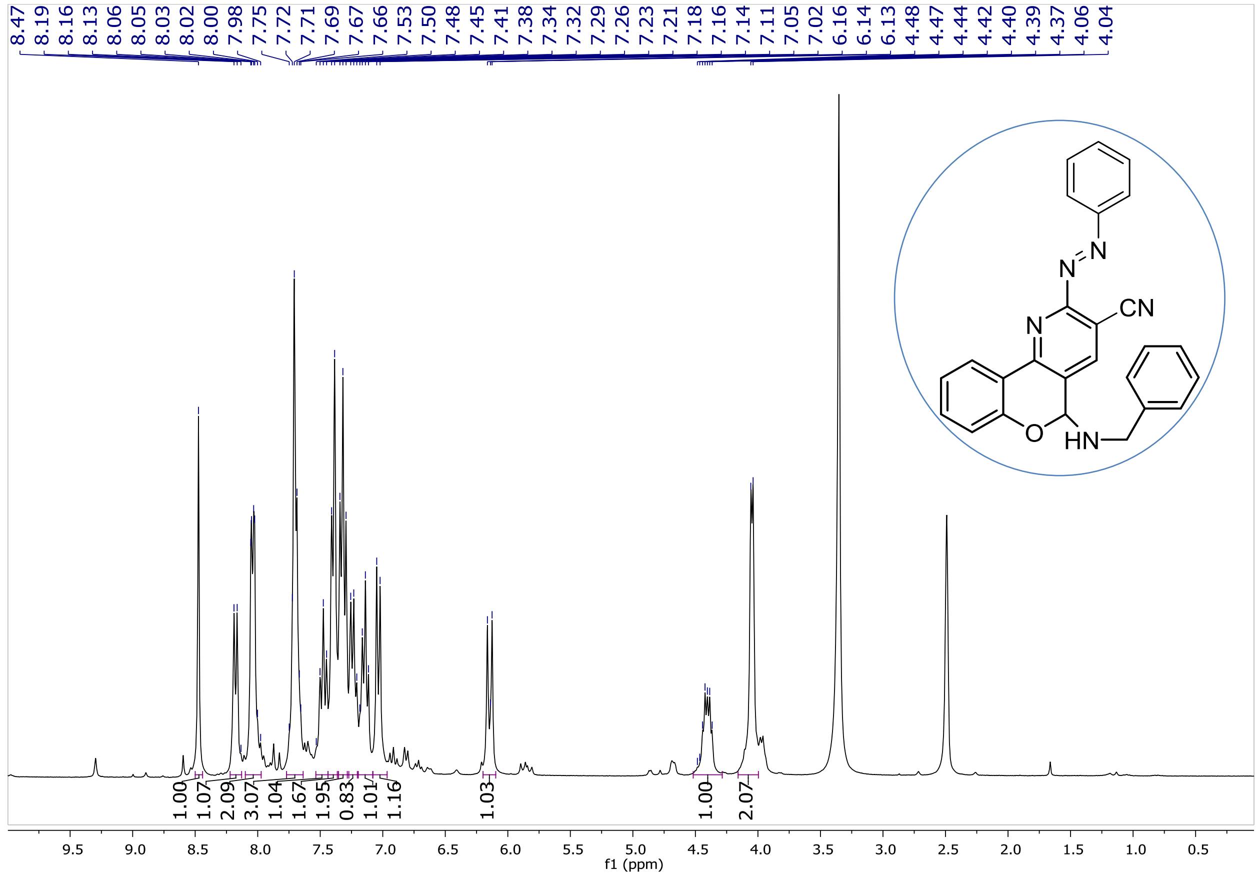

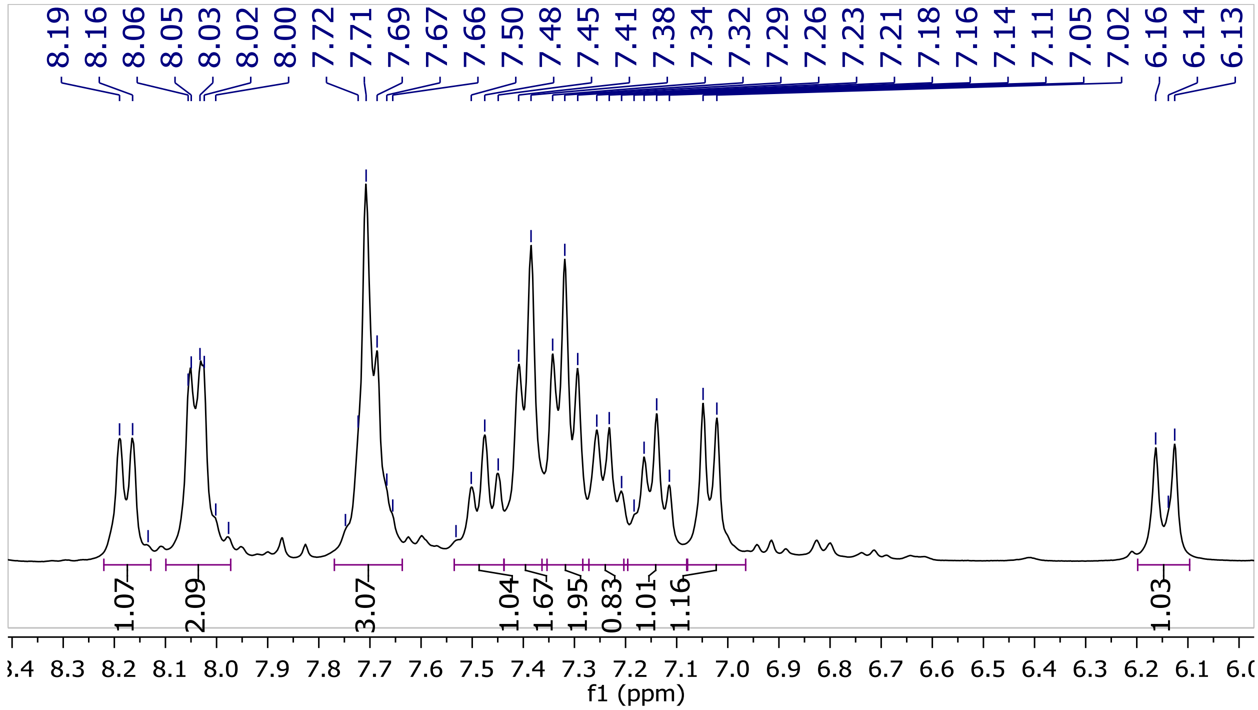

^{1}H-NMR （300 MHz, DMSO- d_6） (**5a**)

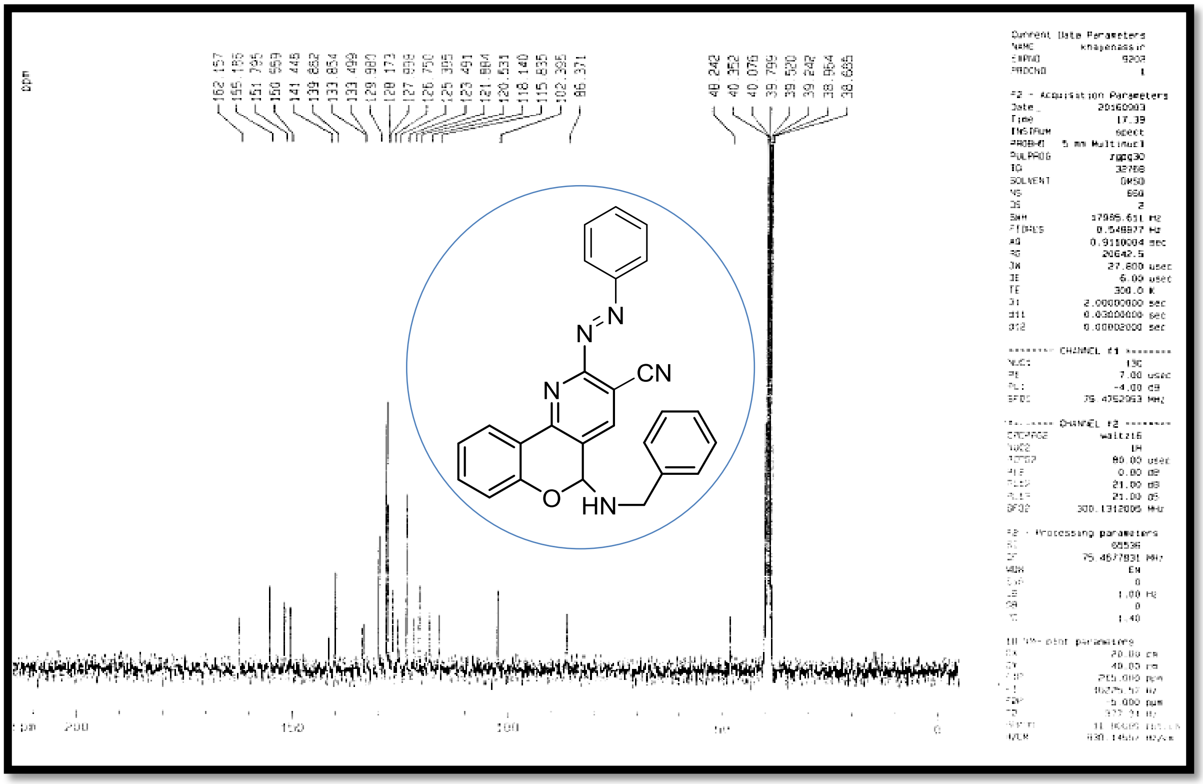
N
N
N
CN
O
HN

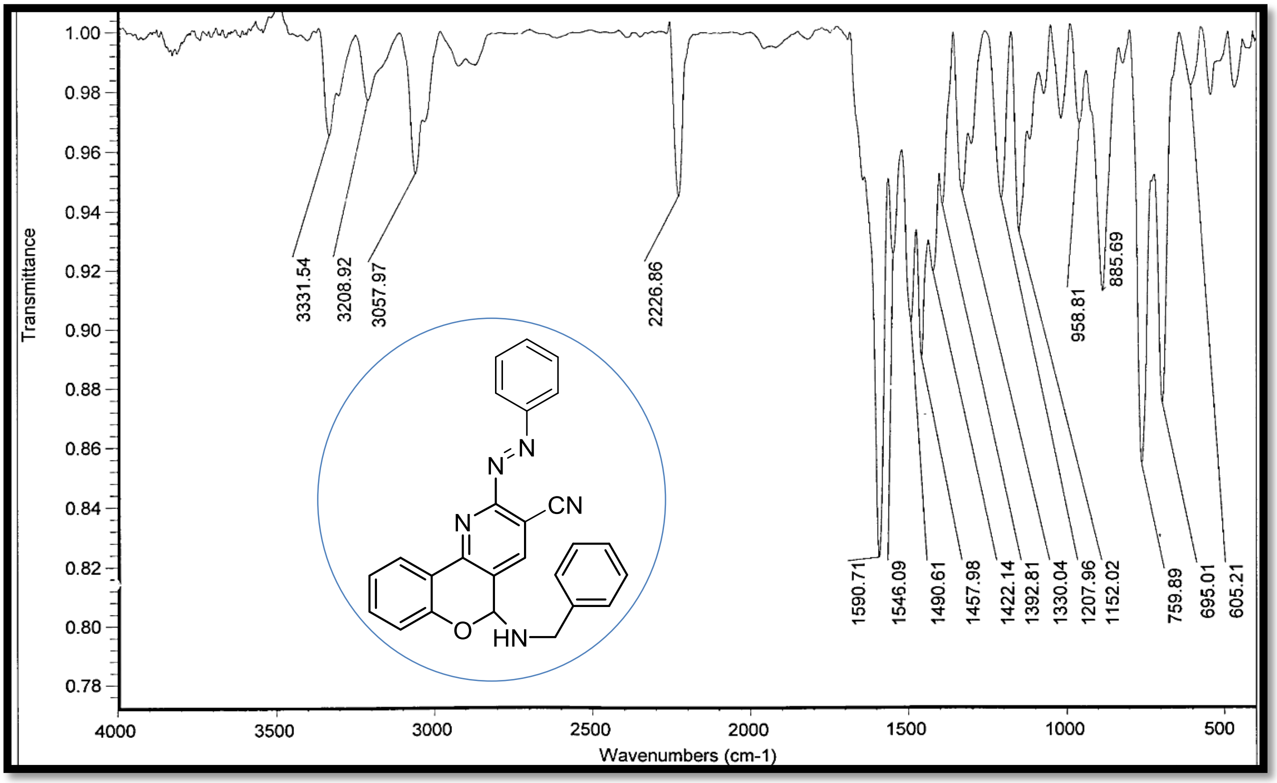
Transmittance
Wavenumbers (cm-1)
3331.54
3208.92
3057.97
2226.86
1590.71
1546.09
1490.61
1457.98
1422.14
1392.81
1330.04
1207.96
1152.02
958.81
885.69
759.89
695.01
605.21
N
N
N
CN
O
HN

IR (KBr) (**5a**)

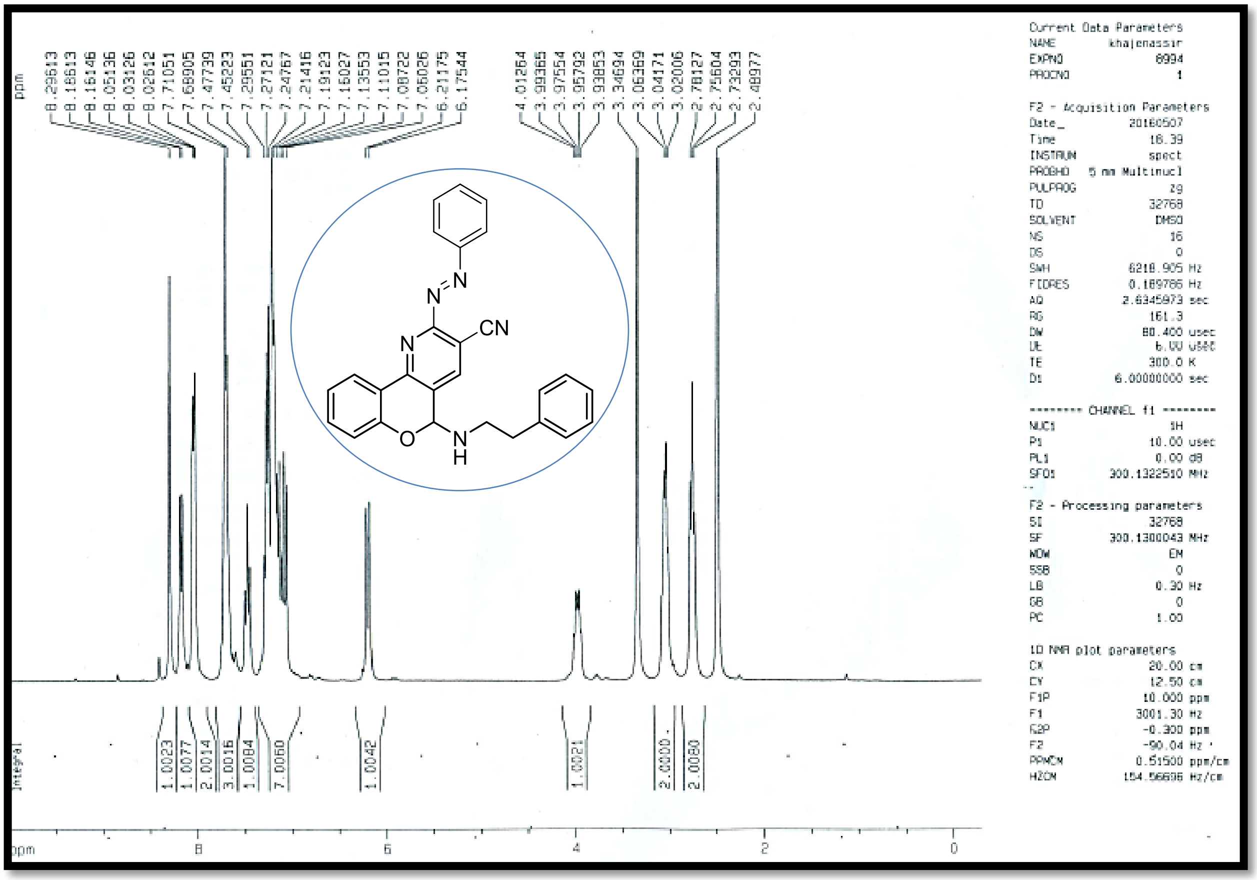

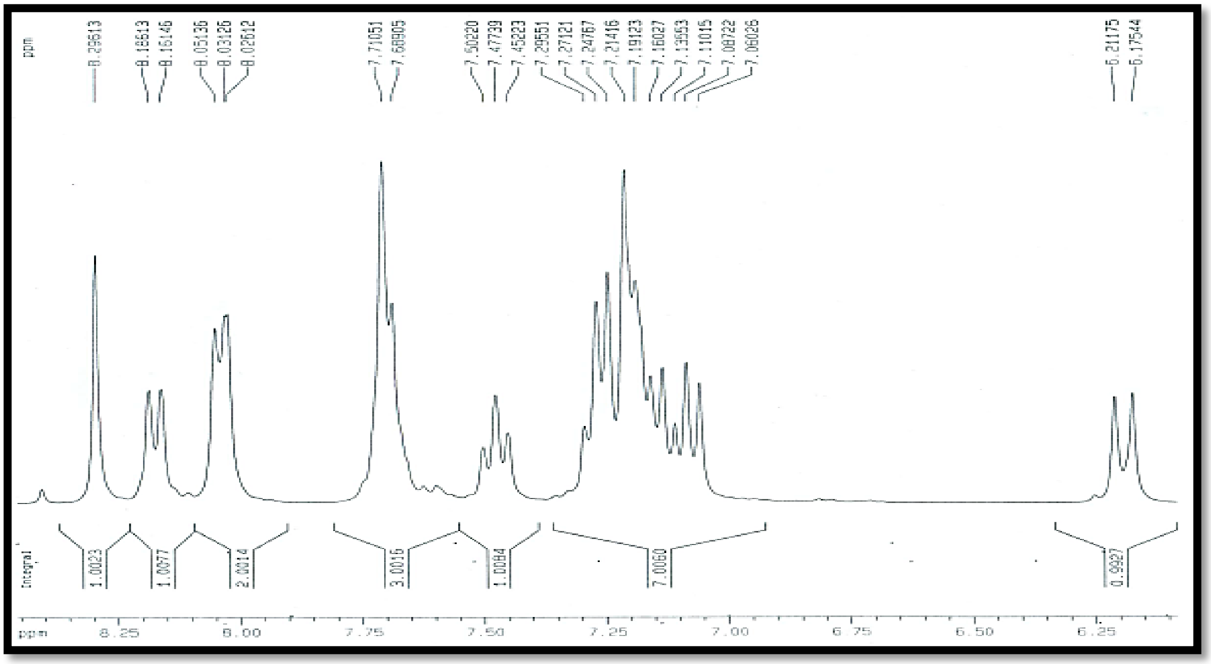

^{1}H-NMR （300 MHz, DMSO-d_6） (**5b**)

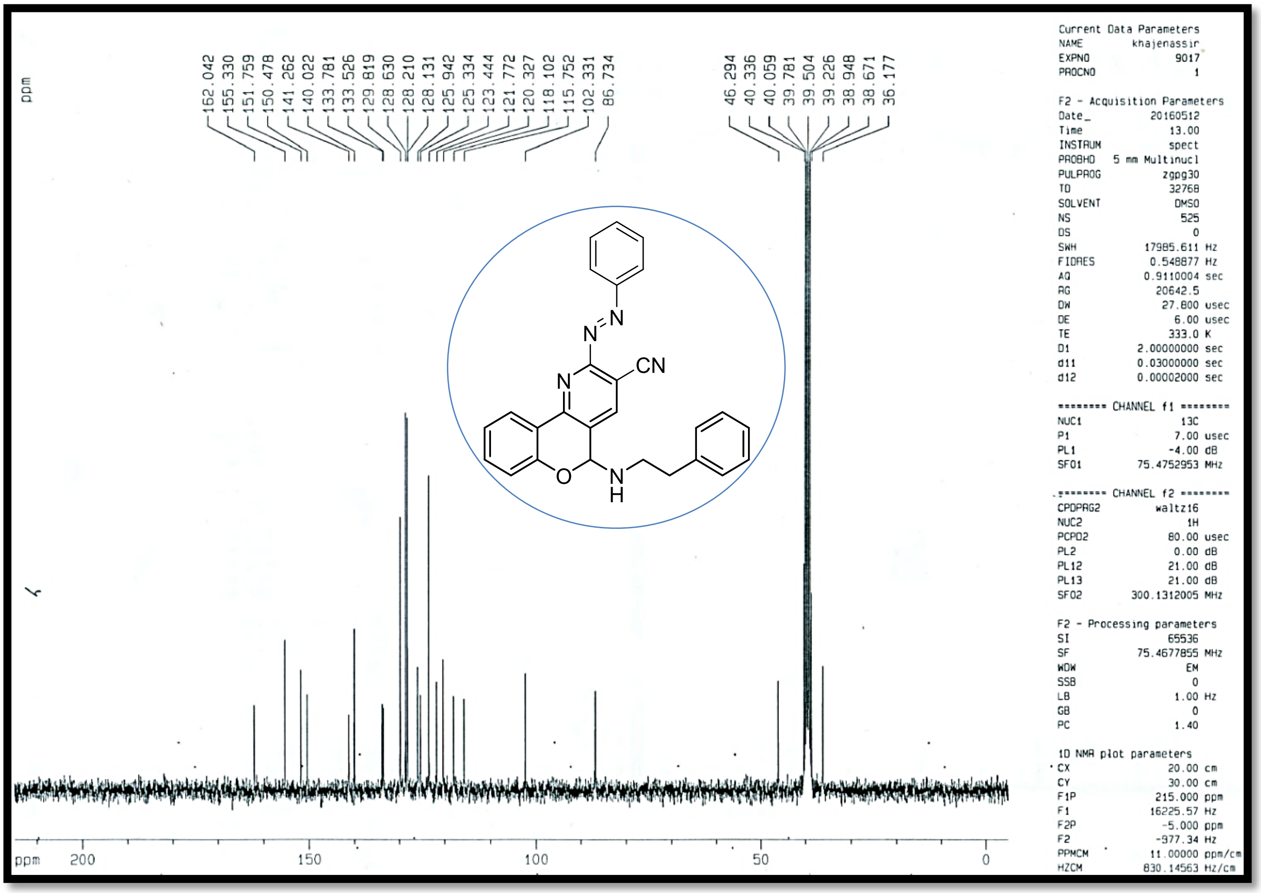

^{13}C-NMR（75 MHz, DMSO- d_6） (**5b**)

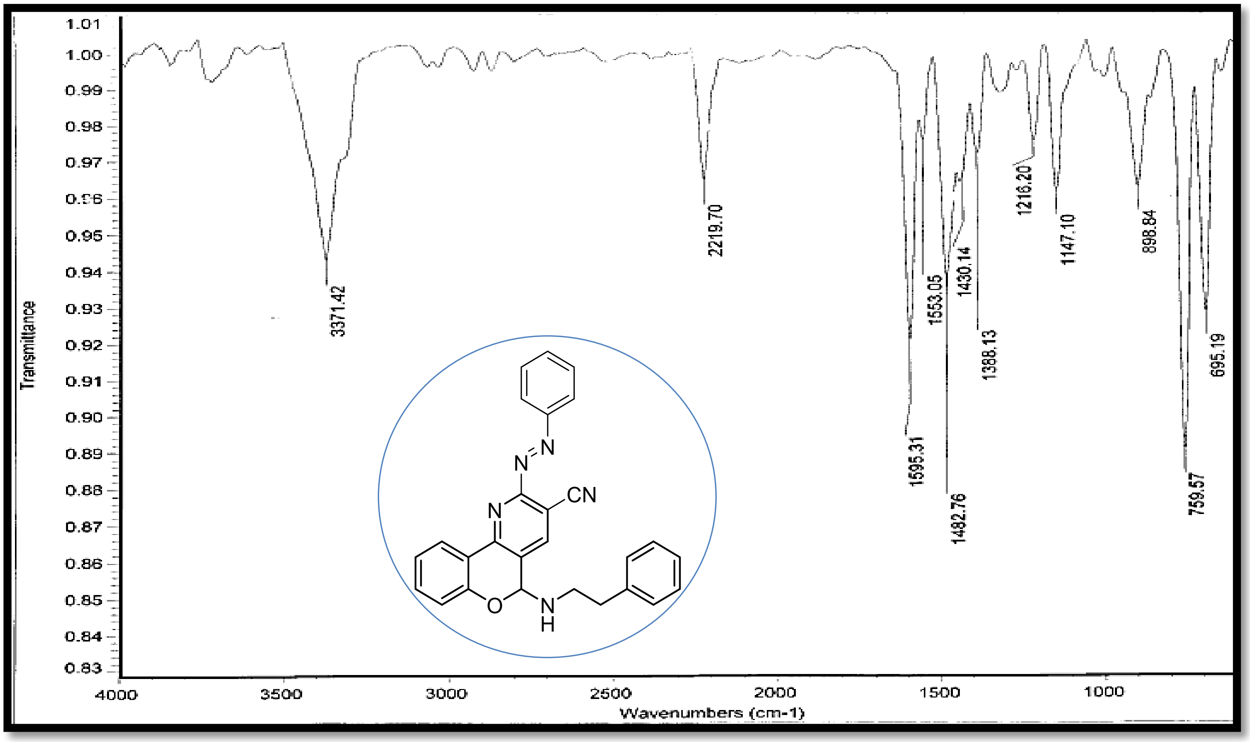

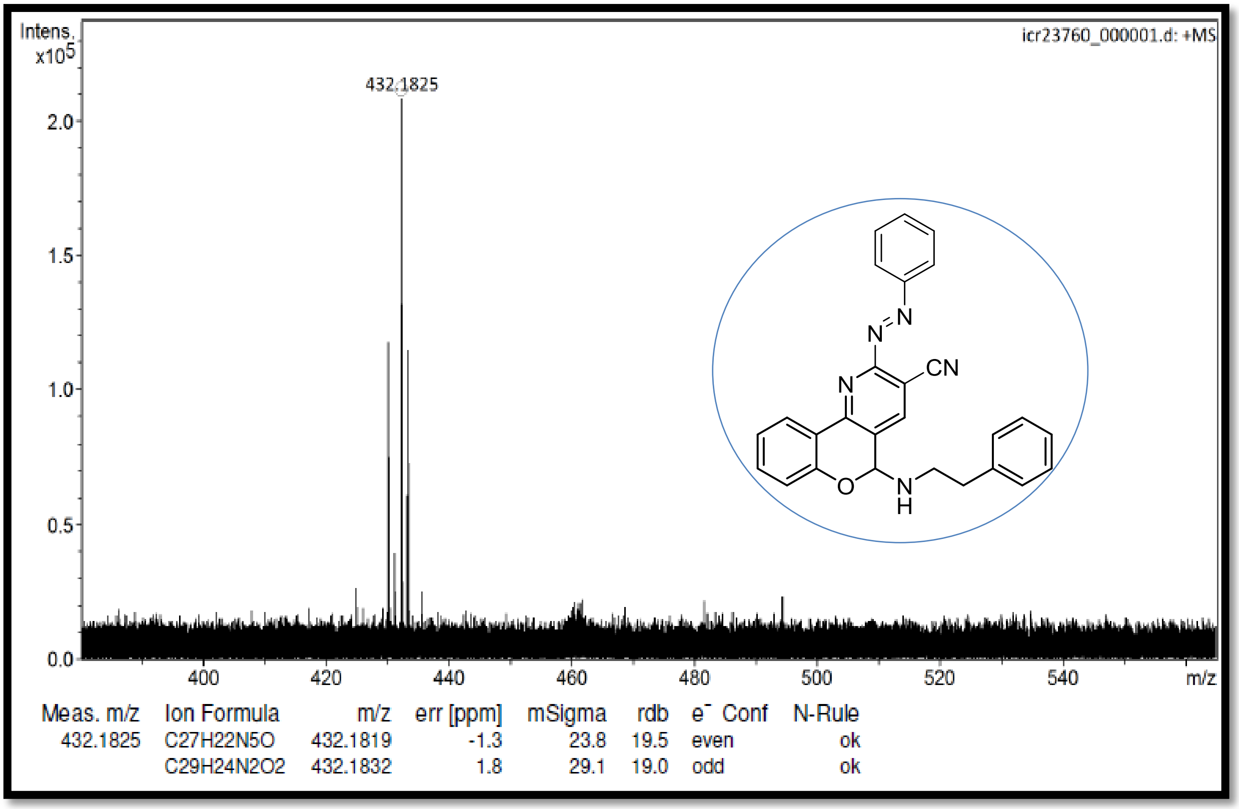

HR-Mass (ESI) (**5b**)

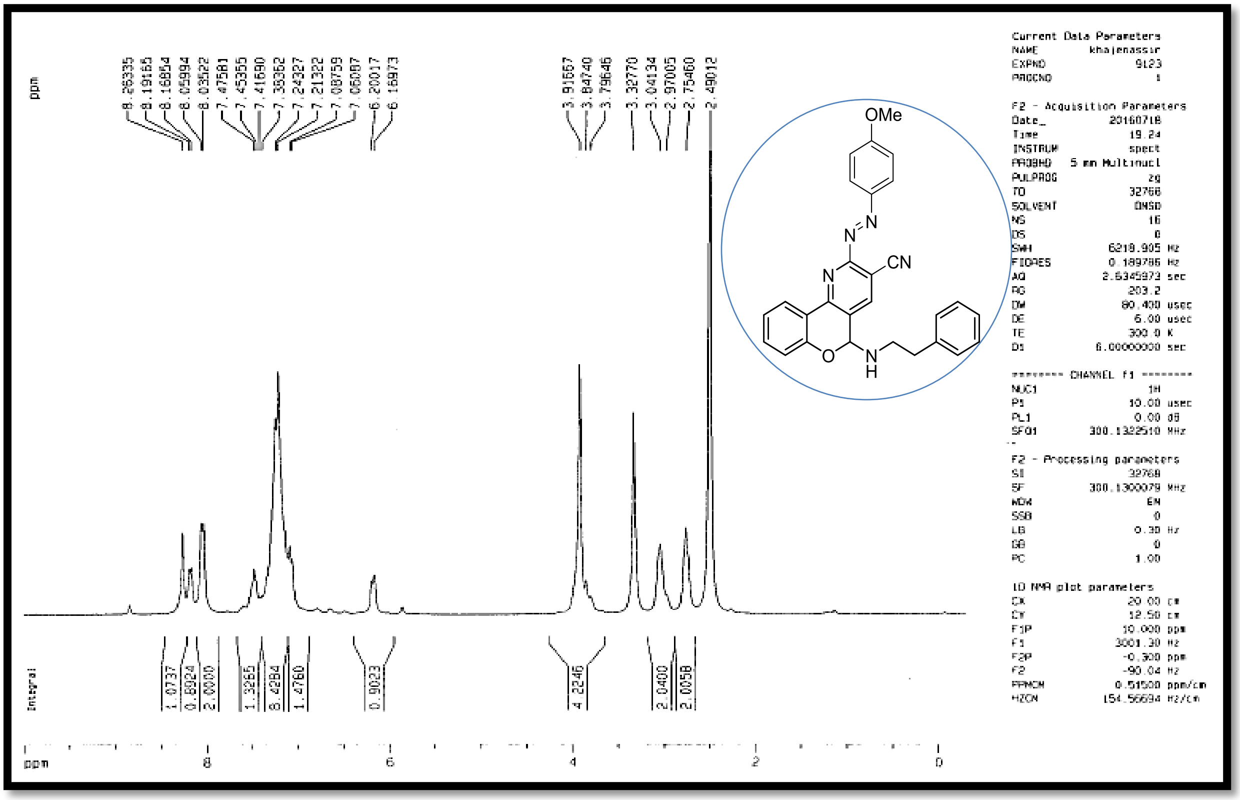

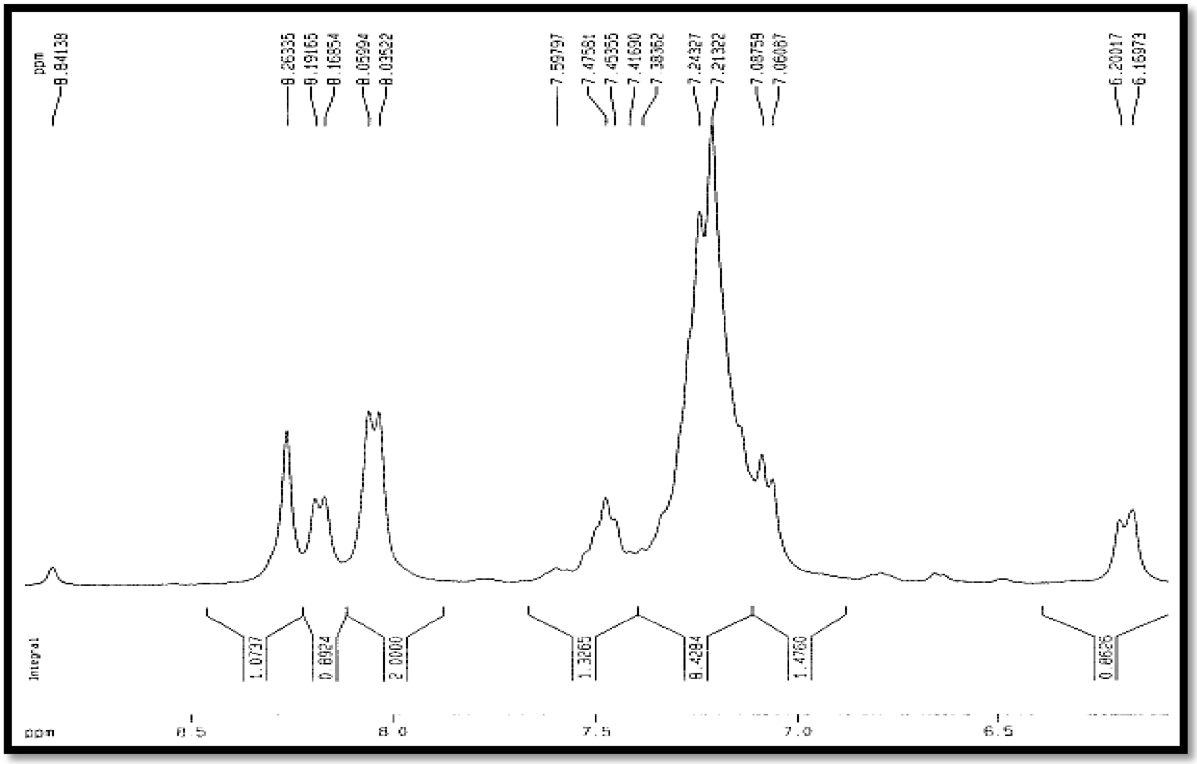

^{1}H-NMR （300 MHz, DMSO-d_6） (**5c**)

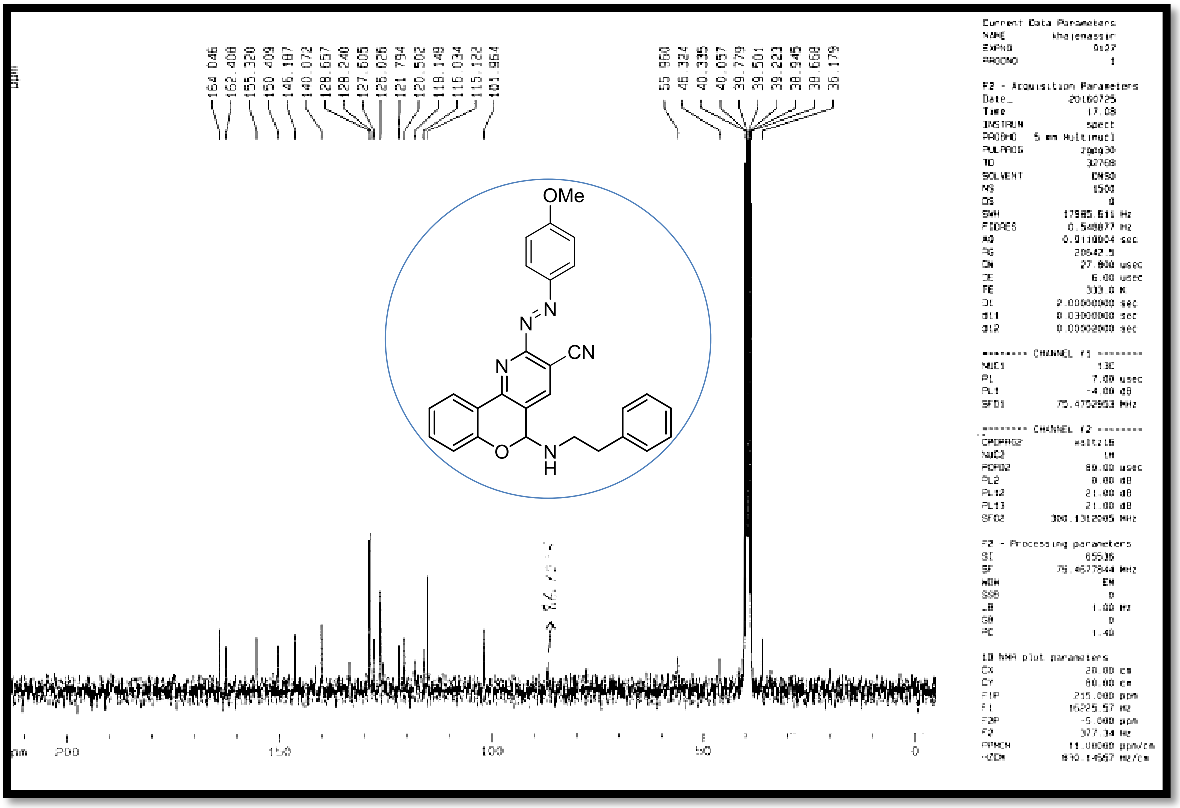

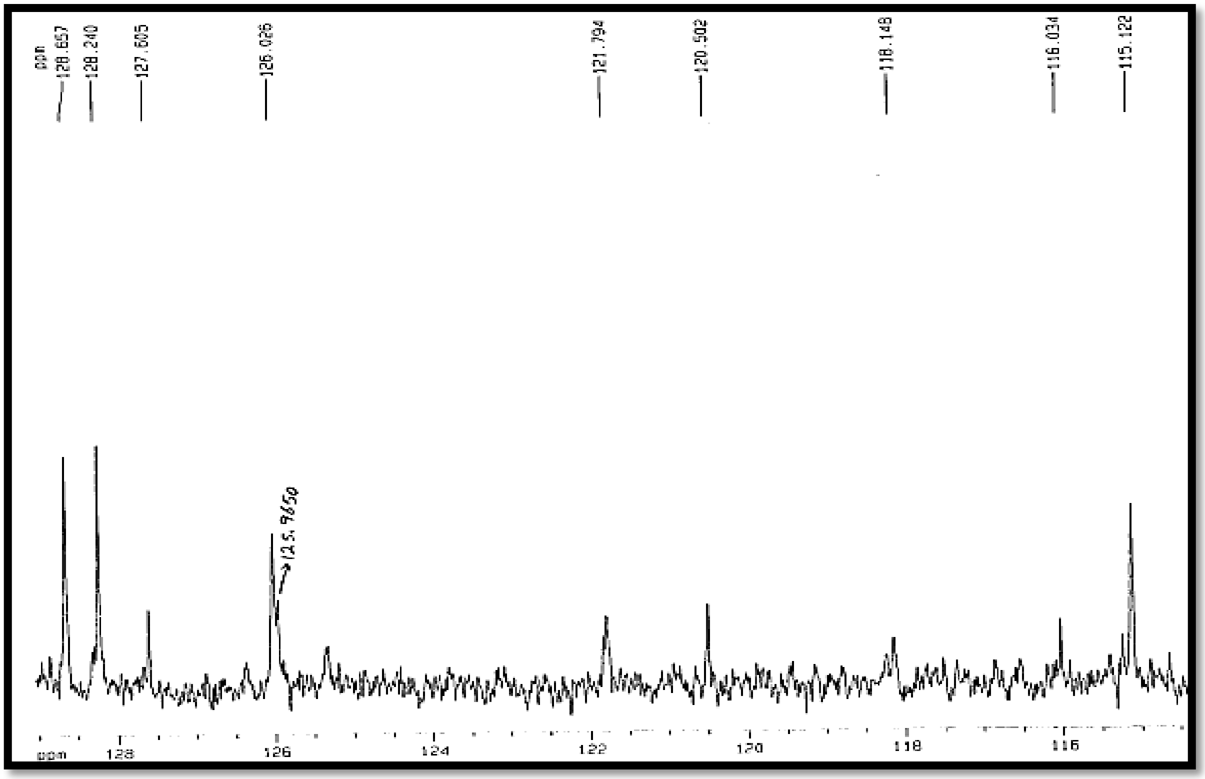

^{13}C-NMR（75 MHz, DMSO-d_6） (**5c**)

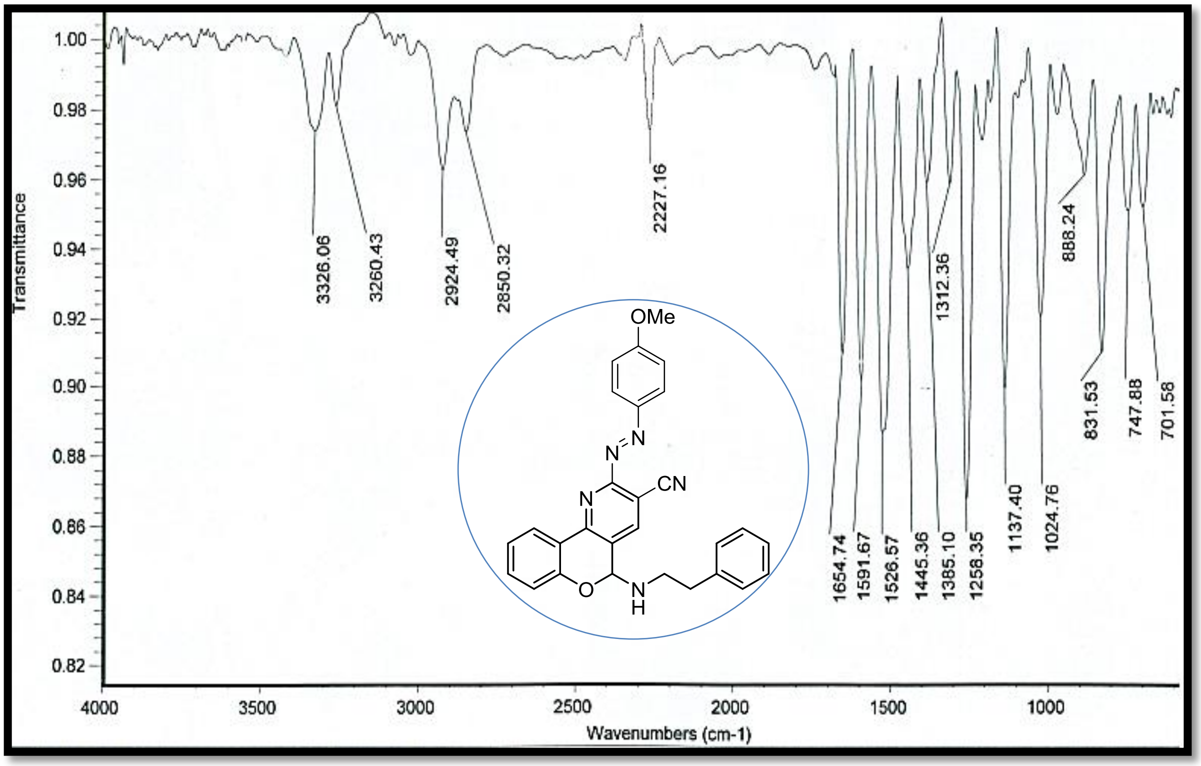

IR (KBr) (**5c**)

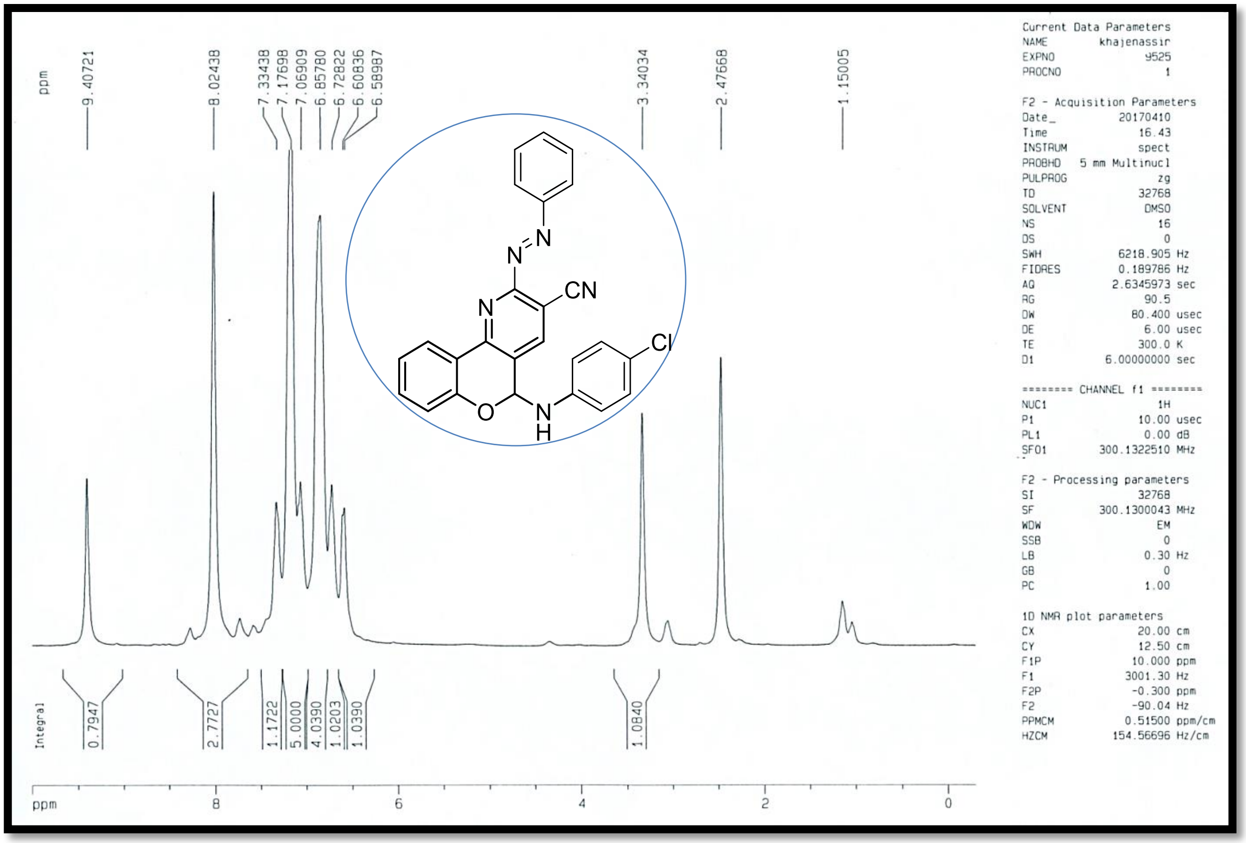

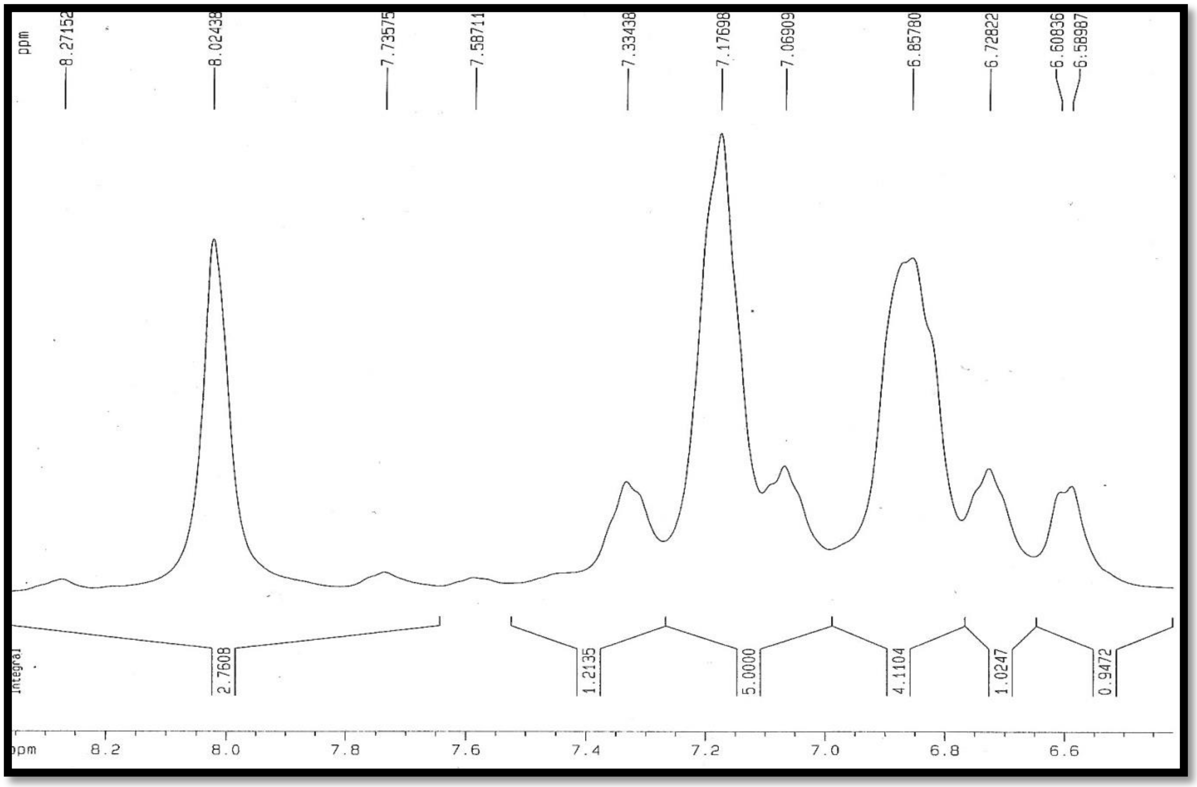

^{1}H-NMR (300 MHz, DMSO- d_6) (**5d**)

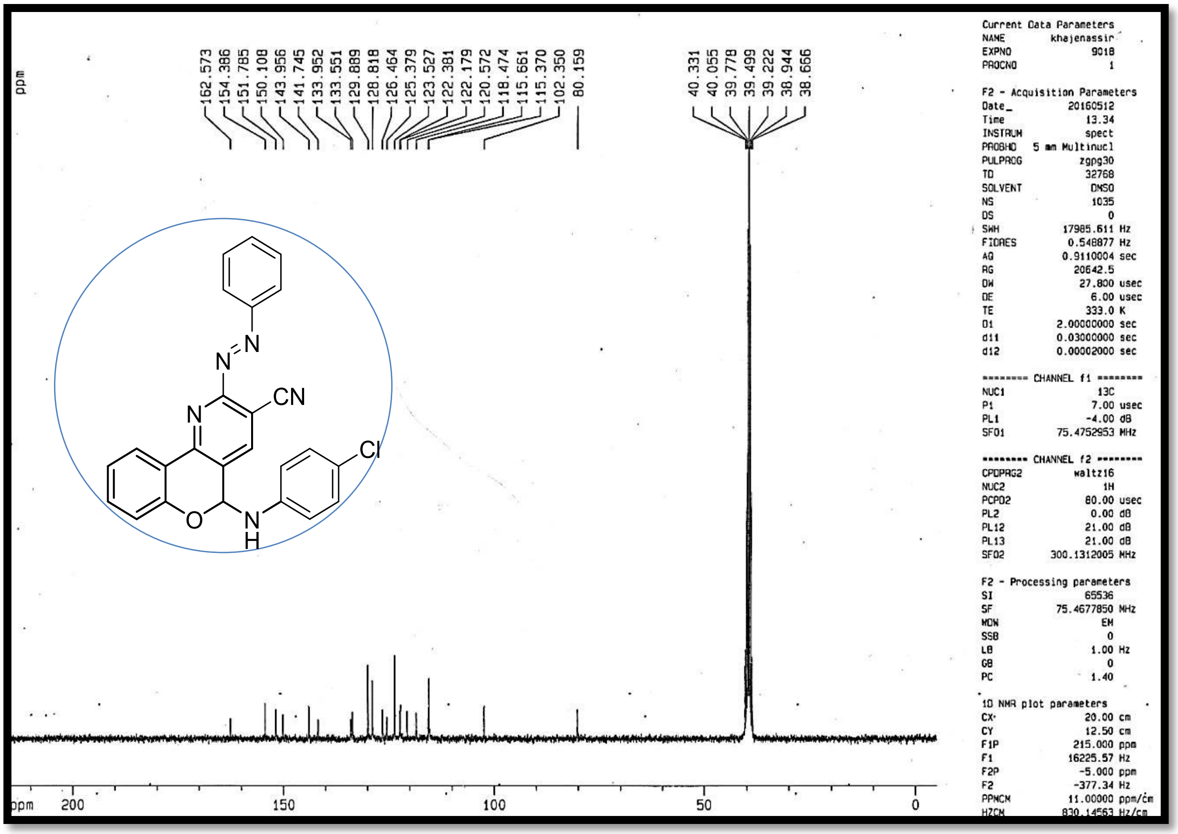

^{13}C-NMR（75 MHz, DMSO- d_6） (**5d**)

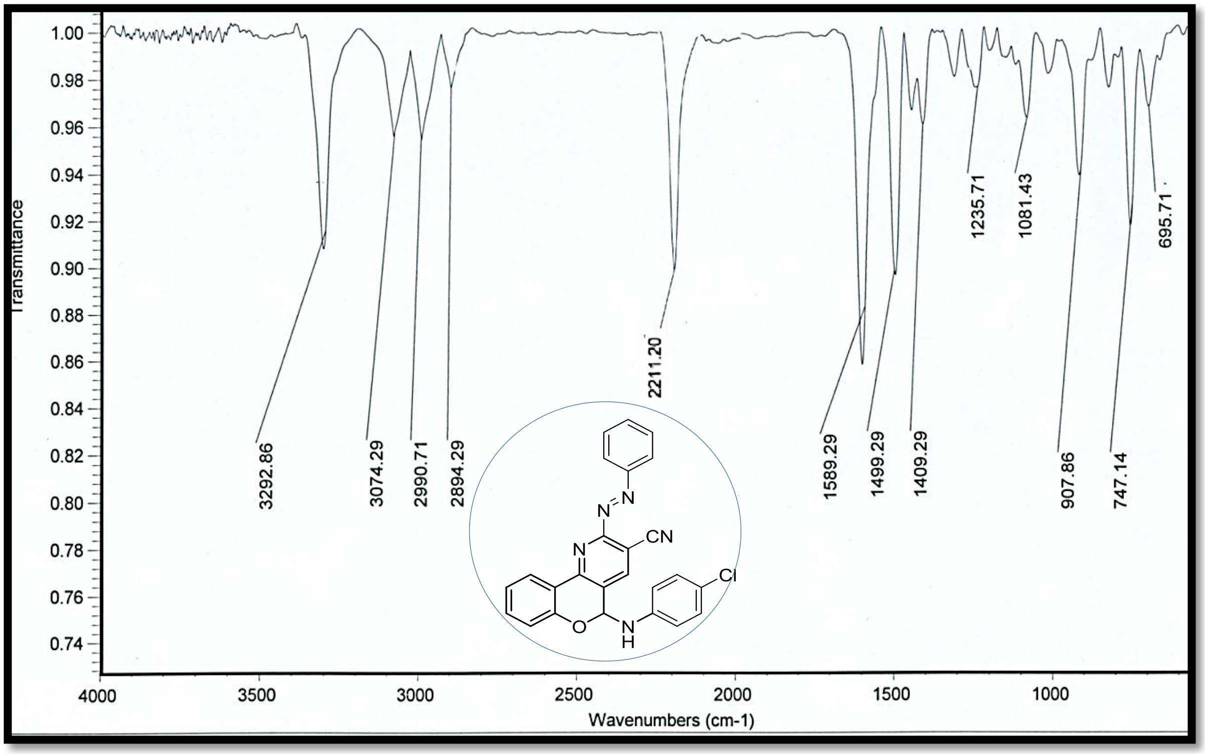

IR (KBr) (**5d**)

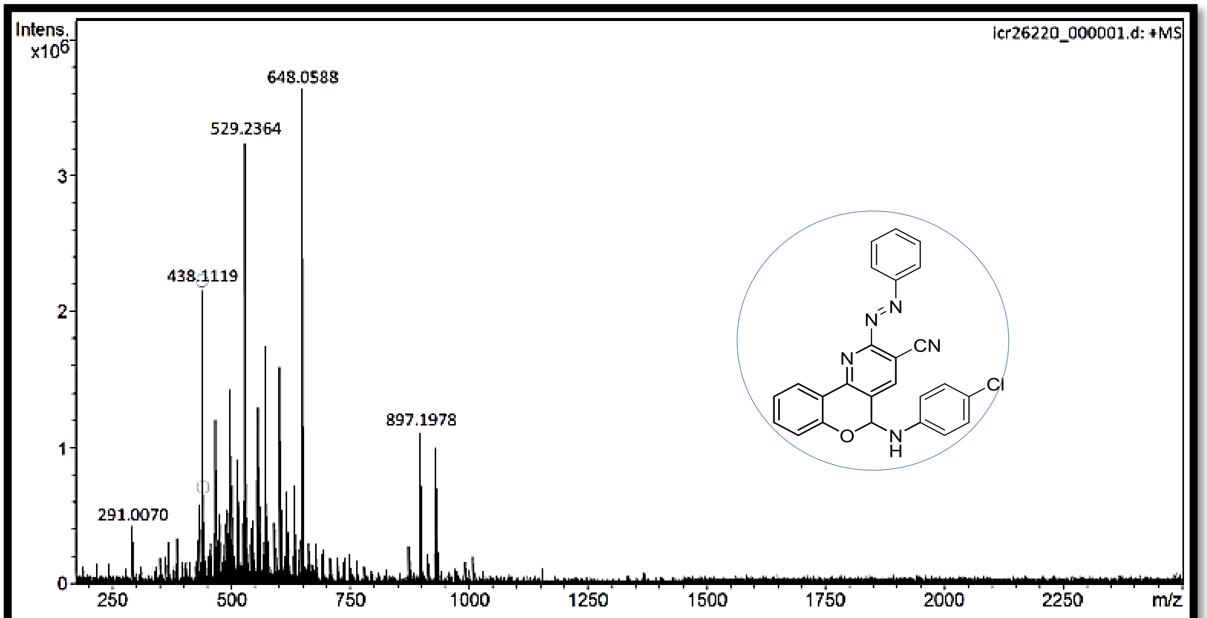

Meas. m/z	Ion Formula	m/z	err [ppm]	mSigma	rdb	e⁻ Conf	N-Rule
438.1119	C18H20N3O10	438.1143	5.6	130.2	10.5	even	ok
	C26H12N7O	438.1098	-4.8	134.9	24.5	even	ok
	C17H14N10O5	438.1143	5.6	136.8	16.0	odd	ok
	C28H14N4O2	438.1111	-1.7	137.2	24.0	odd	ok
	C27H18O6	438.1098	-4.7	148.6	19.0	odd	ok
	C30H16NO3	438.1125	1.4	154.9	23.5	even	ok
440.1089	C33H14NO	440.1070	-4.4	n.a.	27.5	even	ok

HR-Mass (ESI) (**5d**)

Crystallographic data for compound 4a

Table 1: Crystal data and structure refinement for sba141.

Identification code	sba141	
Empirical formula	$C_{28}H_{29}N_5O_3$	
Formula weight	483.56	
Temperature	200(2) K	
Wavelength	0.71073 Å	
Crystal system	monoclinic	
Space group	$P2_1/n$	
Z	4	
Unit cell dimensions	a = 15.586(4) Å	α = 90 deg.
	b =9.519(2) Å	β =111.170(6) deg.
	c = 17.628(4) Å	γ = 90 deg.
Volume	2439.1(10) $Å^3$	
Density (calculated)	1.32 g/cm^3	
Absorption coefficient	0.09 mm^{-1}	
Crystal shape	needle	
Crystal size	0.120 x 0.070 x 0.050 mm^3	
Crystal colour	orange	
Theta range for data collection	1.5 to 20.8 deg.	
Index ranges	$-15 \le h \le 14$, $0 \le k \le 9$, $0 \le l \le 17$	
Reflections collected	10272	
Independent reflections	2624 (R(int) = 0.1111)	
Observed reflections	1199 (I > 2σ(I))	
Absorption correction	Semi-empirical from equivalents	
Max. and min. transmission	0.96 and 0.77	
Refinement method	Full-matrix least-squares on F^2	
Data/restraints/parameters	2624 / 282 / 340	
Goodness-of-fit on F^2	1.00	
Final R indices (I>2sigma(I))	R1 = 0.084, wR2 = 0.183	
Largest diff. peak and hole	0.25 and -0.24 $eÅ^{-3}$	

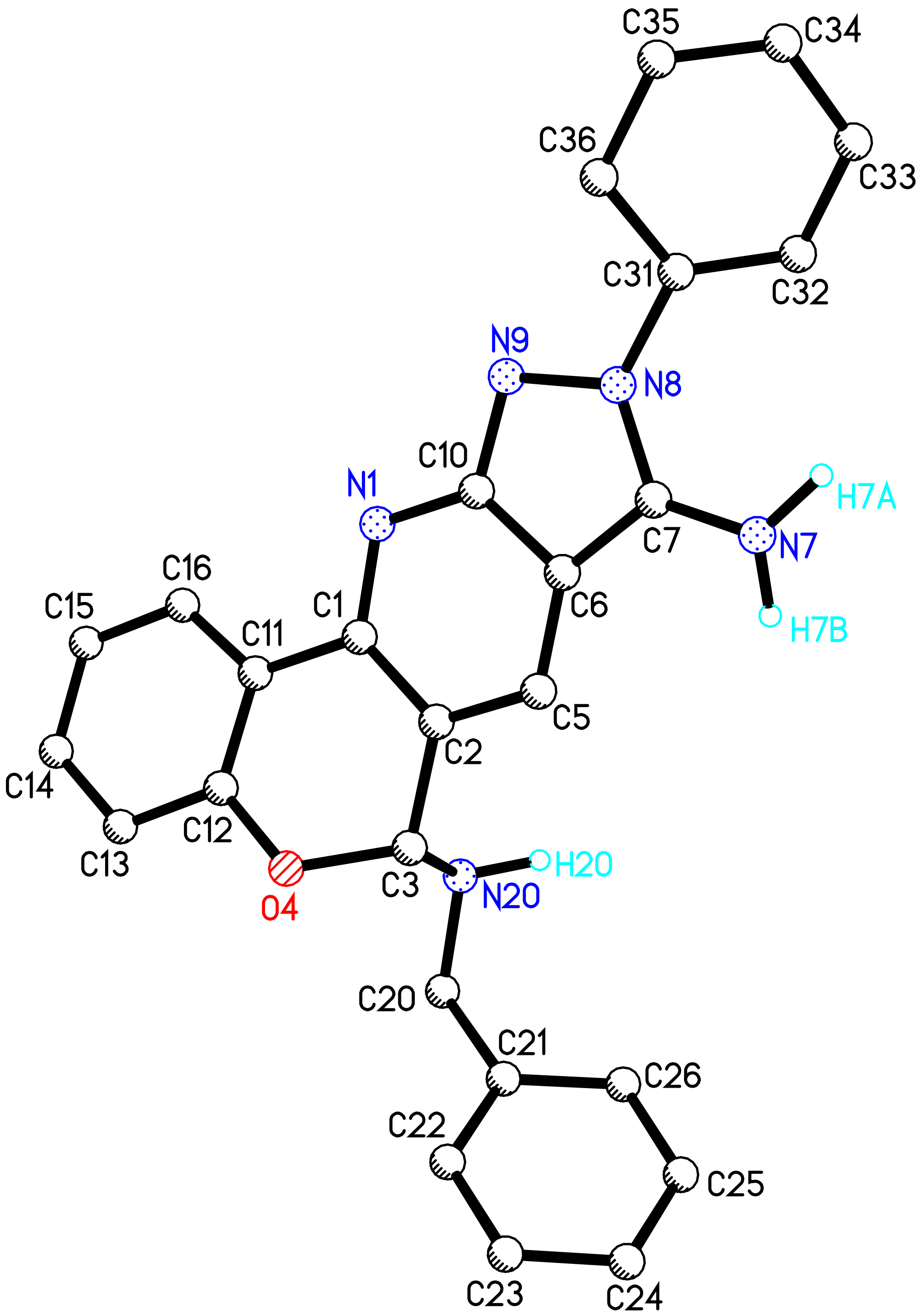
C35
C34
C36
C33
C31
C32
N9
N8
C10
N1
H7A
C7
N7
C16
C6
C15
C1
C11
H7B
C5
C2
C14
C12
C13
O4
C3
H20
N20
C20
C21
C26
C22
C25
C23
C24

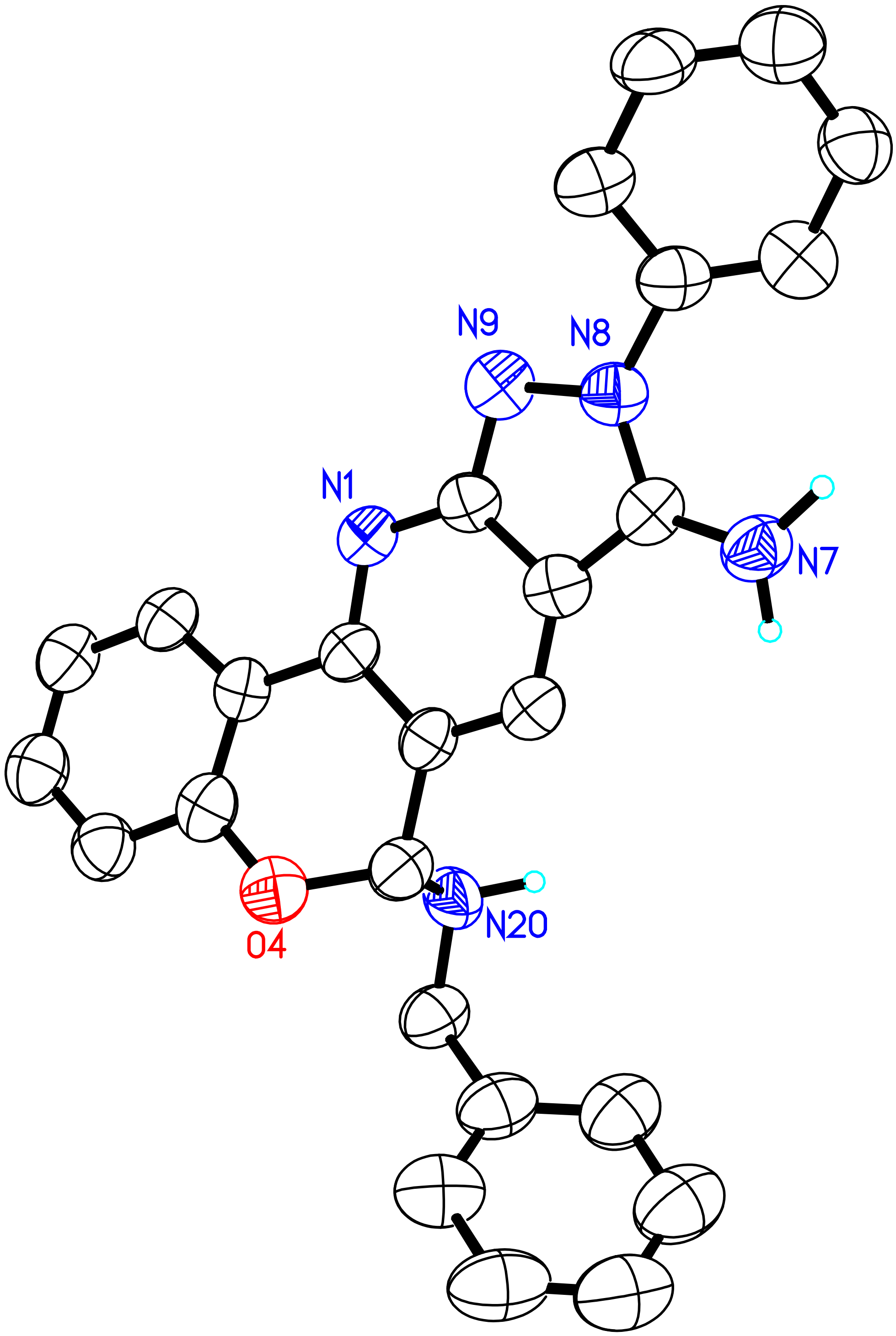
N9
N8
N1
N7
O4
N20

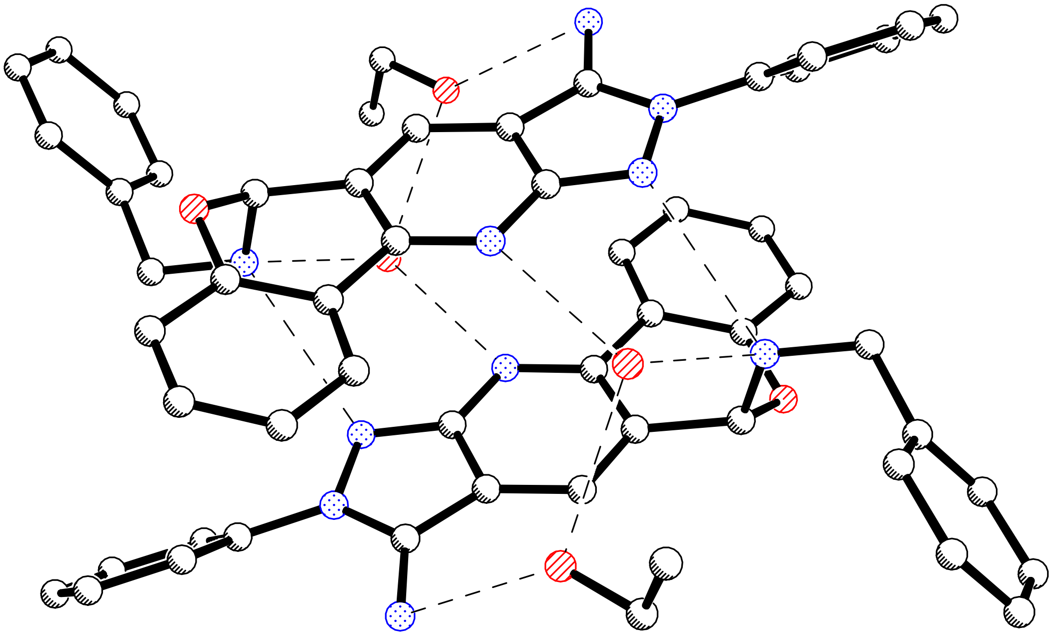

Via hydrogen bridges and the solvent molecules water and ethanol the molecules build pairs with stacking aromatic systems.

References

1. Sheldrick, G. M. Bruker Analytical X-ray-Division, Madison, WI, 2014. (program SADABS 2014/5 for absorption correction)
2. Sheldrick, G. M. *Acta Crystallogr., Sect. C* **2015,** *C71,* 3–8. (program SHELXL-2014/7 (Sheldrick, 2014) for structure refinement) doi:10.1107/S2053229614024218
